# RBM22 regulates RNA polymerase II 5′ pausing, elongation rate, and termination by coordinating 7SK-P-TEFb complex and SPT5

**DOI:** 10.1186/s13059-024-03242-6

**Published:** 2024-04-19

**Authors:** Xian Du, Wenying Qin, Chunyu Yang, Lin Dai, Mingkui San, Yingdan Xia, Siyu Zhou, Mengyang Wang, Shuang Wu, Shaorui Zhang, Huiting Zhou, Fangshu Li, Fang He, Jingfeng Tang, Jia-Yu Chen, Yu Zhou, Rui Xiao

**Affiliations:** 1 Department of Hematology, Medical Research Institute, Frontier Science Center for Immunology and Metabolism, Zhongnan Hospital of Wuhan University, Wuhan University, Wuhan, China; 2 TaiKang Center for Life and Medical Sciences, Wuhan University, Wuhan, China; 3 National “111” Center for Cellular Regulation and Molecular Pharmaceutics, School of Life and Health Sciences, Hubei University of Technology, Wuhan, China; 4 State Key Laboratory of Pharmaceutical Biotechnology, School of Life Sciences, Chemistry and Biomedicine Innovation Center, Nanjing University, Nanjing, China; 5 TaiKang Center for Life and Medical Sciences, College of Life Sciences, State Key Laboratory of Virology, Wuhan University, Wuhan, China

**Keywords:** RBM22, RNA polymerase II, 5′ pausing, Transcription elongation, Transcription termination

## Abstract

**Background:**

Splicing factors are vital for the regulation of RNA splicing, but some have also been implicated in regulating transcription. The underlying molecular mechanisms of their involvement in transcriptional processes remain poorly understood.

**Results:**

Here, we describe a direct role of splicing factor RBM22 in coordinating multiple steps of RNA Polymerase II (RNAPII) transcription in human cells. The RBM22 protein widely occupies the RNAPII-transcribed gene locus in the nucleus. Loss of RBM22 promotes RNAPII pause release, reduces elongation velocity, and provokes transcriptional readthrough genome-wide, coupled with production of transcripts containing sequences from downstream of the gene. RBM22 preferentially binds to the hyperphosphorylated, transcriptionally engaged RNAPII and coordinates its dynamics by regulating the homeostasis of the 7SK-P-TEFb complex and the association between RNAPII and SPT5 at the chromatin level.

**Conclusions:**

Our results uncover the multifaceted role of RBM22 in orchestrating the transcriptional program of RNAPII and provide evidence implicating a splicing factor in both RNAPII elongation kinetics and termination control.

**Supplementary Information:**

The online version contains supplementary material available at 10.1186/s13059-024-03242-6.

## Background

Transcription is a crucial, regulatory phase for gene expression. RNA polymerase II (RNAPII)-mediated transcription involves the promoter recruitment of RNAPII, initiation, promoter-proximal pausing, elongation, and termination, with each step highly regulated [1]. Together with other factors, the DRB sensitivity-inducing factor (DSIF), composed of SPT5 and SPT4, and the negative elongation factor (NELF) complex establish an RNAPII pause [2–4], and such paused RNAPII can be released by the P-TEFb complex, whose CDK9 subunit phosphorylates DSIF, NELF and the Ser2 residues of the RNAPII CTD to enable productive elongation [5, 6]. Alternatively, P-TEFb can be sequestered in a 7SK small nuclear ribonucleoprotein (snRNP)-associated P-TEFb (7SK-P-TEFb) complex with 7SK noncoding RNA and the inhibitor proteins HEXIM1, LARP7 and MePCE [2]. CDK9-mediated phosphorylation leads to the disassociation of NELF from the transcription machinery, while SPT5 is still accompanied by elongating RNAPII, sustaining elongation velocity [7] and efficient termination [8]. At the end of transcription, CPSF-induced cleavage and polyadenylation of nascent RNAs trigger RNAPII termination of most protein-coding genes and lncRNA genes, causing RNAPII to disengage a few kilobases downstream of the polyadenylation (polyA) site in mammalian cells [9, 10].

Splicing factors play a crucial role in facilitating the splicing of pre-mRNA transcripts before mature mRNAs are exported from the nucleus to the cytoplasm [11]. Nonetheless, recent studies have revealed various emerging roles of splicing factors during the transcriptional cycle. For example, SRSF2 facilitates RNAPII pause release [12]; RBM25 mediates transcription factor YY1 function in chromatin binding, DNA looping, and transcription [13]; and RBFOX2 coordinates transcription and polycomb complex 2 (PRC2) at bivalent genes [14]. Intriguingly, the spliceosome component can also be involved in transcription regulation. For instance, U1 snRNP can prevent premature termination during elongation [15], while U2 snRNP is required for RNAPII pause release and elongation velocity at the beginning of genes [16]. These findings have allowed for a better understanding of the overlapping functions of splicing machinery components.

RBM22 is a component of the NineTeen Related (NTR) complex that primarily maintains the conformation of the catalytic core of the spliceosome [17]. During the remodeling B complex to the activated B complex, RBM22 arrives in the complex and contacts the U2 snRNP to maintain it in an opened conformation and stabilize downstream sequences of U6 snRNA [18]. It acts as a bridge between the catalytic core and other essential protein components of the spliceosome, thereby participating in pre-mRNA splicing [19, 20]. Dysregulation of RBM22 is closely associated with cell survival and the progression of cancer [21–23]. Our previous work has revealed the chromatin-binding activity of many RNA-binding proteins (RBPs), including RBM22 [13, 24, 25]. To further investigate whether RBM22 has additional functions independent of its role in splicing, we utilized several top-notch next-generation sequencing (NGS) approaches to analyze its function in multiple regulatory phases of transcription. Our results suggest that RBM22 has a direct role in controlling transcriptional elongation kinetics and termination, thereby regulating gene expression.

## Results

### Splicing factor RBM22 inhibits RNAPII pause release

To understand how RBM22 regulates gene expression, we first analyzed RNA-seq data in RBM22 knockdown HepG2 cells [13], revealing many changes in alternative splicing (AS). Skipped exon and intron retention were frequently induced upon RBM22 knockdown (Additional file 1: Fig. S1a), which were consistent with the known role of RBM22 as a splicing factor. However, the detected changes in gene expression cannot be fully explained by these AS events (Additional file 1: Fig. S1b), suggesting that other possible pathways may contribute to RBM22-dependent gene expression.

**Fig. 1:**
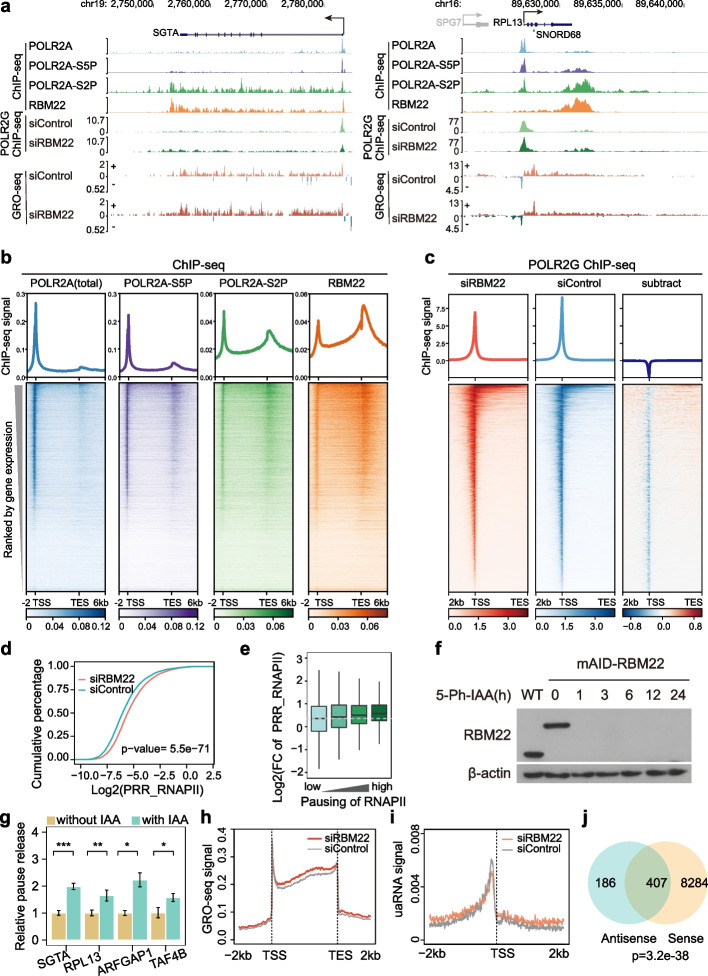
RBM22-mediated repression of RNAPII pause release at many genes. **a** Examples of total RNAPII (POLR2A), Ser5P RNAPII (POLR2A-S5P), Ser2P RNAPII (POLR2A-S2P), and RBM22 occupancy measured by ChIP-seq as well as POLR2G occupancy and gene transcription levels measured by GRO-seq at two representative protein-coding genes (*SGTA* and *RPL13*) in control (siControl) and RBM22 knockdown (siRBM22) HepG2 cells. **b** Heatmap and metagene analysis showing the ChIP-seq signal for POLR2A (total; indigo), POLR2A-S5P (purple), POLR2A-S2P (green), and RBM22 (orange) at all human protein-coding genes in HepG2 cells, rank ordered by gene expression. Color-scaled intensities are in units of cpm. **c** Heatmap and metagene analysis showing the ChIP-seq signal for POLR2G at genes with POLR2G binding at promoters in control and RBM22 knockdown HepG2 cells. Rows are sorted by decreasing POLR2G occupancy in the region between 2 kb upstream of TSS to TES under control condition. For the subtraction of heatmaps, the color bars depict the subtracted values of siRBM22 minus siControl. **d** RNAPII PRR distribution in control or RBM22 knockdown HepG2 cells, showing increased pause release at many genes after RBM22 knockdown. Higher PRR values indicate a higher degree of pause release. The *p* value was determined using the Kolmogorov–Smirnov test. **e** Boxplot showing the fold change (FC) of PRR (POLR2G ChIP-seq) at genes with different degrees of pausing in response to RBM22 depletion. The 9065 genes were equally divided into four groups based on the PRR of RNAPII in the control condition. **f** Western blot showing the protein abundance of RBM22 in wild-type (WT) cells or in mAID-RBM22 HepG2 cells upon the addition of 5-Ph-IAA for the indicated times. β-actin was used as the loading control. The 5-Ph-IAA is an auxin used for degradation. **g** POLR2G ChIP-qPCR quantification of RNAPII pause release at four representative protein-coding genes before (without 5-Ph-IAA) or after (with 5-Ph-IAA) RBM22 degradation in mAID-RBM22 cells. The pause release ratio in untreated cells (without 5-Ph-IAA) was set to 1. The *p* values are determined using the two-tailed unpaired *t*-test (*p ≤ 0.05; **p ≤ 0.01; ns, not significant). **h** Metagene analysis showing the elevated GRO-seq signals at protein-coding genes in RBM22 knockdown HepG2 cells. **i** Metagene analysis of antisense transcription, detected by GRO-seq, at TSS in control and RBM22 knockdown HepG2 cells. **j** Venn diagram showing the protein-coding genes with both PRR changes in the sense direction and transcriptional changes in the antisense direction. The *p* value was determined using the hypergeometric test

Our previous work demonstrated a role of RBM22 in transcriptional control [13]. Indeed, a modest positive correlation was observed between the changes in RNA-seq and GRO-seq signals upon RBM22 depletion (Additional file 1: Fig. S1c), suggesting that RBM22-regulated transcription may directly contribute to mRNA levels. For mechanistic insight into how RBM22 regulates transcription, we first characterized its chromatin occupancy in comparison with that of total, Ser5-phosphorylated (Ser5P) and Ser2-phosphorylated (Ser2P) RNAPII, the latter two of which commonly represent 5′ paused and elongating RNAPII, respectively, in the Encyclopedia of DNA Elements (ENCODE) [26]. As exemplified at the locus of protein-coding genes in HepG2 cells, RBM22 occupancy was observed throughout the entire gene (Fig. 1a). The genome-wide occupancy of RBM22 at genes had striking similarities with the profile of Ser2P RNAPII, with maximal occupancy levels at the promoter-proximal region and downstream of the polyA sites (also referred to here as TES, for transcription end site) (Fig. 1b), where RNAPII frequently pauses for the 5′ elongation checkpoint and 3′ termination, respectively [2]. Additionally, RBM22 showed stronger chromatin occupancy on genes with higher levels of gene expression (Fig. 1b). To comprehensively characterize RBM22 occupancy in the genome, we investigated its presence on snoRNA and snRNA genes, revealing evident but distinct signals (Additional file 1: Fig. S1d). A similar chromatin association of RBM22 was also observed in K562 cells (Additional file 1: Fig. S1d, e). These data suggest that RBM22 might regulate transcription at multiple phases.

Initially, we evaluated the expression and splicing changes (Additional file 1: Fig. S1a, b) of established regulatory transcription factors [2, 27–33] following the RBM22 knockdown. Our results indicate no significant alternations in the expression level and splicing patterns of these factors, thereby excluding the possibility of an indirect effect (Additional file 1: Fig. S1f, g, and h). We next examined the impact of RBM22 on RNAPII occupancy. To monitor total RNAPII, we used a well-characterized antibody against the RNAPII subunit POLR2G [34, 35], which has the same capacity to detect RNAPII-chromatin interactions as the anti-POLR2A antibody [13], to perform ChIP-seq and obtained high-quality and reproducible data (Fig. 1c, Additional file 1: Fig. S2a, b). We verified positive correlations between POLR2G and RBM22 density at the transcription start site (TSS), gene body, and TES (Additional file 1: Fig. S2c). Upon RBM22 depletion, RNAPII levels over transcription start sites (TSSs) were clearly decreased, coupled with an increased density of RNAPII in gene bodies, as evident from both single gene profiles and metagene analysis of protein-coding genes (Fig. 1a, c), suggesting a genome-wide role of RBM22 in controlling RNAPII pausing and release at promoter-proximal regions. To further quantify such changes in RNAPII occupancy, we analyzed the modified “pause release ratio” (PRR) of RNAPII on individual genes [36], that is, the ratio of RNAPII occupancy between the gene body (+ 500 bp from TSS to − 500 bp from TES) and its promoter (− 300 to + 100 bp from TSS) (Additional file 1: Fig. S2d). Consistently, a global increase in the RNAPII PRR in response to RBM22 depletion was detected (Fig. 1d), and 3960 genes displayed a ≥ 1.5-fold increase (Additional file 1: Fig. S2e and Additional file 2: Table S1), confirming that RBM22 represses RNAPII pause release at promoters. Moreover, such effects can be rescued by re-expression of the siRNA-resistant RBM22 (Additional file 1: Fig. S2f, g). Additionally, we performed a Gene Ontology (GO) analysis for genes whose pausing is strongly regulated by RBM22. The analysis revealed enrichment in various biological processes, including cellular component disassembly, proteasome-mediated ubiquitin-dependent protein catabolic process, vesicle organization, regulation of autophagy, macroautophagy, Golgi vesicle transport, and more (Additional file 1: Fig. S2h). Furthermore, we analyzed the degree of this regulatory effect of RBM22 on the genes of different pausing-and-release states of RNAPII, and the results showed a stronger effect on the genes with higher pausing compared with those with lower pausing (Fig. 1e). Notably, this effect was not observed on genes with higher expression levels (Additional file 1: Fig. S2i). Interestingly, RBM22 exhibits an opposing effect to chromatin-associated splicing factors SRSF1 and SRSF2 on the release of RNAPII pausing [12]. To eliminate the possibility of secondary effects resulting from chronic RBM22 depletion via RNAi, we employed the auxin-inducible degron (AID) system [37, 38]. This system allowed us to achieve a rapid and acute depletion of RBM22 by introducing a mini-AID (mAID) tag at the N-terminus of the *RBM22* gene in OsTir1 (F74G)-expressing HepG2 cells. As expected, the addition of 5-Ph-IAA robustly induced the rapid depletion of RBM22 protein within 1 h in mAID-RBM22 cells (Fig. 1f) while leaving the protein levels of RBM22 and POLR2G unaltered in WT HepG2 cells (Additional file 1: Fig. S2j). We next used anti-RBM22 ChIP-qPCR to confirm that mAID-tagged RBM22 maintains a specific binding pattern on chromatin similar to that of the endogenous RBM22 (Additional file 1: Fig. S2k). Consistently, we obtained similar results in terms of RNAPII pause release at the *SGTA*, *RPL13*, *ARFGAP1*, and *TAF4B* genes upon acute depletion (Fig. 1g), while observing no such changes in WT HepG2 cells (Additional file 1: Fig. S2l), indicating a direct effect.

Given that RNAPII pausing and release appear to be affected by splicing inhibition [39, 40] and that the depletion of RBM22 inhibits the first step of splicing [41, 42], it is possible that RBM22 may regulate pause release through affecting splicing. Pladienolide B (Pla-B) appears to have similar efficacy in inhibiting the first step of splicing by blocking SF3B1 [43, 44]. Nevertheless, Pla-B-mediated splicing inhibition globally increases RNAPII pause duration near the promoter [16], displaying an opposite effect of RBM22. This effect was corroborated through SF3B1 knockdown (Additional file 1: Fig. S2m, n). Additionally, no significant difference was observed in the RNAPII PRR levels between intron-less genes and genes bearing introns (Additional file 1: Fig. S2o). Thus, the effect of RBM22 on RNAPII pausing and release is improbable due to affecting splicing. Taken together, these results suggest that the chromatin association of RBM22 mediates the suppression of the pause release of RNAPII at many gene promoters.

### RBM22 depletion enhances both sense and promoter upstream antisense RNA synthesis

To determine whether RNAPII pause release provoked by RBM22 depletion in turn promotes RNA synthesis, we analyzed global nuclear run-on coupled with deep sequencing (GRO-seq) data which reflect nascent RNA synthesis by transcriptionally engaged RNAPII, upon RBP depletion [13]. As expected, RBM22 knockdown resulted in a significant increase in GRO-seq signals in the gene body regions; in contrast, the knockdown of another chromatin-associated splicing factor, U2AF2, showed no such effect (Fig. 1h, Additional file 1: Fig. S2p). Additionally, a significant increase in the level of PRR of GRO-seq signals was detected upon RBM22 depletion but not U2AF2 depletion (Additional file 1: Fig. S2q). These data further confirm the inhibitory role of RBM22 in RNAPII pause release and emphasize the contribution of its chromatin association on such a role.

Interestingly, transcription elongation in the antisense direction at the promoter, which produces promoter upstream antisense RNA (uaRNAs or PROMPTs) [45–47], was also apparently elevated in RBM22-depleted cells, as illustrated at the locus of *SGTA* and *RPL13* genes (Fig. 1a). Moreover, metagene analysis shows that the level of uaRNAs is evidently increased beyond 300 bp upstream of the TSS, with a clear decrease near the TSS in RBM22-depleted cells; however, U2AF2 depletion has no such effect genome-wide (Fig. 1i, Additional file 1: Fig. S2r). We confirmed these changes with simplified transient transcriptome [10] quantitative PCR (TT-qPCR) experiments at example genes as well as many other genes in the cells treated with two independent *RBM22*-targeted siRNAs (Additional file 1: Fig. S2s; Method Details). These results were consistent with elevated RNAPII occupancy upstream of gene promoters in response to RBM22 depletion (Fig. 1c). Notably, 68% of these changes in uaRNAs transcription overlapped with the changes in the PRRs of associated genes in the sense direction (Fig. 1j), suggesting that pause release induced by RBM22 depletion at gene promoters in the sense and antisense direction are functionally coupled. Collectively, these results support the idea that RBM22 controls bidirectional elongation by RNAPII.

### RBM22 is required to maintain rapid elongation of RNAPII across genes

**Fig. 2:**
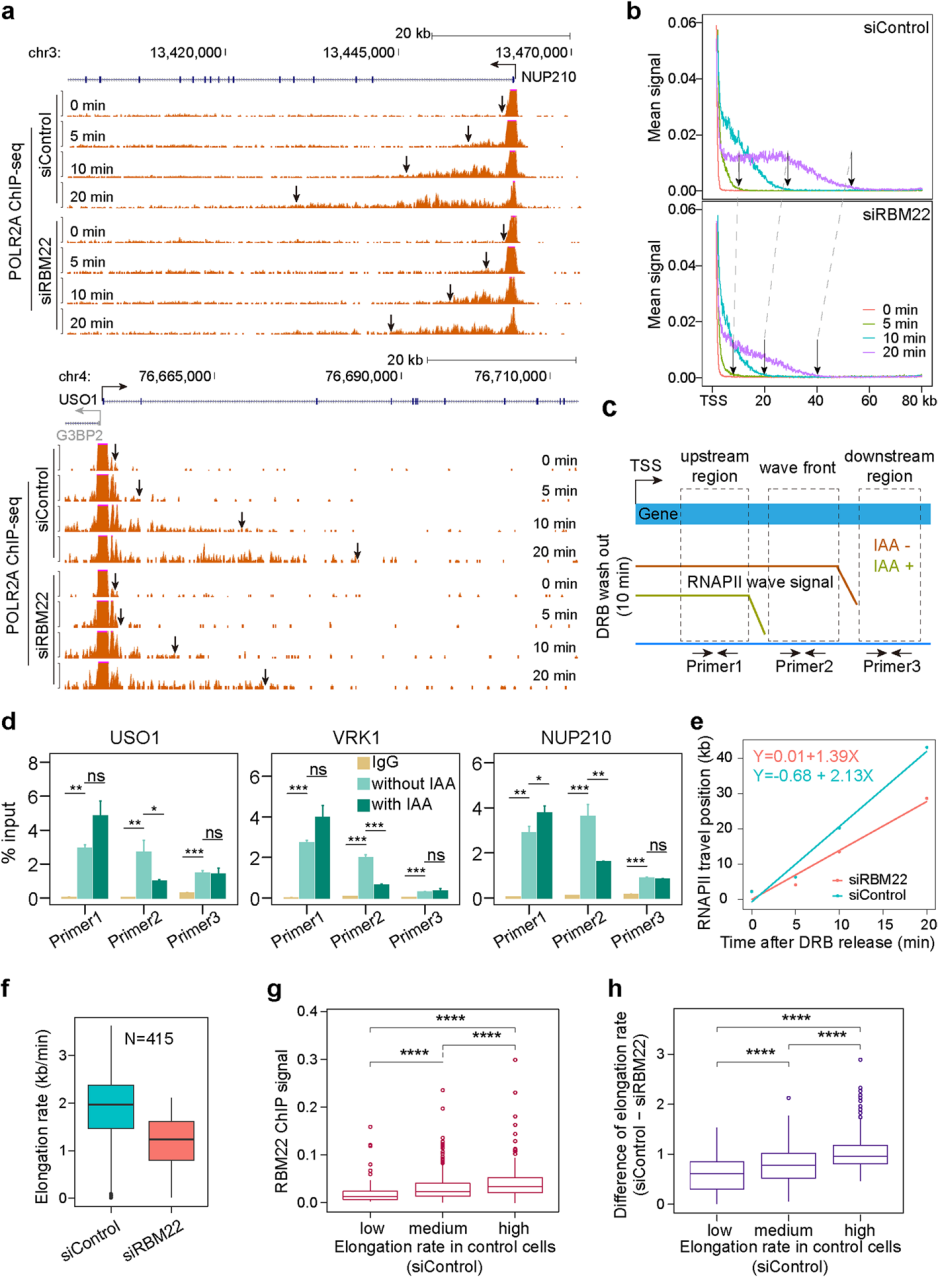
DRB/POLR2A ChIP-Seq measures RNAPII elongation rates across genes. **a** Examples of DRB/POLR2A ChIP-seq signals at two representative genes (*NUP210* and *USO1*) at different time points following the release from DRB-induced inhibition of transcriptional elongation. Arrows indicate the front of the transcription wave. **b** DRB/POLR2A ChIP-seq metagene profiles for the genes longer than 80 kb (*N* = 2217). Arrows indicate the front of the calculated transcription wave. **c** Schematic representation of the regions amplified in the ChIP-qPCR. The wave front represents the region where RNAPII transcribes only in the condition without 5-Ph-IAA treatment. The upstream region represents the common interval where RNAPII transcribes with or without 5-Ph-IAA treatment. The downstream region represents the interval where RNAPII does not transcribe with or without 5-Ph-IAA treatment. **d** POLR2A ChIP-qPCR showing the RNAPII enrichment in different areas of example long genes in mAID-RBM22 cells with 5-Ph-IAA or without 5-Ph-IAA treatment. Values are mean ± SD (*n* = 4). The *p* values are determined using the two-tailed unpaired *t*-test (*p ≤ 0.05; **p ≤ 0.01; ***p ≤ 0.001; ****p < 0.00001; ns, not significant). **e** Calculation of the RNAPII average elongation rates based on metagene profiles using linear regression for all the genes longer than 80 kb with high POLR2A ChIP-seq signals in control (2.13 kb/min) or RBM22 knockdown (1.39 kb/min) HepG2 cells. **f** Boxplot analysis of the RNAPII elongation rates for individual 415 genes with robust signal at all time points. **g–h** Boxplot analysis of the RBM22 ChIP-seq signals (**g**) and the elongation rate changes upon RBM22 knockdown (**h**) for the genes with different elongation rates in control cells. The 598 genes with computable elongation rates were divided into three groups based on elongation rate in control cells: low (rate < 1.87 kb/min), medium (1.87 kb/min < rate < 2.36 kb/min), and high (rate > 2.36 kb/min). The *p* values are determined using the two-tailed unpaired *t*-test (*p ≤ 0.05; **p ≤ 0.01; ***p ≤ 0.001; ****p < 0.00001)

Given the RBM22 occupancy throughout the entire gene, we next examined whether it might play a role in regulating the RNAPII elongation rate. The reversible CDK9 inhibitor 5,6-dichloro-1-b-D-ribofurano-sylbenzimidazole (DRB), which can arrest RNAPII at the TSS of genes while permitting it to clear from gene bodies, was used to measure the elongation rate. The paused polymerases could be released into the gene body in a synchronous ‘‘wave” after removing DRB [48]. We monitored the RNAPII wave at 0, 5, 10, and 20 min after DRB washout by anti-POLR2A ChIP-seq (Additional file 1: Fig. S3a, b, c). At each time point after DRB removal, we observed that the RNAPII elongation wave was visibly delayed in the RBM22-depleted cells, as shown in both single gene and metagene profile analysis (Fig. 2a, b), suggesting that RNAPII transcribes slower in the absence of RBM22. To confirm these effects, we performed anti-POLR2A ChIP-qPCR, which enabled us to examine the RNAPII wave front signal and reevaluate the elongation rate in mAID-RBM22 cells (Fig. 2c). As anticipated, a significant reduction in the signal from elongating RNAPII was observed in the region of the RNAPII wave front at *NUP210* and *USO1* genes 10 min after DRB washout, following the acute depletion of RBM22. In contrast, no such changes were detected in the upstream and downstream regions (Fig. 2d), thereby confirming a decrease in the elongation rate. Subsequently, we computed the average POLR2A ChIP-seq signals from the 0, 5, 10, and 20-min samples across all genes longer than 80 kb (2217 genes) and used linear regression to calculate the RNAPII elongation rates. The average elongation rate of RNAPII across genes was apparently decreased from 2.13 kb/min, as reported in previous studies [49, 50], to 1.39 kb/min after RBM22 knockdown (Fig. 2e). Such decreased elongation rate can be readily repeated by another independent experiment (Additional file 1: Fig. S3d, e). Moreover, to compare the RNAPII elongation rate at the individual gene level, we deliberately selected 415 genes exhibiting robust RNAPII ChIP-seq signals at all three time points in both control and knockdown cells, ensuring precision in our calculation. Consistently, a globally reduced elongation rate was observed (Fig. 2f). These results demonstrate that RBM22 is crucial for promoting fast elongation by RNAPII genome-wide, exhibiting an additional role of RBM22 in transcriptional elongation kinetics.

To further investigate the impact of RBM22 occupancy on elongation velocity, we split genes into three subsets based on elongation velocity in control cells and found that the genes of faster transcriptional elongation showed higher levels of RBM22 occupancy at genes (Fig. 2g), indicating a positive correlation between elongation velocity and RBM22 occupancy. Accordingly, the genes with higher transcription elongation velocity displayed a greater decrease than others after silencing *RBM22* (Fig. 2h). In contrast, the RBM22 occupancy is uncorrelated with gene length (Additional file 1: Fig. S3f), meanwhile, the changes in RNAPII elongation rate were relatively unaffected by the increases in gene length (Additional file 1: Fig. S3g). These results suggest that a contribution of the chromatin association of RBM22 to transcription elongation velocity.

Pla-B-mediated splicing inhibition also leads to a reduction in RNAPII elongation velocity at the beginning of many genes [39], in contrast, our results demonstrated that RBM22 promotes elongation velocity across the entire genes, showing a distinct effect. Additionally, the changes in RNAPII elongation rate were relatively unaffected by the increases in intron density (Additional file 1: Fig. S3h). Thus, these data suggest that RBM22-mediated regulation on elongation velocity is unlikely due to affecting splicing, although we cannot completely rule out the effect of splicing.

### Loss of RBM22 impairs transcription termination of protein-coding genes

**Fig. 3:**
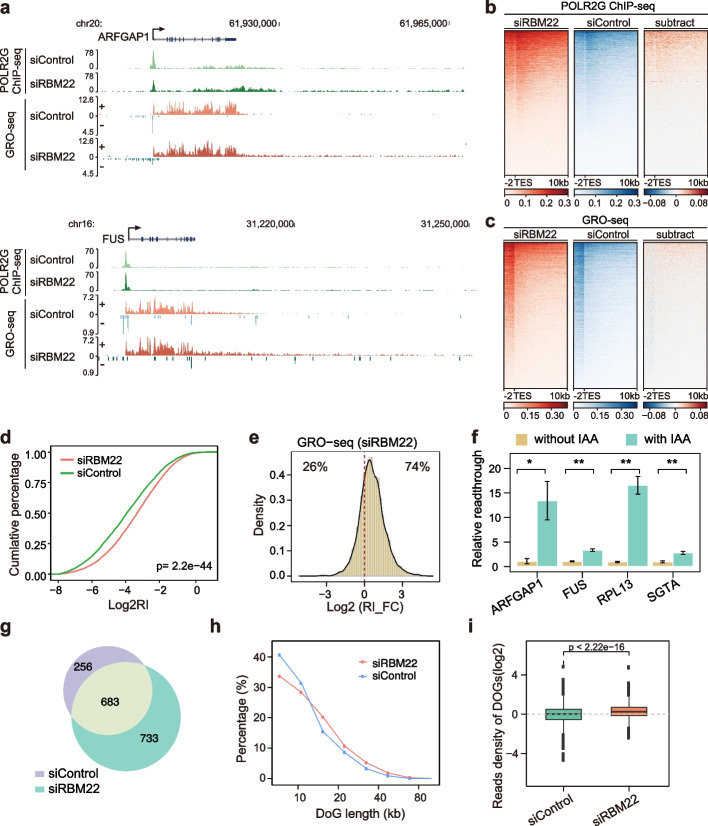
RBM22 controls the transcription termination of protein-coding genes. **a** Examples of POLR2G ChIP-seq and GRO-seq signals at two representative protein-coding genes (*ARFGAP1* and *FUS*) in control, RBM22 knockdown HepG2 cells, showing the enhanced transcriptional readthrough in the absence of RBM22. **b–c** Heatmaps displaying the changes in POLR2G ChIP-seq (**b**) and GRO-seq (**c**) signals in a region from 2 kb upstream to 10 kb downstream of the TES of each protein-coding gene (*N* = 20,003) upon RBM22 knockdown in HepG2 cells. For the subtraction of heatmaps, the color bars depict the subtracted values of siRBM22 minus siControl. **d** RI distribution in control and RBM22 knockdown HepG2 cells, showing the enhanced transcriptional readthrough at many transcribed genes (*N* = 5626) upon RBM22 knockdown. The *p* value was determined using the Kolmogorov–Smirnov test. **e** Histogram showing the fold change (FC) of RI (GRO-seq) at protein-coding genes upon RBM22 knockdown in HepG2 cells. **f** TT-qPCR quantification of transcriptional readthrough at four representative protein-coding genes before (without 5-Ph-IAA) or after (with 5-Ph-IAA) RBM22 degradation in mAID-RBM22 cells. The readthrough ratio in untreated cells (without 5-Ph-IAA) was set to 1. Error bars represent the SD. The *p* values are determined using the two-tailed unpaired *t*-test (*p ≤ 0.05; **p ≤ 0.01; ns, not significant). **g** Venn diagram of DoGs discovered in the two individual conditions. **h** Length distribution of DoGs discovered in the three individual conditions. The percentage of DoGs of various lengths relative to the entire set of DoGs identified in individual conditions is shown on the *y*-axis. **i** Boxplot showing the GRO-seq signal at DoG regions in the two conditions

The high occupancy of RBM22 at RNAPII pausing sites in the region downstream of the TES (Fig. 1b) implies that RBM22 might have a role in RNAPII termination. Thus, we next examined RNAPII occupancy beyond the TES. Interestingly, a strong increase in total RNAPII levels downstream of the TES was detected in RBM22-depleted cells (Fig. 3a, b), suggesting that RNAPII continues elongation even after reaching the TES and that transcriptional readthrough may occur.

To determine whether RBM22 depletion causes transcriptional readthrough, we analyzed the GRO-seq signals beyond the TES. Consistent with the elevated RNAPII occupancy downstream of the TES, the loss of RBM22 indeed induced substantial readthrough at many actively transcribed genes (Fig. 3a, c). We confirmed the changes in readthrough frequency in our example genes and in many other genes by TT-qPCR and these changes could be rescued by the re-expression of RBM22 (Additional file 1: Fig. S4a). To quantify the changes in the transcriptional readthrough of individual genes, the readthrough index (RI), the ratio of length-normalized nascent RNA levels in the region beyond the TES to the levels in the last exon [51], was analyzed genome-wide (Additional file 1: Fig. S4b). As expected, the RI values of most actively transcribed genes were significantly increased in RBM22-depleted cells (Fig. 3d), and 3260 genes displayed a ≥ 1.5-fold increase in RI (Fig. 3e, Additional file 2: Table S2). In comparison, the RI was not changed in U2AF2-depleted cells (Additional file 1: Fig. S4c). Furthermore, acute depletion of RBM22 in cells consistently recapitulates the alterations in transcription termination observed at *ARFGAP1*, *FUS*, *RPL13*, and *SGTA* genes (Fig. 3f). Taken together, these data indicate that chromatin-associated RBM22 is required for transcription termination at protein-coding genes.

Given that the inefficient splicing of terminal introns could potentially result in transcriptional readthrough [52–55], we analyzed how this effect contributes to the termination defect upon RBM22 depletion. However, only 2.70% of readthrough genes were associated with retained terminal introns, showing a subtle contribution (Additional file 1: Fig. S4d). We also confirmed that the splicing inhibition by Pla-B did not result in widespread transcriptional readthrough of protein-coding genes (Additional file 1: Fig. S4e) by analyzing a previously published TT-seq dataset [16]. Thus, it is unlikely that the global effects of RBM22 knockdown on termination are due to the effects on terminal intron splicing or global splicing inhibition, suggesting a splicing-independent effect at the vast majority of regulated genes.

Stress-induced readthrough transcription results in the synthesis of downstream-of-gene (DoG)-containing transcripts, a unique class of noncoding RNAs [56], that might affect downstream gene expression [53]. To evaluate DoG transcripts, we employed ARTDeco [57] to identify DoG regions genome-wide. By definition, normal transcription termination events occur on average approximately 4 kb downstream of annotated gene ends [10]. We observed an increase in the number of DoG regions (> 5 kb) from 939 in the control cells to 1416 in the RBM22-depleted cells, with 256 regions lost and 733 new regions gained (Fig. 3g). In addition to the enlarged size of the DoG regions (Fig. 3h), an increased expression of DoG transcripts was also detected upon RBM22 depletion (Fig. 3i).

Furthermore, in our analysis of RNA-seq data, we found that the expression of a small proportion of downstream genes was altered, showing a minor effect of readthrough induced by RBM22 on downstream gene expression (Additional file 1: Fig. S4f). A likely explanation was that without intruding into the neighboring gene locus (Additional file 1: Fig. S4g), the DoG transcripts provoked by RBM22 depletion are unable to interfere with the expression of downstream genes. Together, these data indicate that RBM22 loss-of-function causes DoG transcript expression but does not affect downstream gene expression.

### RBM22 controls the transcription of snoRNA and snRNA

**Fig. 4:**
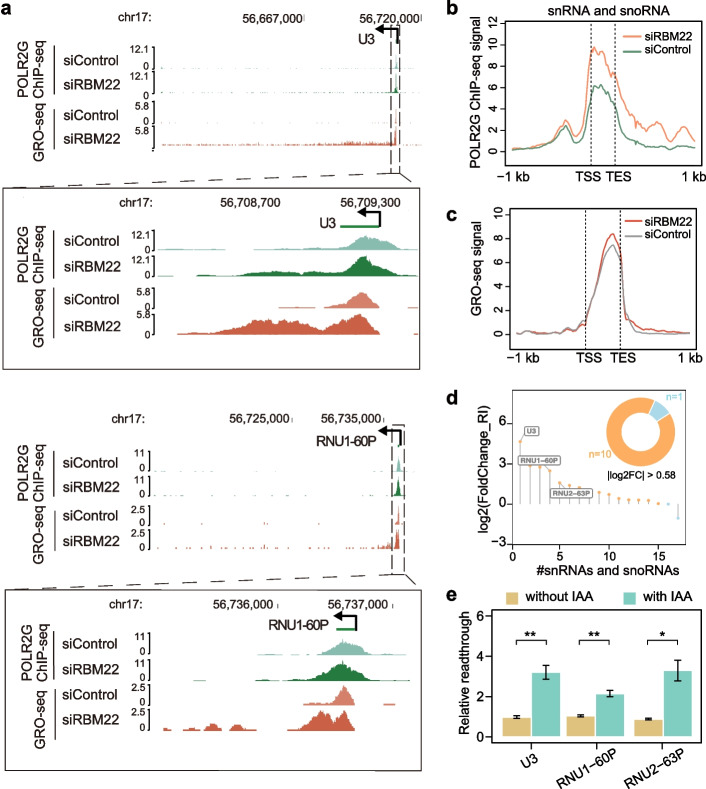
RBM22-mediated transcriptional control at snoRNA and snRNA genes. **a** Examples of POLR2G ChIP-seq and GRO-seq signal at two representative sno/snRNA genes (*U3* and *RNU1-60P*) in control and RBM22 knockdown HepG2 cells, showing the enhanced transcriptional readthrough induced by RBM22 knockdown. **b** Metagene analysis showing the change in POLR2G ChIP-seq signal at independently transcribed snoRNA and snRNA genes (*N* = 25) upon RBM22 knockdown. **c** Metagene analysis showing the change in GRO-seq signal at independently transcribed snoRNA and snRNA genes (*N* = 25) upon RBM22 knockdown. **d** Histogram showing the fold change of RI value for sno/snRNA genes upon RBM22 knockdown, ranked according to RI value. Doughnut plot displaying the number of genes with significant RI change, determined by |log2FC|> 0.58 (*n* = 11). SnoRNA/snRNA genes occupied by POLR2G in the control condition, as defined by having a peak called MACS2 and not overlapped with transcribed genes were used (*n* = 17). **e** TT-qPCR quantification of transcriptional readthrough at three representative sno/snRNA genes before (without 5-Ph-IAA) or after (with 5-Ph-IAA) RBM22 degradation in mAID-RBM22 cells. The readthrough ratio in untreated cells (without 5-Ph-IAA) was set to 1. Error bars represent the SD. The *p* values are determined using the two-tailed unpaired *t*-test (*p ≤ 0.05; **p ≤ 0.01; ns, not significant)

In addition to protein-coding genes, RNAPII-transcribed snRNA and snoRNA genes are also obviously occupied by RBM22 (Additional file 1: Fig. S1e), suggesting that RBM22 might affect their transcription. For a subset of independently transcribed snoRNAs and snRNAs, a strong increase in RNAPII occupancy in both genic regions and downstream regions of TES was detected in the RBM22-depleted cells (Fig. 4a, b), displaying stronger recruitment of RNAPII and continuous transcription without disengagement beyond TES. Consistently, we observed a significant increase in nascent RNA signals up to 100 kb downstream of the TES, in addition to increased nascent RNA signals in genic regions in the absence of RBM22 (Fig. 4a, c), confirming the stronger transcriptional activity and compromised termination. In contrast, no such changes were detected in the U2AF2-depleted cells (Additional file 1: Fig. S5a). We validated these changes by TT-qPCR, which also could be rescued by re-expression of RBM22 (Additional file 1: Fig. S5b). To quantify the level of transcriptional readthrough, we specifically defined the RI values for snoRNAs and snRNAs (Additional file 1: Fig. S5c). Remarkably, the RI values of the vast majority of these small noncoding RNAs were dramatically increased (Fig. 4d, Additional file 2: Table S3). Moreover, we verified the changes in transcription termination at *U3*, *RNU1-60P*, and *RNU2-63P* genes upon the acute depletion of RBM22 (Fig. 4e). These data indicate that in addition to its effects on protein-coding genes, RBM22 also restrains the transcriptional activity of snoRNA and snRNA genes and facilitates their termination. Taken together, these results demonstrate a common effect of RBM22 on transcriptional activity and termination at diverse classes of RNAPII-transcribed genes.

It is worth noting that these snoRNA and snRNA genes are independently transcribed and do not require splicing. Moreover, subtle changes in transcriptional activity and read-through were detected at these types of genes by TT-seq upon splicing inhibition [39] (Additional file 1: Fig. S5d, e). Collectively, these results suggest a splicing-independent role of RBM22 on the termination of this subset of snoRNA and snRNA genes.

### sno/snRNA gene-derived DoGs are provoked by RBM22 depletion

Intriguingly, in contrast to previously characterized readthrough transcripts that typically initiate from protein-coding genes [53], the readthrough transcription at sno/snRNA genes may generate a new type of DoG, sno/snRNA gene-derived DoGs (sRDoGs) (Fig. 4a). To determine whether these sRDoGs are polyadenylated, we used modified, circulation-based 3′ sequencing (c3′-seq) to quantify the genome-wide usage of polyA sites **(**Additional file 1: Fig. S5f; Method Details). Confirming the quality of the c3′-seq data, the majority of detected signals were located at the end of annotated genes with high reproducibility (Additional file 1: Fig. S5g, h, i), and canonical polyadenylation signals, such as AWTAAA and 2GT/T elements, were substantially enriched around the end of c3′-seq reads (Additional file 1: Fig. S5j). However, we were not able to detect confident polyA signals in these sRDoG regions, although each region contains more than one AWTAAA element (Additional file 1: Fig. S5k), suggesting that unlike those protein-coding gene-derived DoGs upon osmotic stress [58], these sRDoGs induced by RBM22 depletion do not undergo polyadenylation. These transcripts may be unstable and subjected to quick degradation by nuclear quality control.

### RBM22 is associated with 5′ paused and elongating RNAPII and inhibitory 7SK-P-TEFb complex

**Fig. 5:**
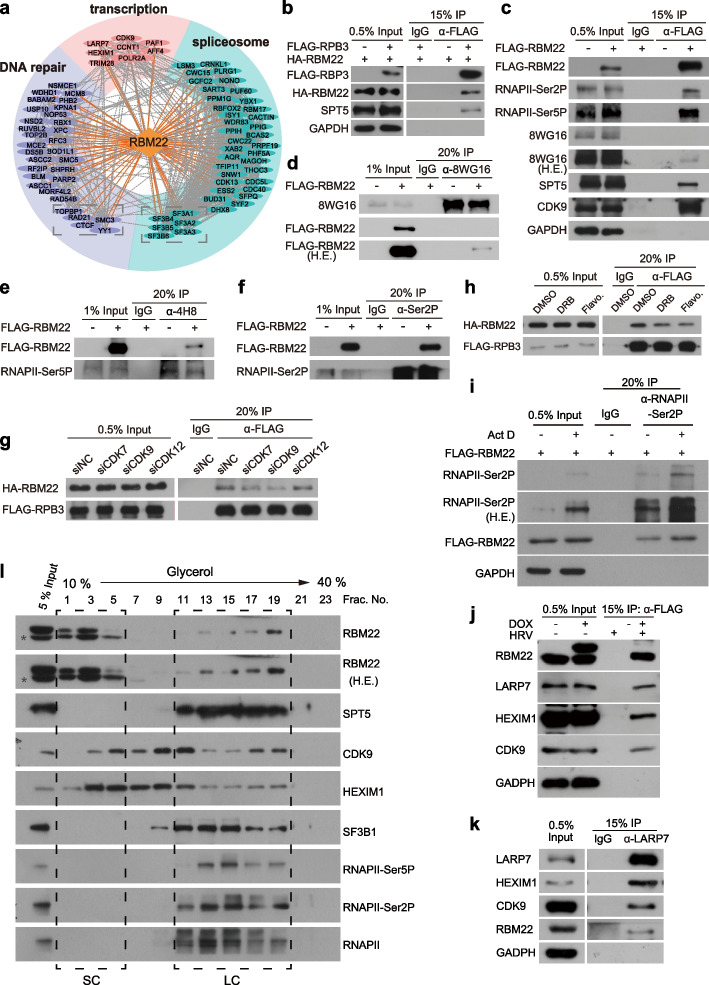
Interaction network of RBM22 between RNAPII and inhibitory 7SK-P-TEFb complex. **a** Cytoscape network analysis of the RBM22 protein interactome in HepG2 cells. Orange diamond, RBM22; light blue oval, spliceosome complex; pink oval, transcription; purple oval, DNA repair. **b** Western blot results showing co-immunoprecipitation of HA-tagged RBM22 and FLAG-tagged RPB3 in HEK293T cells. **c** Western blot results showing co-immunoprecipitation of FLAG-tagged RBM22 and RNAPII with different phosphorylation status, SPT5, CDK9 in HepG2 cells. **d–f** Reciprocal co-immunoprecipitation results showing the distinct interaction between RBM22 and unphosphorylated (**d**), Ser5P (**e**), and Ser2P (**f**) RNAPII in HepG2 cells. **g** Effect of CDK7, CDK9, and CDK12 knockdown on the interaction between RBM22 and RPB3 in HepG2 cells. **h** Effect of DRB and flavopiridol treatment on the interaction between RBM22 and RPB3 in HepG2 cells. **i** Effect of Actinomycin D treatment on the interaction between RBM22 and RNAPII in HepG2 cells. **j–k** Reciprocal co-IP results in HepG2 cells inducibly expressing FLAG-HRV-tagged RBM22 (**j**) and wild-type HepG2 cells (**k**) showing the association between RBM22 and 7SK-P-TEFb complex. **l** RBM22 co-fractionation with RNAPII and spliceosome and identification of separate RBM22-7SK complex. HepG2 cell lysate was analyzed by glycerol gradient sedimentation. Collected fractions were detected by Western blotting. The dashed box highlights a large complex (LC) and a small complex (SC) associated with RBM22. The asterisk indicates a non-specific signal

The data presented above indicate that RBM22 is an important transcriptional regulator of RNAPII, but the underlying molecular mechanism is unclear. We therefore next sought to study the protein interactome of RBM22. Cells inducibly expressing FLAG-HRV-tagged RBM22 were lysed, and RBM22-interacting proteins were isolated by FLAG immunoprecipitation, specifically released by HRV 3C proteinase and analyzed by quantitative label-free mass spectrometry (qMS; Additional file 1: Fig. S6a). Relative to control cells without Dox induction, this analysis identified 517 interacting partners (Fig. 5a, Additional file 2: Table S4), showing enrichment for spliceosome components, especially U2 snRNP, consistent with the role of RBM22 as a splicing factor [20, 59]. Importantly, we also identified many transcription elongation factors, such as P-TEFb (consisting of CDK9 and CCNT1), PAF1 and AFF4 [3, 36, 60], as well as POLR2A, the largest RNAPII subunit (Fig. 5a, Additional file 1: Fig. S6b). Indeed, RBM22 has been reported to be immunoprecipitated with RNAPII from human cells [7].

To confirm this interaction, we performed RNAPII immunoprecipitation in benzonase-treated cell lysates to eliminate any nucleic acid-dependent interactions. Efficient coprecipitation was observed when tagged RBM22 and the RNAPII subunit RPB3 were coexpressed by transient transfection of expression plasmids in HEK293T cells (Fig. 5b). As expected, the RNAPII-associated transcription elongation factor SPT5 was also coprecipitated by RBP3 (Fig. 5b). These results indicate that RBM22 associates with RNAPII in cells, consistent with the RBP3 protein interactome analysis [7].

To investigate the potential recognition of phosphorylated RNAPII, we used lentivirus to establish a cell line that stably expresses FLAG-tagged RBM22 and carried out coimmunoprecipitation to examine the binding of RBM22 to unphosphorylated, Ser5P and Ser2P RNAPII in this cell line. Strikingly, RBM22 preferentially interacts with Ser5P and Ser2P RNAPII in comparison with unphosphorylated RNAPII (Fig. 5c), consistent with the stronger co-occupancy of RBM22 and hyperphosphorylated RNAPII on chromatin (Fig. 1a, b). The same results were obtained from reciprocal coimmunoprecipitation experiments (Fig. 5d, e, f). Notably, the interaction between RBM22 and the P-TEFb component CDK9 as well as SPT5 was also detected (Fig. 5c).

To further elucidate whether the phosphorylation state of RNAPII CTD affects the recruitment of RBM22, we eliminated Ser5 and Ser2 phosphorylation by siRNA knockdown of CDK7, CDK9, and CDK12. As expected, CDK9 knockdown reduced Ser2P RNAPII, while CDK7 knockdown decreased both Ser5P and Ser2P RNAPII (Additional file 1: Fig. S6c), consistent with findings from previous studies using the CDK9 siRNA [61] and CDK7 inhibitor THZ1 [62–65]. Interestingly, CDK12 knockdown did not result in changes in RNAPII phosphorylation levels (Additional file 1: Fig. S6c), possibly due to the presence of its functionally redundant kinase, CDK13, in cells [66–68]. Clearly, the interaction between RBM22 and RNAPII was reduced by either CDK7 or CDK9 depletion but not CDK12 depletion (Fig. 5g, Additional file 1: Fig. S6c), showing that both Ser5 and Ser2 phosphorylation of RNAPII CTD are required for the recruitment of RBM22 to RNAPII. Notably, the downregulation of both Ser2P and Ser5P RNAPII upon CDK7 knockdown did not result in a stronger decrease in the interaction between RNAPII and RBM22 in comparison with CDK9 knockdown (Fig. 5g). It is possible that CDK9, as an interacting protein of RBM22 (Fig. 5a, c, and j) and RNAPII [31, 69], may also have a role in enhancing their interaction through protein–protein interaction. We further confirmed this requirement by treating the cells with the CDK9 inhibitor DRB and the pan-CDK inhibitor flavopiridol (Fig. 5h). Consistently, these inhibitors reduced the levels of Ser5 and Ser2 phosphorylated RNAPII (Additional file 1: Fig. S6d), meanwhile decreasing the association of RBM22 with RNAPII (Fig. 5h). To determine whether the association of RBM22 with RNAPII is transcription-dependent, we performed the co-IP experiments upon Actinomycin D treatment. We observed that Actinomycin D treatment leads to an increase in the levels of Ser2P RNAPII (Fig. 5i), consistent with previous observations [70, 71], however, the relative level of RBM22 associated with Ser2P RNAPII remains unchanged (Fig. 5i). These results suggest a transcription-independent interaction. Together with the genome-wide co-association between RBM22 and RNAPII, these results support the idea that RBM22 is recruited by 5′ paused and elongating RNAPII during transcription.

Interestingly, the RBM22 interactors also included the components of the P-TEFb-inhibiting 7SK snRNP [2, 72–74], such as LARP7, HEXIM1, and TRIM28 (Fig. 5a, Additional file 1: Fig. S6b), suggesting that RBM22 might also associate with the 7SK-P-TEFb complex. To test this idea, we first accessed the published RBM22 and LARP7 eCLIP data in HepG2 cells [35] and confirmed the interaction between RBM22 and 7SK noncoding RNA (Additional file 1: Fig. S6e), the core RNA component of the 7SK snRNP [74]. Furthermore, reciprocal IP experiments confirmed the interaction between RBM22 and the core protein components of the 7SK snRNP, such as LARP7, HEXIM1, and CDK9 (Fig. 5j, k).

The association of RBM22 with RNAPII, the spliceosome, and the 7SK-P-TEFb complex was further explored through glycerol gradient fractionation analysis of RBM22 complexes in cell lysates. Our results indicated that approximately 40% of RBM22 is linked with RNAPII, forming a large protein complex with the spliceosome (Fig. 5l, fractions 11–19). The remaining fraction of RBM22 forms a smaller complex with the free 7SK-P-TEFb complex under our sedimentation conditions (Fig. 5l, fractions 1–5). These findings, consistent with the co-IP data presented earlier, suggest that RBM22 may play a role in coordinating different RNAPII activities and splicing through its association with distinct complexes.

### RBM22-regulated homeostasis of the 7SK-P-TEFb complex on chromatin contributes to RNAPII 5′ pause and release

**Fig. 6:**
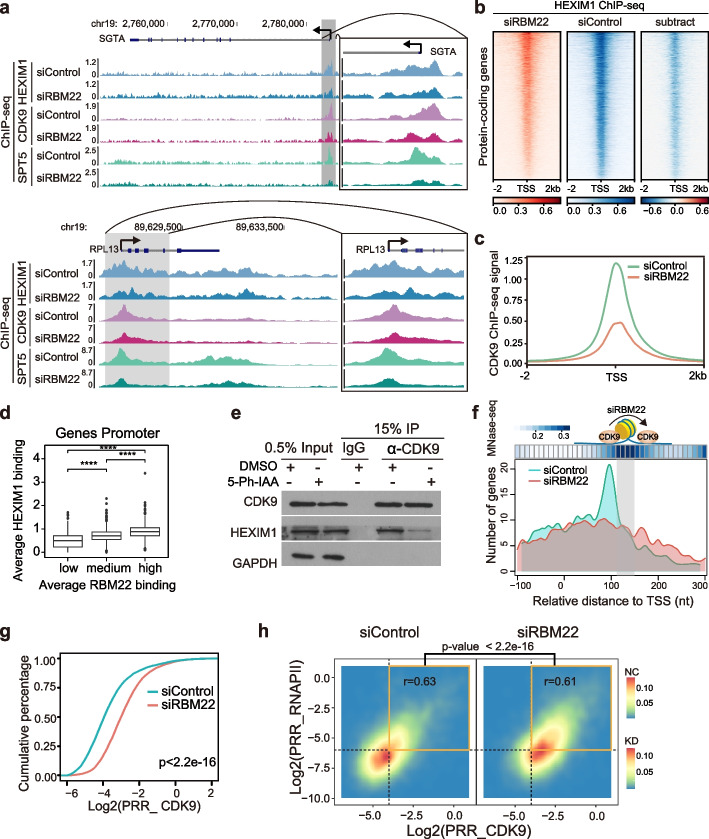
P-TEFb dynamics on chromatin coordinated regulated by RBM22 and inhibitory 7SK snRNP. **a** Examples of HEXIM1, CDK9, and SPT5 ChIP-seq signals at two representative protein-coding genes (*SGTA* and *RPL13*) in control or RBM22 knockdown HepG2 cells. **b** Heatmaps displaying the reduction in HEXIM1 ChIP-seq signal at protein-coding gene promoters upon RBM22 knockdown. For the subtraction of heatmaps, the color bars depict the subtracted values of siRBM22 minus siControl. **c** Metagene analysis showing the change in CDK9 ChIP-seq signal in the promoter-proximal regions of protein-coding genes in control or RBM22 knockdown HepG2 cells. **d** Boxplot analysis of HEXIM1 occupancy signal at gene promoters with different RBM22 binding signals. The 1068 genes with HEXIM1 binding at the promoter were equally divided into three groups based on RBM22 occupancy at gene promoters. **e** Co-IP assay examining the interaction between endogenous HEXIM1 and CDK9 in mAID-RBM22 cells with 5-Ph-IAA or DMSO treatment. **f** Distance distribution of CDK9 ChIP-seq peak summit relative to TSS and + 1 nucleosome dyads, determined by MNase-seq, showing the pause release of P-TEFb at chromatin level upon RBM22 knockdown. The *y*-axis represents the gene number of CDK9 accumulation at relative positions from summit to TSS in control and RBM22 knockdown cells. **g** CDK9 PRR distribution showing the stronger pause release of P-TEFb at protein-coding genes upon RBM22 knockdown. The *p* value was determined using the Kolmogorov–Smirnov test. **h** 2D density plot displaying the high correlation between CDK9 PRR and RNAPII PRR in control and RBM22 knockdown HepG2 cells. The yellow line box represents the gene with PRR values higher than the median, which were used to compare the PRR change for CDK9 and RNAPII before and after the RBM22 knockdown. The p-value was determined using the Fisher test

The functional impact of RBM22 on RNAPII pause release and its association with the inhibitory 7SK-P-TEFb complex suggests that RBM22 may affect the homeostasis of the 7SK-P-TEFb complex on chromatin. We therefore performed ChIP-seq of both inhibitory (HEXIM1) and activating (CDK9) components of the 7SK-P-TEFb complex (Additional file 1: Fig. S7a, b). Western blotting showed that RBM22 depletion did not affect the global protein levels of HEXIM1, LARP7, or CDK9 (Additional file 1: Fig. S7c). We obtained clear HEXIM1 and CDK9 ChIP-seq signals at promoter-proximal regions in control cells (Fig. 6a–c), similar to the previously reported results for ChIP-seq in HCT116, MCF7, and MEF cells [12, 73, 75], suggesting genome-wide inhibition of P-TEFb within the 7SK-P-TEFb complex at gene promoters in the human genome. Moreover, we observed that the HEXIM1 binding signal at promoters is associated with an increase in the RBM22 occupancy (Fig. 6d). More importantly, knockdown of RBM22 caused a strong reduction in the levels of HEXIM1 at gene promoters, as illustrated by the *SGTA* and *RPL13* gene loci (Fig. 6a) and apparent from the heatmap analysis (Fig. 6b). These results indicate that HEXIM1 is substantially disassociated from the gene promoter in the absence of RBM22, suggesting that RBM22 stabilizes the inhibitory 7SK-P-TEFb complex at promoters.

The disassociation of HEXIM1 from the gene promoter may result in the liberation of active P-TEFb from the inhibitory 7SK-P-TEFb complex, allowing it to process into the gene body (GB) from the promoter. To test this hypothesis, we initially assessed the release of P-TEFb from the 7SK-P-TEFb complex by examining the interaction between CDK9 and HEXIM1 through co-IP [76, 77]. Our findings indeed confirm a reduction in CDK9-bound HEXIM1 in the absence of RBM22 (Fig. 6e). Moreover, the depletion of RBM22 largely reduces the accumulation of CDK9 at the TSS but relatively increases CDK9 occupancy in the gene body on both example genes and genome-wide (Fig. 6a, c), displaying P-TEFb promoter-GB translocation (PGT) at the chromatin level in response to RBM22 depletion. We next determined the sites of CDK9 accumulation and the + 1 nucleosome in the promoter-proximal regions. The accumulation sites of CDK9 were defined as their binding summits detected by ChIP-seq (Method details), and ENCODE MNase-seq data in K562 cells were used to determine the position of + 1 nucleosome dyads [78]. Using metagene analysis, we found that CDK9 accumulates immediately upstream of the + 1 nucleosome at most gene promoters in control cells; however, such accumulation of CDK9 upstream of the + 1 nucleosome was apparently decreased, while CDK9 accumulation downstream of the + 1 nucleosome was clearly increased (Fig. 6f), suggesting that CDK9 was translocated from upstream to downstream of the + 1 nucleosome in the absence of RBM22 and supporting the idea that RBM22 inhibits P-TEFb PGT at promoters. In contrast, HEXIM1 showed a decrease in accumulation before the + 1 nucleosome, with only a subtle increase after the + 1 nucleosome (Additional file 1: Fig. S7d), consistent with its disassociation from the promoter (Fig. 6b). To generally evaluate P-TEFb PGT, we used CDK9 PRR to quantify the degree of P-TEFb PGT. As expected, an evident increase in the PRR of CDK9 was observed upon RBM22 depletion (Fig. 6g). Accordingly, CDK9 inhibition had the opposite effect on CDK9 PRR [79] (Additional file 1: Fig. S7e). Together with the disassociation of HEXIM1 from the promoter, these data support the idea that RBM22 represses P-TEFb PGT by stabilizing the 7SK-P-TEFb complex at promoters.

To determine whether P-TEFb PGT results in RNAPII pause release, we first analyzed the correlation between the RNAPII PRR and CDK9 PRR. Strikingly, a strong positive correlation between RNAPII PRR and CDK9 PRR was observed in control cells (Fig. 6h, left panel), in agreement with the correlation analysis of previous ChIP-seq data [79] (Additional file 1: Fig. S7f, left panel), suggesting that P-TEFb PGT appears to promote RNAPII pause release. Next, we examined the RNAPII PRR and CDK9 PRR in response to RBM22 depletion. Clearly, RNAPII PRR increases along with the elevated CDK9 PRR in response to RBM22 depletion (Fig. 6h, right panel), consistent with the coincident decrease in RNAPII PRR and CDK9 PRR under treatment with a specific CDK9 inhibitor [79] (Additional file 1: Fig. S7f, right panel), suggesting that upon RBM22 depletion, the P-TEFb PGT contributes to the RNAPII pause release. Intriguingly, we observed similar changes at snoRNA and snRNA genes, even though they are only several hundred base pairs long. However, these changes are consistent with the occurrence of readthrough transcription (Additional file 1: Fig. S7g, h and i). Taken together, these results suggest altered homeostasis of the 7SK-P-TEFb complex in the genome in the absence of RBM22 as a likely cause for RNAPII pause release at protein-coding genes as well as elevated transcription at snoRNA and snRNA genes.

### Loss of RBM22 disrupts the association of the elongation factor SPT5 with RNAPII

**Fig. 7:**
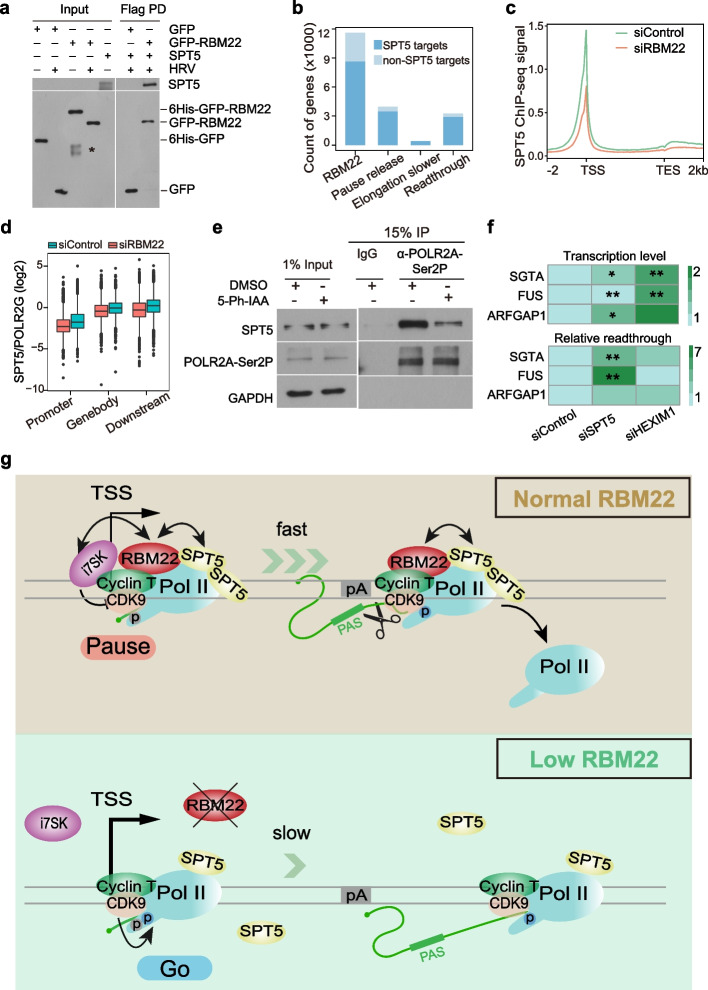
Loss of RBM22 impairs the association of the elongation factor SPT5 with RNAPII. **a** Recombinant proteins from pull-down assays visualized by immunoblotting. Input (purified 6HIS-GFP, 6HIS-GFP-RBM22 before and after the cleavage of HRV 3C protease and SPT5) and eluted proteins from immunoprecipitations (GFP or GFP-RBM22 incubated with SPT5) were visualized with the GFP and SPT5 antibodies (* indicates non-specific signal). **b** Barplot showing the number of SPT5 target genes within each category of RBM22 ChIP-seq binding, pause release, elongation slower, and readthrough genes. **c** Metagene analysis showing the change in SPT5 ChIP-seq signal at all protein-coding genes in control or RBM22 knockdown HepG2 cells. **d** Metagene profiles of RNAPII-normalized SPT5 occupancy levels at the promoter, gene body, and termination zone in control and RBM22 knockdown HepG2 cells. The 9076 genes with RNAPII binding were used. **e** Co-IP assay examining the interaction between endogenous SPT5 and Ser2P RNAPII (POLR2A-Ser2P) in mAID-RBM22 cells with DMSO or 5-Ph-IAA treatment. **f** TT-qPCR quantification of transcriptional level and transcriptional readthrough at three representative protein-coding genes in control, SPT5 knockdown (siSPT5) or HEXIM1 knockdown (siHEXIM1) HepG2 cells. Graphs show the ratios of relative readthrough, normalized to control. The *p* values are based on a two-tailed unpaired *t* test; *P < 0.05, **P < 0.01. **g** During early elongation by RNAPII at many protein-coding genes, RBM22 may initially be recruited by promoter-paused RNAPII, whose CTD is modified by Ser5 phosphorylation. This recruitment may stabilize promoter-associated inhibitory 7SK-P-TEFb complex to globally restrict P-TEFb PGT and subsequent RNAPII pause release in the sense and antisense direction. Moreover, RBM22 interacts with SPT5 and sustains the association between SPT5 and RNAPII, thus promoting RNAPII pausing at promoters. During productive elongation and termination at most genes, RBM22 may enhance the association of SPT5 to maintain the speed of elongating RNAPII and ensure efficient transcription termination. These roles of RBM22 facilitate the maintenance of RNAPII transcription homeostasis

Previous work showed that disruption of SPT5 can cause pause release [80], a reduced elongation rate [7], and transcriptional readthrough [81, 82]. We observed a clear interaction between RBM22 and SPT5 (Fig. 5c) in cells. To determine whether the interaction between RBM22 and SPT5 is direct, we incubated purified SPT5 with RBM22, both expressed and isolated from E. coli. Efficient co-precipitation of SPT5 was observed with GFP-RBM22 but not with GFP alone (Fig. 7a, Additional file 1: Fig. S8a, b), indicating a direct interaction. Furthermore, a strong co-occupancy of RBM22 and SPT5 in the genome (Fig. 7b) and genome-wide positive correlations between the binding of RBM22 and SPT5 at promoters, gene bodies, and downstream regions (Additional file 1: Fig. S8c, d) were observed in wild-type HepG2 cells, in agreement with the interplay between these two proteins. These data imply that RBM22 may strengthen the interaction between SPT5 and chromatin as well as RNAPII.

Remarkably, the vast majority of genes with changes in pause release, elongation rate, and transcriptional readthrough in RBM22-depleted cells harbor SPT5 binding (Fig. 7b). A very elegant hypothesis is that SPT5 may also contribute to RBM22-mediated transcription regulation. We therefore tested whether RBM22 depletion perturbed the chromatin localization of SPT5. Indeed, we observed a strong decrease in SPT5 occupancy across the entire gene locus in response to RBM22 depletion while western blotting showed that RBM22 depletion did not affect the global protein levels of SPT5 (Figs. 6a and 7c, Additional file 1: Fig. S7c). Moreover, the reduction in SPT5 binding on chromatin upon RBM22 knockdown increases with the level of RBM22 occupancy on chromatin (Additional file 1: Fig. S8e). These findings strongly suggest a requirement of RBM22 for the interaction of SPT5 with chromatin. When we normalized SPT5 occupancy signals to the corresponding RNAPII levels, the relative occupancy of SPT5 was strongly reduced at the promoter, GB and termination zone upon loss of RBM22 (Fig. 7d). Furthermore, we observed a reduced interaction between SPT5 and actively transcribing RNAPII following the acute depletion of RBM22 (Fig. 7e). Taken together, these findings provide support for the notion that RBM22 plays a role in enhancing the stability of the association between SPT5 and RNAPII during transcription. Furthermore, the genes with changes in pause release, elongation rate, and termination significantly overlapped (Additional file 1: Fig. S8f), supporting SPT5-mediated functional coupling. These findings suggest that RBM22 can link RNAPII 5′ pausing, elongation rate, and termination through its functional interaction with SPT5.

Since the SPT5 association with RNAPII also decreases at sno/snRNA genes upon RBM22 depletion (Additional file 1: Fig. S8g), we investigated whether SPT5 depletion is sufficient to induce readthrough of these types of genes. We first analyzed rRNA minus transcriptome data upon SPT5 knockdown in HeLa cells [83] and confirmed the detectable readthrough signals downstream of several sno/snRNA genes, including our example gene *RNU2-63P* (Additional file 1: Fig. S8h, left panel). Moreover, RBM22 depletion-induced readthrough at *U3*, *RNU1-60P*, *RNU2-63P*, and *RNVU1-6*, as shown in Fig. 4, is repeatable upon dTAG-mediated acute degradation of SPT5 [84] (Additional file 1: Fig. S8h, right panel). These data indicated that in addition to protein-coding genes, SPT5 is also required for sno/snRNA gene termination, suggesting that the decreased SPT5 association with RNAPII might be a factor for impaired termination of sno/snRNA genes.

To confirm the regulatory roles of inhibitory HEXIM1 and elongation factor SPT5 in RBM22-regulated transcription, we used TT-qPCR to examine the nascent RNA level in response to HEXIM1 and SPT5 depletion. For protein-coding genes, we readily detected an increase in transcriptional level and relative readthrough upon depletion of either protein (Fig. 7f, Additional file 1: Fig. S8i), while similar results were obtained for sno/snRNA genes (Additional file 1: Fig. S8j). Taken together, our findings suggest that the observed RBM22-dependent alternations in transcription elongation kinetics and termination defects are at least partially mediated by coordinating the 7SK-P-TEFb complex homeostasis and SPT5 dynamics.

## Discussion

We present a systematic study of RBM22 in transcriptional control and gene expression. Our knowledge of the RBM22 protein has largely been restricted to its function as a nuclear-localized RBP that promotes RNA splicing as part of the human spliceosome and has an essential role in zebrafish development [20, 35, 59, 85–87]. Our data suggest that RBM22 has a splicing-independent role in coordinating transcriptional programs in multiple aspects. First, RBM22 inhibits the RNAPII 5′ pause release. Second, RBM22 sustains a high transcription elongation rate. Third, RBM22 participates in transcription termination by RNAPII at both protein-coding genes and sno/snRNA genes. Intriguingly, these two types of genes undergo termination through distinct mechanisms [2, 9]; however, robust termination in both cases relies on the presence of RBM22. These roles of RBM22 facilitate the maintenance of RNAPII transcription homeostasis (Fig. 7g), reminiscent of the effect of the elongation factors ELOF1 and RECQL5 and the mRNA cleavage factor WDR33 [88–90]. Intriguingly, RBM22 displays a negative effect on RNAPII pause release but a positive effect on the elongation rate and termination process. There is no indication that any other RBPs or splicing factors possess such a function. The observations that loss of RBM22 increases pause release and that RBM22 is required to maintain rapid elongation seem contradictory. Probably, enhanced pause release results in transcription-induced DNA supercoiling accumulation, which might impede RNAPII elongation and reduce its rate in turn [91, 92]. It is worth noting that a converse effect was observed on the negative elongation factor RECQL5, which promotes RNAPII pause release but decreases its elongation rate [89]. These observations may reflect a transcriptional balance between pause release and elongation rate. Our observation of increased uaRNA signals downstream of the TSS following RBM22 depletion may also suggest a potential involvement of RBM22 in influencing chromatin conformation near the TSS. While the exact mechanisms underlying this phenomenon require further investigation, it prompts us to consider the possibility that RBM22, in addition to its known functions in transcriptional regulation, may contribute to shaping the local chromatin landscape. In any case, RBM22 represents a new versatile RNAPII transcription factor.

Given the direct role of RBM22 in transcriptional control, we speculate that the high abundance of RBM22 expression and its association with distinct machineries and complexes in cells allow it to have a dual function. Similarly, the U1 snRNP also shows a splicing-independent role in preventing premature termination and polyadenylation in introns [15]. Additionally, splicing inhibition by targeting U2 snRNP strongly impairs RNAPII pause release and reduces RNAPII elongation velocity at the beginning of genes, thereby decreasing RNA synthesis genome-wide [16]. Furthermore, SRSF2 also has a role in transcriptional control but promotes RNAPII pause release by liberating P-TEFb [12]. Splicing factors, by virtue of their involvement in the intricate process of mRNA maturation, may have evolved multifaceted roles to coordinate and fine-tune gene expression. One speculation is that splicing factors, being intimately connected with nascent RNA or regulatory non-coding RNA, could influence various steps of transcription, from initiation to elongation and termination. Additionally, they may interact with components of the transcription machinery, forming dynamic regulatory networks. This 'moonlighting' phenomenon raises intriguing questions about the evolution and functional versatility of splicing factors. Further investigations into the specific molecular mechanisms and evolutionary pressures driving splicing factors to take on additional roles in transcription will undoubtedly contribute to a deeper understanding of cellular regulatory networks.

The transcriptional process typically commences with chromatin opening at the promoter facilitated by pioneer transcription factors. Subsequently, RNAPII undergoes pre-initiation, initiation, 5′ pausing, pause release, productive elongation, and mRNA cleavage-triggered termination [1]. Our data indicate that RBM22 plays a role in this process by associating with 5′-paused and elongating RNAPII and interacting with gene loci. At promoters, RBM22 binds to and stabilizes the inhibitory 7SK-P-TEFb, which in turn represses the release of active P-TEFb and its PGT on chromatin. This, in effect, restrains the release of RNAPII pause. Furthermore, RBM22 may serve as a stabilizing factor for the association of SPT5 with RNAPII, facilitating RNAPII pausing at promoters, maintaining a consistent elongation rate, and ensuring efficient termination (Fig. 7g). Notably, the mechanism of transcription elongation activated by P-TEFb PGT observed in this study differs from that by the chromatin recruitment of released P-TEFb for transcription activation suggested by many previous studies [93–95]. Our data, together with previous literature [79], suggest that PGT of P-TEFb at the chromatin level may be an important mechanism of regulating gene transcription [79] (Fig. 6).

In addition to RBM22, other RBPs, such as DDX21 and WDR43, also have a role in regulating RNAPII pausing [96–98]. In addition, AGO1 enhanced RNAPII transcription in *Arabidopsis thaliana* by coordinating small noncoding RNAs and the SNF complex [99]. In contrast, RBM22 activity displays opposing functions in regulating RNAPII pause release by stabilizing the promoter-associated 7SK-P-TEFb complex and suppressing the release of active P-TEFb by restricting its promoter–GB translocation.

This study identifies the splicing factor RBM22, which acts as a key regulator of RNAPII transcription to orchestrate 5′-elongation control, elongation velocity, and transcription termination. Given the roles of RBM22 in development and human health [85, 100–102], investigating how RBM22 in transcriptional control contributes to physiological function and disease phenotypes will be an interesting future direction.

## Conclusions

In summary, we unveil a previously unknown, splicing-independent role of RBM22, a canonical splicing factor, in regulating multiple phases of RNAPII transcription, including 5′ pausing, elongation, and termination, to sustain the homeostasis of RNAPII transcription. Additionally, we demonstrate that RBM22 executes this function through mechanisms involving the coordination of the inhibitory 7SK-P-TEFb complex and the elongation factor SPT5. These findings challenge the traditional view of RBM22 solely as a splicing factor, suggesting a broad function in orchestrating RNAPII transcription.

## Methods

### Cell culture

HepG2 (HB-8065), HEK293T (CRL-11268), and derived cell lines were cultured according to ENCODE cell culture protocol. Briefly, HepG2 cells were cultured in MEM medium (Gibco, 11,095,080), supplemented with 10% fetal bovine serum (NEWZERUM, FBS-S500), 1 mM non-essential amino acids (Gibco, 11,140,050), 1 mM sodium pyruvate (Gibco, 11,360,070), 100 U/mL penicillin and 100 μg/mL streptomycin (Gibco, 15,140,148). HEK293T cells were grown in DMEM medium (Gibco, 11,965,084), supplemented with 10% fetal bovine serum, 100 U/mL penicillin, and 100 μg/mL streptomycin. All cell lines are free from mycoplasma contamination.

### General cloning

Human RBM22 cDNA was gifted by Professor Jiahuai Han (Xiamen University, China) and inserted into pLVX-Flag-Puro between AgeI and BamHI sites, and pcDNA3.0-HA between EcoRI and BamHI sites. Human RPB3 (POLR2C) cDNA was obtained by RT-PCR with total cDNA of HepG2 cells and inserted into pCDH-Flag between Xbal and BamHI sites. SPT5 full-length was cloned by PCR amplification and inserted into pET-28a by homologous recombination with NovoRec plus One step PCR Cloning Kit (Novoprotein, NR005). GFP or GFP-RBM22 containing Flag-HRV region were also recombined into pET-28a.

All sgRNAs were designed to target the region surrounding the start codon of RBM22, within a range of ± 50 base pairs. The sgRNA oligos were annealed and subsequently inserted into the pX459 plasmid. For the mAID-GFP RBM22 donor plasmid, the left and right homology arms were cloned by PCR amplification from the genomic DNA of HepG2. The Blasticidin-P2A-FLAG mAID-EGFP-HRV cassette, along with the left and right homology arms, was integrated into the pUC19 plasmid by NovoRec plus One step PCR Cloning Kit (novoprotein, NR005-01B). All used primers are listed in Table S5.

### Cell line manipulation and treatment

To generate stable cell lines expressing inducible FLAG-tagged RBM22, HEK293T cells were co-transfected with pLVX-Flag-RBM22-Puro, psPAX2 (Addgene # 12,260) and PMD2.G (Addgene # 12,259) to produce lentivirus. Virus-containing supernatant was collected 48 h post transfection to infect HepG2 cells in the presence of 8 µg/ml of polybrene. Then the infected cells were selected by puromycin. The expression of FLAG-RBM22 was induced by the addition of doxycycline for 48 h and assessed by western blotting. HEK293T cell lines expressing FLAG-RPB3 were generated as described above by co-transfection of pCDH-FLAG-RPB3, psPAX2, and PMD2.G.

For the generation of the parental degron cell line, the modified pMK381 plasmid (AAVS1 CMV-mCherry-OsTIR1 F74G) and AAVS1-targeting sgRNA were transfected into HepG2 cell lines. After 2 days, the cells were transferred and cultivated for 1 week in the presence of 4 μg/ml puromycin in the culture medium. Clonal lines were isolated using FACS enrichment of high mCherry-expressing cells.

For the construction of the RBM22 degron cell line, OsTIR1 (F74G) stable-expressing cells were transfected with RBM22 N-terminal targeting sgRNA, CRISPR/Cas9, and mAID-EGFP RBM22 donor plasmid. After 2 days, colonies were selected in the presence of 100 μg/ml Blasticidin. Cells with high GFP expression were isolated by FACS, and individual cells were sorted into a 96-well plate to obtain single clones. Single colonies were selected and analyzed using genomic PCR to confirm the genome type. Subsequently, Western blotting was performed to assess the expression levels of RBM22, comparable to those in wild-type HepG2 cells. Furthermore, homozygous knock-in cells were analyzed to determine their capacity for auxin-inducible degradation of mAID-EGFP-tagged RBM22.

SiRNA transfection was performed with Lipofectamine RNAiMAX (Invitrogen, 13,778,500) following its reverse transfection protocol. SiRNAs used in this study were purchased from GenePharma and their sequences are listed in Table S5. Plasmid transfection was carried out with Lipofectamine 2000 (ThermoFisher Scientific, 11,668,019) according to the manufacturer’s instruction. Transfected cells were harvested 48 or 72 h post transfection for experimental assays. For DRB and flavopiridol treatment, cells were incubated with 100 µM DRB (5,6-dichlorobenzimidazone-1-b-D-ribofuranoside, Sigma) for 3 h or 5 µM flavopiridol (MCE) for 30 min. For actinomycin D (Act D) treatment, cells were incubated with 5 µg/ml Act D (Sigma) for 90 min.

### Mass spectrometry

One to 2 × 10^8^ HepG2 cells expressing inducible FLAG-RBM22 were induced for 48 h by Dox (100 ng/ml final concentration) or H_2_O (control). Cells were harvested by centrifugation at 500 g for 6 min at 4°C. Cell pellets were suspended in cell lysis buffer (50 mM Tris HCl, pH 7.4, 50 mM NaCl, 1 mM EDTA, 1% TritonX-100, and proteinase inhibitor cocktail) containing 200 units/ml Benzonase (Thermo, 88,701). After rotating for 30 min at 4°C, the cell lysates were cleared by spinning at 13,000 rpm for 30 min at 4°C. The supernatants were incubated with ANTI-FLAG M2 Affinity Gel (Sigma-Aldrich, A2220) overnight at 4°C with rotation. Beads were washed four times with IP wash buffer (20 mM Tris–HCl pH 7.5, 150 mM NaCl, 1.5 mM MgCl_2_, 3 mM EDTA, 10% (v/v) glycerol, 0.1% (v/v) NP-40) and once with TE buffer (10 mM Tris–HCl pH 8.0, 1 mM EDTA). The immunoprecipitations were eluted using HRV 3C protease by incubation for 2 h at 22°C and subjected to label-free quantitative mass spectrometry.

### Co-immunoprecipitation and western blotting

Cells were lysed in cell lysis buffer containing phosphatase and protease inhibitors and 200 units/ml Benzonase for 30 min at 4°C. Cell debris was removed by centrifugation at 12,000 rpm for 30 min at 4°C. The supernatants were incubated with protein A/G magnetic beads (Thermo, 88,803) coupled with 5 µg antibody against FLAG (GenScript, A00187), POLR2A-S2P (Abcam, ab5095), POLR2A-S5P (Abcam, ab5408) or 8WG16 (Abcam, ab817) overnight at 4°C with rotation. Beads were washed four times with IP wash buffer and once with TE buffer. Beads were eluted using 30–100 µl TE buffer containing 10 mM DTT by shaking for 30 min at 37°C. Protein samples were loaded into 10% SDS-PAGE and transferred to PVDF membranes. After blocking and antibody incubation, the western blot signals were detected by SuperSignal West Pico plus chemiluminescent Substrate (Thermo).

### Glycerol gradient analysis

Glycerol gradient analysis was performed following a previously established protocol with some adjustments [103]. Briefly, a 10–40% glycerol gradient was prepared in NP-40 lysis buffer (50 mM HEPES, pH 7.5, 0.2 mM EDTA, 100 mM NaCl, 1 mM DTT, 1% NP-40, 1 × protease inhibitor cocktail) and allowed to stand for 1 h at 4°C. Subsequently, HepG2 cell lysate (500 μl) was carefully layered onto a 4 ml-gradient and underwent ultracentrifugation in a Beckman MLS-50 rotor at 50,000 rpm for 11 h at 4°C. The resulting gradient fractions (200 μl each) were manually collected and then precipitated with 10% trichloroacetic acid.

### TT-qPCR

TT-qPCR was performed as previously described [10] with a few modifications. Briefly, nascent RNA was labeled with 4sU (1 mM; Sigma, T4509) for 10 min at 37°C in cells. Cell nuclei were isolated with CE buffer (Hepes 10 mM, KCl 60 mM, EDTA 1 mM, NP40 0.1%, DTT 1mM, sucrose, 0.34 M) and lysed with TRIzol. Total RNA was purified by phenol–chloroform extraction and ethanol precipitation, fragmented to ~ 500 bp using Covaris S220, and then treated with RQ1 DNase (Promega, M6101) for 15min at 37°C. After treatment with antarctic phosphatase (NEB, M0289S), biotinylation of 4sU-labeled RNA was performed using MTSEA (5mg/ml; Biotium, BT90066) at R.T. in the dark. Biotinylated samples were extracted by phenol–chloroform and precipitated by ethanol to remove free biotin-XX and biotinylated RNA was captured by MyOne C1 streptavidin-coupled beads (Invitrogen, 65,001). After washing, the biotinylated RNA was eluted by proteinase K treatment. Eluted RNA was precipitated and subjected to reverse transcription and quantitative PCR. The 2-ΔΔCt method calculates relative readthrough expression by comparing the Ct values of the readthrough region to the last intron, normalizing them to WT, and computing fold change as 2^−ΔΔCt^. The primers used for TT-qPCR are listed in Table S5.

### ChIP-seq and ChIP-qPCR

ChIP-seq was performed as previously described [13]. Briefly, approximately 1 × 10^7^ cells were crosslinked for 20 min at room temperature in 1% formaldehyde solution diluted in PBS and then glycine was added to stop the reaction. Crosslinked cells were harvested and lysed in cell lysis buffer and then nuclei were isolated and resuspended in nuclear lysis buffer. Genomic DNA was sheared by sonication for 10 cycles with 10 s pulses and 50 s pausing under 40% output power. The clear supernatant was subjected to immunoprecipitation with antibody-coupled magnetic beads overnight at 4℃. After washing, elution, and decrosslinking, RNase and proteinase K treatments were used to remove RNA and protein respectively. Purified DNA was subjected to library construction using the VAHTS™ Universal DNA Library Prep Kit (Vazyme, ND606) and ChIP-qPCR. To calculate the relative pause release ratio, the 2-ΔΔCt method compares the differences in Ct values (Ct_(Input)_-Ct_(ChIP)_) between the gene body and promoter regions and then normalizes these values to the control sample. All the ChIP-qPCR primers used in this study are listed in table S5. ChIP-seq libraries between 200 and 500 bp were purified and sequenced by the Illumina Novaseq 6000. Antibodies used in this study are listed in Table S6.

### c3′-seq

Total RNA was isolated using TRIzol followed by two consecutive phenol–chloroform extractions and then precipitated by ethanol. Purified RNA was fragmented to ~ 500 bp with Covaris S220, followed by DNase digestion. Sheared RNA and RT-circulation-primer (Table S5) were used for reverse transcription, which was performed using HiScript® III 1st Strand cDNA Synthesis Kit (Vazyme; R312). After the removal of remaining RT primers with Exonuclease I (NEB, M029S), the cDNA from 150 to 500 nt was purified in 10% TBE-Urea gel. The cDNA circularization was performed using CircLigase II (epicenter, CL9021K) for 1 h at 60°C. Circularized products were re-linearized with APE1 (NEB, M0282S) and then amplified with Phanta Max Super-Fidelity DNA Polymerase (Vazyme; P505). C3′-seq libraries between 225 and 400 bp were purified and sequenced by the Illumina Novaseq 6000.

### Pulldown assays of recombinant proteins

Pull-down assays were generated as described [7] with the following modifications. Briefly, pET-28a plasmids (6His-SUMO-3Flag-HRV-GFP and 6His-SUMO-3Flag-HRV-GFP-RBM22) were transformed into BL21 E.coli. Pre-culture was inoculated in LB medium until an OD600 of 0.6. Overexpression was induced by adding 1 mM IPTG while shaking for 18 h at 16°C. Bacteria were pelleted and resuspended by vortexing 10 ml of lysis buffer (PBS, protease inhibitors). After sonication and centrifugation, proteins were purified on a disposable gravity flow column with Ni–NTA agarose. Purified proteins were concentrated by using centrifugal filter units (Millipore) and then second purified with ANTI-FLAG M2 Affinity Gel (Sigma-Aldrich, A2220).

For in vitro pull-down, GFP or GFP-RBM22 coupled beads were washed with pulldown buffer (100 mM NaCl, 20 mM Na-HEPES pH 7.5, 4% glycerol, 3 mM MgCl_2_, 1 mM 1,4-Dithiothreitol, 300 ng/ml BSA) incubated with purified SPT5 at 4°C for 3 h. After pull-down, beads were washed with pull-down buffer and NETN buffer (20 mM Tris pH 8.0, 100 mM NaCl, 1 mM EDTA, 0.5% NP-40). Pull-down was eluted using HRV 3C protease by incubation for 2 h at 22°C and separated by SDS-PAGE.

### ChIP-Seq data analysis

ChIP-seq data were processed according to the ENCODE uniform transcription factor ChIP-seq pipeline (https://www.encodeproject.org/chip-seq/transcription_factor) and using hg19 as the reference human genome. GENCODE v19 gene annotation was used for downstream analysis. PCR duplicates were removed using Picard v 2.26.5. The resulting reads were normalized to total reads aligned (reads per million, rpm). BigWig files were visualized using the UCSC Genome Browser (http://genome.ucsc.edu). Peak calling of ChIP-seq was performed with MACS version 2.1.2 (model-based analysis) [104]. Deeptools (v3.5.0) was used to calculate the average read density across defined genomic intervals and plot heatmaps of chromatin occupancy.

### GRO-Seq data analysis

GRO-seq data of RBP knockdown (GSE120105), generated in our previous study [13], were downloaded and mapped to the human genome using the Bowtie2 [105] with the local model. Non-redundant reads were determined by Samtools [106] and used for downstream analysis. Bam files were normalized based on the number of total reads with Samtools and bigwig files were built with the python package RSeQC. DoGs were identified using ARTDeco [57].

### RNA-seq data analysis

Taking advantage of the published fractional RNA-seq data [107], we simply quantified gene expression using featureCounts v1.6.2 tools from the Rsubread R package. Differential expression analysis was performed using the DESeq2 R package. For each gene, we analyzed basic types of alternative splicing events by using rMATS v4.1.1.

### c3′-seq data analysis

C3′-seq reads were firstly trimmed using cutadapt (v3.4) [108] to remove the adaptor and dT sequence at 3′ end and aligned against the hg19 genome with STAR (2.6.0a) [109]. The uniquely mapped reads were filtered and merged as a peak if they were within 30 bp from each other. The misprimed sites that have ten or more consecutive As in the downstream of the peak were removed and the remaining peaks were potential polyA sites detected by c3′-seq. Global alternative polyadenylation changes were quantified by PolyA-miner [110] from c3′-seq data.

### PRR calculation

Pause release ratios (PRR) were calculated by taking the read density in the gene body (500 bp downstream of the TSS to 500 bp upstream of the TES) divided by the read density of the promoter region (300 bp upstream of the TSS to 100 bp downstream of the TSS). FeatureCounts [111] (v1.6.2) was used to count reads within promoter and gene body intervals. Genes, which are occupied by RNAPII at promoters, longer than 2 kb, and located > 1 kb from the neighboring genes, were chosen for accurate calculation. If a gene has multiple TSSs, the TSS with the highest RNAPII occupancy, determined by POLR2G ChIP-seq, will be chosen for PRR analysis. PRR distribution plots were visualized using ggplot2 (v3.3.5).

### Readthrough analysis

GRO-seq data were used to measure transcriptional readthrough. Readthrough index (RI) was defined for two different groups of genes. For protein-coding genes, RI was the ratio of the GRO-seq read density in the downstream region of a gene relative to the GRO-seq read density in its last exon. If a gene has multiple annotated last exons, the last exon with the highest expression, determined by RNA-seq, will be chosen for analysis. For snoRNA and snRNA genes, a special RI was defined as the ratio of the GRO-seq read density in the region of twice the gene length located 100 bp downstream of TES to the GRO-seq read density in the gene body.

### Calculation of elongation rate

For DRB RNAPII ChIP-seq experiments, cells were treated with 100 μM DRB for 3 h. Then the cells were washed twice with PBS and incubated in DRB-free media for 0, 5, 10, or 20 min before crosslinking. For DRB POLR2A ChIP-seq metagene profiles, the signal was plotted for 50 bp bins for the region from the TSS to + 80 kb only for non-overlapping genes > 80 kb long. All the genes longer than 80 kb were used for elongation rate calculation (corresponding to 2217 human genes with non-overlapping transcription units). The RNAPII wave front was identified by first calculating the mean ChIP-seq signal for the region from + 60 to + 80 kb downstream of the TSS, which we defined as background. We then identified the position where the signal rose 5 standard deviations above the mean background signal and the position was defined as wave peak. Then elongation rates can be calculated from the position of the wave peak for each time point using linear regression. For a single gene, all the genes longer than 80 kb were divided into 50 bp bins (to a total of 80 kb) and the expressed signal of each 50-bp bin was quantified. Genes that are barely expressed or have missing values in more than 60% of the bins will be filtered out. Then we get a set of genes that pass an expression threshold to calculate the elongation rate. For each gene of each sample, the transcriptional wave front was identified in the same way as the meta-elongation rate calculation described above. Only the genes where the front edge of the wave was called at a position downstream of the wave for the previous time point were chosen. Finally, we fit a linear model to the wave peak positions of all samples (i.e., 0, 5, 10, 20 min post release) as a function of time to determine the rate of elongation.

### Sequencing data normalization

We used standard normalization techniques to remove effects originating from different sequencing depths, gene lengths, or both.

### Supplementary Information

**Additional file 1: Fig. S1**. The impact of RBM22 on gene expression and splicing. **Fig. S2.** The impact of RBM22 on RNAPII transcription at promoter-proximal regions. **Fig. S3.** Elongation rate analysis. **Fig. S4.** Functional feature of readthrough transcription. **Fig. S5.** RBM22-mediated transcriptional control at sno/snRNA genes and quality control of the c3'-seq. **Fig. S6.** Identification of interaction for RBM22. **Fig. S7.** Additional characterization of 7SK-P-TEFb and SPT5 dynamics regulated by RBM22. **Fig. S8.** RBM22 depletion leads to reduced SPT5 occupancy on chromatin. **Fig. S9.** Uncropped images for the blots in Fig. 1, 3, 5, 6, 7 and supplementary Fig. 1, 2, 3, 6, 7, 8.

**Additional file 2: Table S1.** Pause release ratio analysis of POLR2G ChIP-seq signals in HepG2 cells. **Table S2.** Readthrough index of protein-coding genes determined by GRO-seq in control and RBM22-depleted HepG2 cells. **Table S3.** Readthrough index of sno/snRNA and histone genes determined by GRO-seq in control and RBM22-depleted HepG2 cells. **Table S4.** Enriched partners of RBM22 interaction identified by label-free qMS. **Table S5.** Primers and siRNAs used in this study. **Table S6.** Antibodys used in this study.

**Additional file 3. **

## Data Availability

The accession numbers for the raw data FASTQ files and processed BigWig files for all sequencing data deposited in NCBI GEO are GEO: GSE220318. The published GRO-seq data for knockdown of different RBPs and RBM22 ChIP-seq data were downloaded from the GEO database under GSE120105 and GSE120104 [13]. The published MNase-seq data of K562 and RNAPII (POLR2A, POLR2A Ser5P, POLR2A Ser2P) ChIP-seq data of HepG2 were downloaded from ENCODE database under ENCSR000CXQ, ENCSR000EEM, ENCSR000EDX, ENCSR000BPI. The published CDK9 ChIP-seq data in THP-1 cells with DMSO or CDK9 inhibitor treatment were downloaded from GSE163804 [112]. The published TT-seq data in K562 cells treated with DMSO or Pla-B for 1 h was downloaded from GSE148433 [39]. The eCLIP data of RBM22 and LARP7 were downloaded from the ENCODE database under ENCSR961OKA and ENCSR456JJQ. The published RNA-seq data in Hela cells upon SPT5 knockdown were downloaded from GSE70268 [83]. The published TT-seq in SPT5-dTAG cells was downloaded from GSE180845 [84]. **Code availability** The source codes for the analysis including workflows in Snakemake and scripts in Python are available at the https://github.com/Duxian/RBM22_project [113] in Github. Raw scripts used in the paper can be found from https://doi.org/10.5281/zenodo [114]. All the other data generated in this study can be found in the manuscript and additional files.
