# Supplementary material for: RBM22 regulates RNA polymerase II 5′ pausing, elongation rate, and termination by coordinating 7SK-P-TEFb complex and SPT5
Source: Genome Biol. 2024 Apr 19;25:102. doi: 10.1186/s13059-024-03242-6 (PMC11027413; doi:10.1186/s13059-024-03242-6)
Supplement: Supplementary file 1 — Additional file 1: Fig. S1. The impact of RBM22 on gene expression and splicing. Fig. S2. The impact of RBM22 on RNAPII transcription at promoter-proximal regions. Fig. S3. Elongation rate analysis. Fig. S4. Functional feature of readthrough transcription. Fig. S5. RBM22-mediated transcriptional control at sno/snRNA genes and quality control of the c3'-seq. Fig. S6. Identification of interaction for RBM22. Fig. S7. Additional characterization of 7SK-P-TEFb and SPT5 dynamics regulated by RBM22. Fig. S8. RBM22 depletion leads to reduced SPT5 occupancy on chromatin. Fig. S9. Uncropped images for the blots in Fig. 1, 3, 5, 6, 7 and supplementary Fig. 1, 2, 3, 6, 7, 8. [file 13059_2024_3242_MOESM1_ESM.docx]

**Additional file 1: Supplementary figures**

**Title: RBM22 Regulates RNA Polymerase II 5' Pausing, Elongation Rate and Termination by Coordinating 7SK-P-TEFb complex and SPT5**

**Authors**: Xian Du^1,2,6^, Wenying Qin^1,2,6^, Chunyu Yang^1,2^, Lin Dai^1,2^, Mingkui San^1,2^, Yingdan Xia^1,2^, Siyu Zhou^1,2^, Mengyang Wang^1,2^, Shuang Wu^1,2^, Shaorui Zhang^1,2^, Huiting Zhou^1,2^, Fangshu Li^1,2^, Fang He^1,2^, Jingfeng Tang^3^, Jia-Yu Chen^4^, Yu Zhou^5^, Rui Xiao^1,2,7,*^

**Affiliations:**

^1^Department of Hematology, Medical Research Institute, Frontier Science Center for Immunology and Metabolism, Zhongnan Hospital of Wuhan University, Wuhan University, Wuhan, China

^2^TaiKang Center for Life and Medical Sciences, Wuhan University, Wuhan, China

^3^National "111" Center for Cellular Regulation and Molecular Pharmaceutics, School of Life and Health Sciences, Hubei University of Technology, Wuhan, China

^4^State Key Laboratory of Pharmaceutical Biotechnology, School of Life Sciences, Chemistry and Biomedicine Innovation Center, Nanjing University, Nanjing, China

^5^TaiKang Center for Life and Medical Sciences, College of Life Sciences, State Key Laboratory of Virology, Wuhan University, Wuhan, China.

^6^These authors contributed equally

^7^Lead contact

* To whom correspondence may be addressed. Email: xiaorui9@whu.edu.cn

**
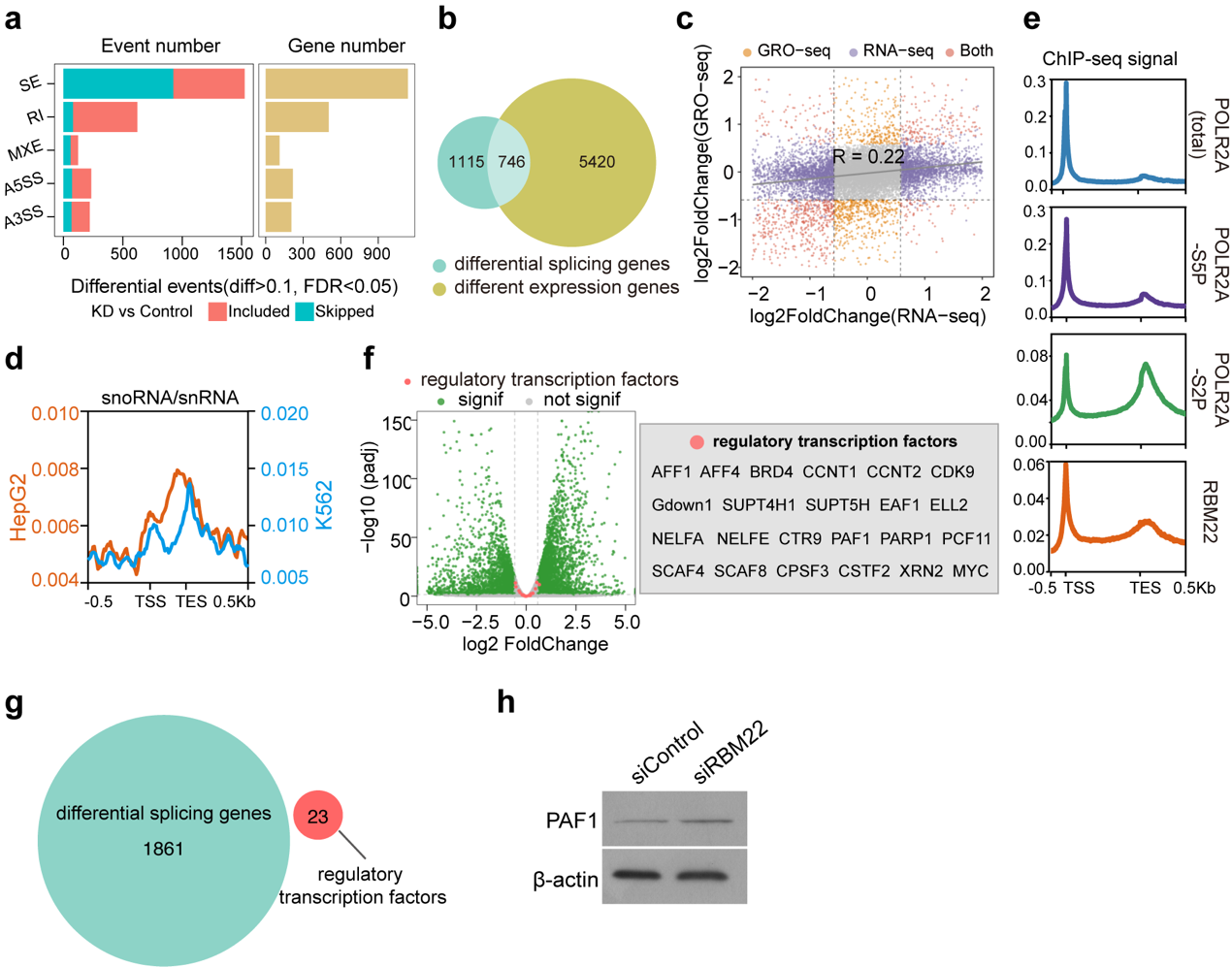
**

**Additional file 1: Fig. S1 The impact of RBM22 on gene expression and splicing.**

**(a)** Statistics of differential alternative splicing (AS) events and associated genes upon RBM22 knockdown. SE, skipped exon; MXE, mutually exclusive exon; A5SS, alternative 5’ splice site; A3SS, alternative 3’ splice site; RI, retained intron.

**(b)** Venn diagram showing the overlap between differentially expressed and alternative spliced genes detected by RNA-seq analysis in RBM22-depleted HepG2 cells.

**(c)** Comparison between the changes in gene expression profiled by RNA-seq and GRO-seq upon RBM22 knockdown in HepG2 cells. Pearson correlations are shown for genes expression change upon depletion of RBM22.

**(d)** Metagene analysis showing the occupancy of RBM22 at sno/snRNA genes in HepG2 and K562 cells.

**(e)** Metagene analysis showing the ChIP-seq signal of POLR2A (total), POLR2A-S5P, POLR2A-S2P and RBM22 at all protein-coding genes in K562 cells.

**(f)** Volcano plot showing the changes in gene expression profiled by RNA-seq (left) and a list of certain documented regulatory transcription factors (right). Significantly regulated genes are determined by padj of < 0.05 and log2FoldChange of < -0.58 or > 0.58.

**(g)** Venn diagram showing the overlap of differential splicing genes and the certain regulatory transcription factors.

**(h)** Efficient knockdown of RBM22 without affecting the PAF1 protein.


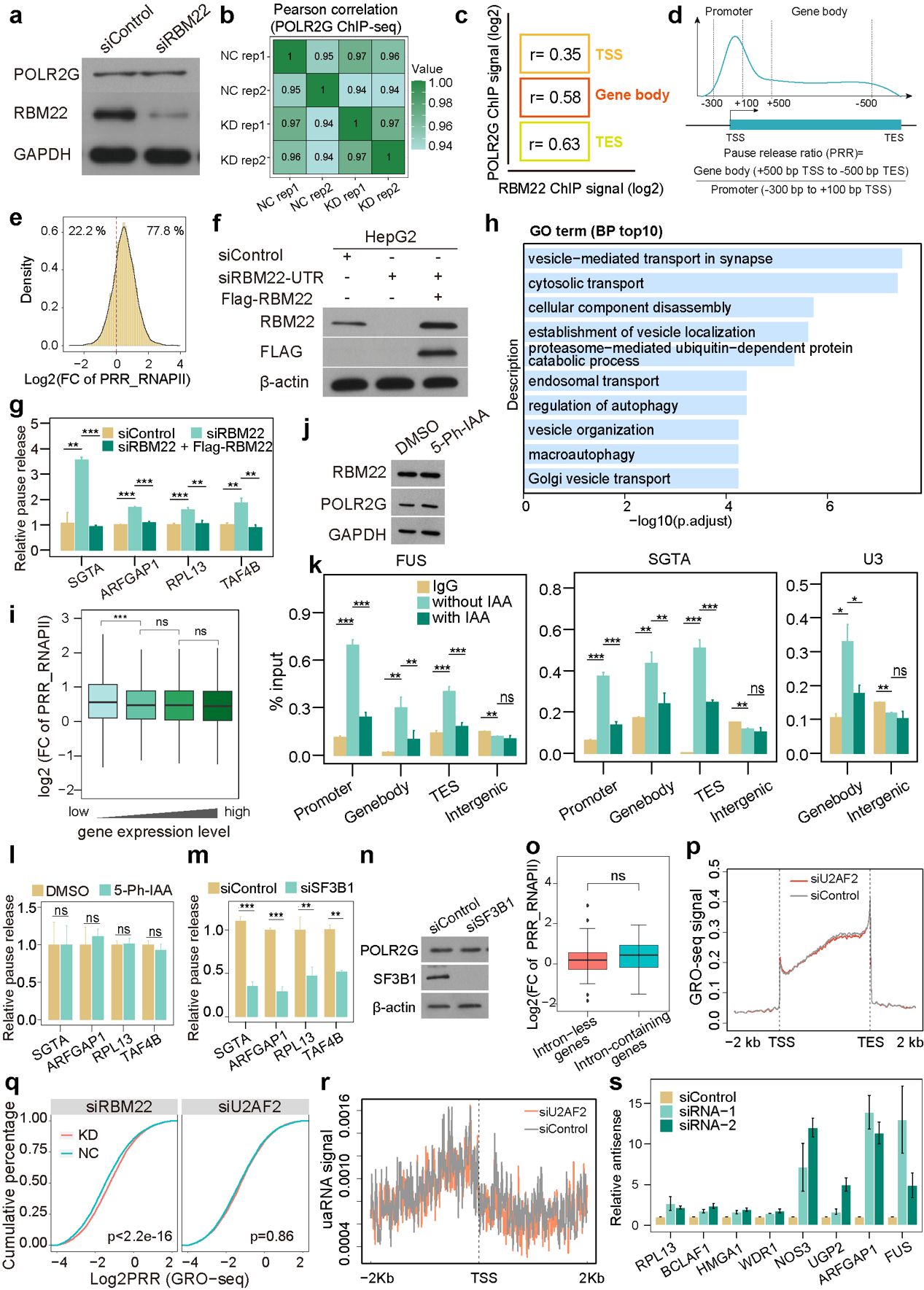


**Additional file 1: Fig. S2 The impact of RBM22 on RNAPII transcription at promoter-proximal regions.**

(**a**) Efficient knockdown of RBM22 without affecting the POLR2G protein.

**(b)** Pearson correlation analysis of POLR2G ChIP-seq data.

**(c)** The correlation between the POLR2G ChIP-seq signal and RBM22 ChIP-seq signal at promoter, gene body and TES.

**(d)** Schematic representation describing the calculation used to determine the pause release ratio (PRR) at each RNAPII-bound gene in HepG2 cells. The promoter region is defined using a fixed window from -300 bp to +100 bp around the transcription start site (TSS). The transcribed region (gene body) is from 500 bp downstream of the TSS to 500 bp upstream of the TES. The PRR is the ratio of RNAPII density in the transcribed region to that in the promoter region.

**(e)** Histogram showing the fold change (FC) of PRR (POLR2G ChIP-seq) at protein-coding genes upon RBM22 knockdown in HepG2 cells.

**(f)** Western blot analysis of RBM22 in HepG2 cells with depletion of RBM22 by siRNA (targeting the 3’-UTR), or restoration of physiological levels by re-expression of siRNA-resistant wild-type RBM22. β-actin serves as loading controls.

**(g)** POLR2G ChIP-qPCR quantification of RNAPII pause release at four representative protein-coding genes in control, RBM22 knockdown and re-expression of siRNA-resistant wild-type RBM22 after depletion of RBM22 HepG2 cells. Graphs show the ratios of relative pause release, normalized to control. The p values are based on a two-tailed unpaired t test; *P < 0.05, **P < 0.01.

**(h)** Barplot showing the gene ontology for the ten most significantly enriched biological processes for the genes strongly regulated by RBM22.

**(i)** Boxplot showing the fold change (FC) of PRR (POLR2G ChIP-seq) at genes with different degree of gene expression in response to RBM22 depletion. The 9065 genes were equally divided into four groups based on the gene expression levels.

**(j)** Western blot showing the protein abundance of RBM22 and POLR2G in wild-type HepG2 cells with DMSO or 5-Ph-IAA treatment.

**(k)** ChIP-qPCR analysis of the RBM22 binding level at the promoter, genebody, TES and intergenic of the protein-coding genes or at the genebody and intergenic of the snoRNA gene. The p values are based on a two-tailed unpaired t test; *P < 0.05, **P < 0.01, ***P < 0.001, ****P < 0.0001.

**(l)** POLR2G ChIP-qPCR quantification of RNAPII pause release at four representative protein-coding genes in wild-type HepG2 cells with DMSO or 5-Ph-IAA treatment. Graphs show the ratios of relative pause release. The p values are based on a two-tailed unpaired t test; *P < 0.05, **P < 0.01, ***P < 0.001.

**(m)** POLR2G ChIP-qPCR quantification of RNAPII pause release at four representative protein-coding genes in control and SF3B1 knockdown HepG2 cells. Graphs show the ratios of relative pause release. The p values are based on a two-tailed unpaired t test; *P < 0.05, **P < 0.01, ***P < 0.001.

**(n)**Western blot showing the protein abundance of SF3B1 and POLR2G in wild-type cells or in SF3B1 depletion HepG2 cells.

**(o)** Boxplot showing the fold change (FC) of PRR (POLR2G ChIP-seq) for intron-less genes and genes bearing introns with the same gene length distribution. 70 intron-less genes and 70 intron-containing genes, both within the length range of 2 kb to 7 kb, were selected to ensure a similar length distribution.

**(p)** Metagene analysis showing the elevated GRO-seq signals at many protein-coding genes in U2AF2 knockdown HepG2 cells (siU2AF2).

**(q)** PRR calculation of GRO-seq signals in control, RBM22 knockdown or U2AF2 knockdown HepG2 cells, showing the increased RNAPII pause release upon RBM22 knockdown. ***p < 0.001, ns=no significant (Kolmogorov–Smirnov test).

**(r)** Metagene analysis of antisense transcription, detected by GRO-seq, at TSS in control and U2AF2 knockdown HepG2 cells.

**(s)** TT-qPCR quantification of antisense transcription in HepG2 cells treated with two independent RBM22-targeted siRNAs. Error bars represent the SD. The p values are determined using the two-tailed unpaired t-test in comparing different siRNA to the control (*p<=0.05; **p<=0.01; ns, not significant).

**
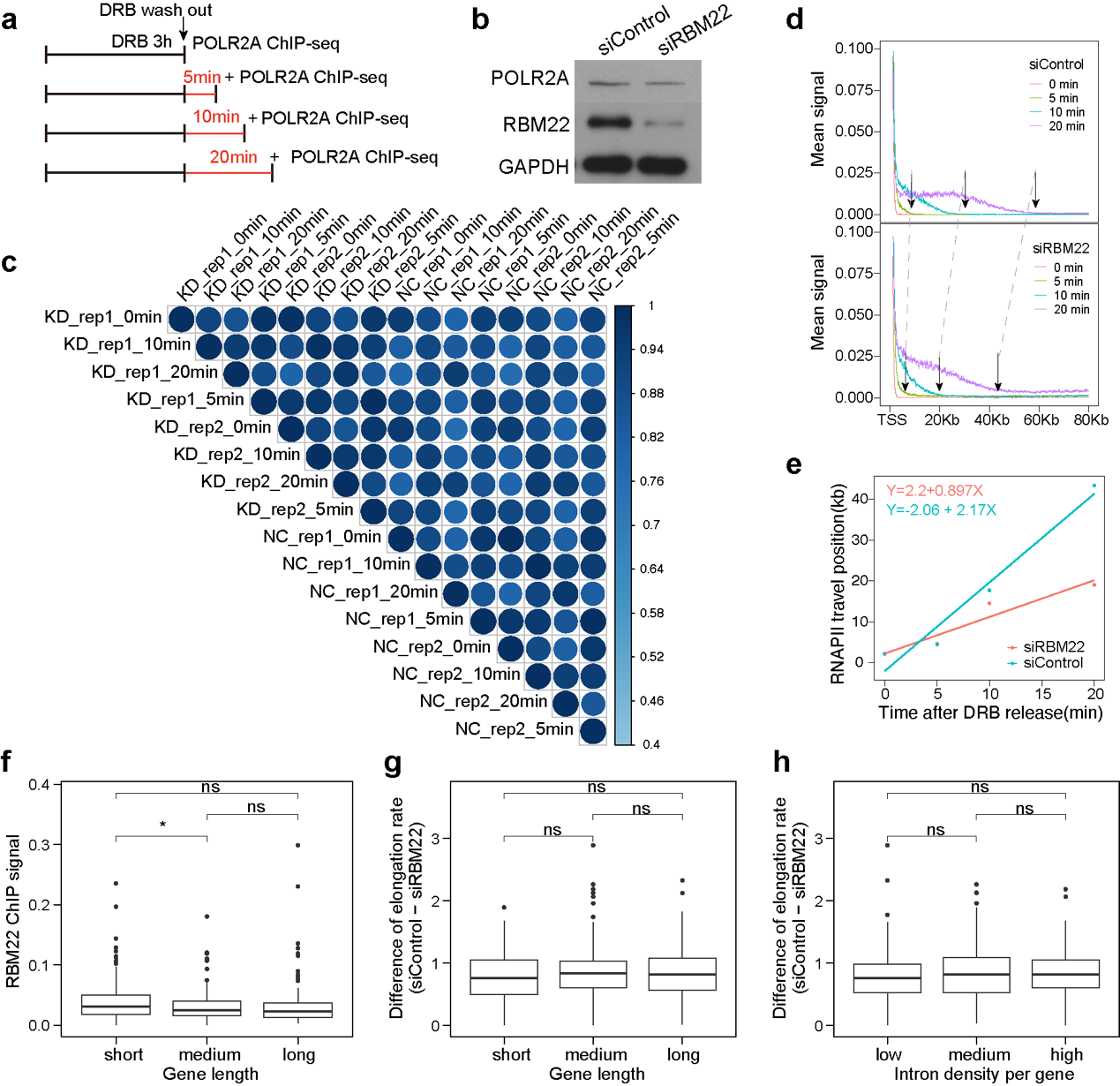
**

**Additional file 1: Fig. S3 Elongation rate analysis.**

**(a)** Schematic of DRB/POLR2A ChIP-seq to measure RNAPII elongation rates.

(**b**) Efficient knockdown of RBM22 without affecting the POLR2A protein.

**(c)** Pearson correlation analysis of DRB/POLR2A ChIP-seq data.

**(d)** DRB/POLR2A ChIP-seq metagene profiles at the genes longer than 80 Kb. Arrows indicate the front of the transcription wave for second replicate experiments.

**(e)** Calculation of the RNAPII average elongation rates based on metagene profiles using linear regression for second replicate experiments.

**(f-g)** Boxplot analysis of the RBM22 occupancy signals (**f**) and the elongation rate change upon RBM22 knockdown (**g**) for genes with different gene length. The genes were divided into three groups based on gene length: short (length < 112.2kb), medium (112.2kb < length < 170.2kb) and long (length > 170.2kb).

**(h)** Boxplot analysis of the elongation rate change upon RBM22 knockdown for genes with different intron density. The genes were divided into three groups based on intron density of each gene. The p values are determined using the two-tailed unpaired t-test (*p<=0.05; **p<=0.01; ***p<=0.001; ****p<0.00001)


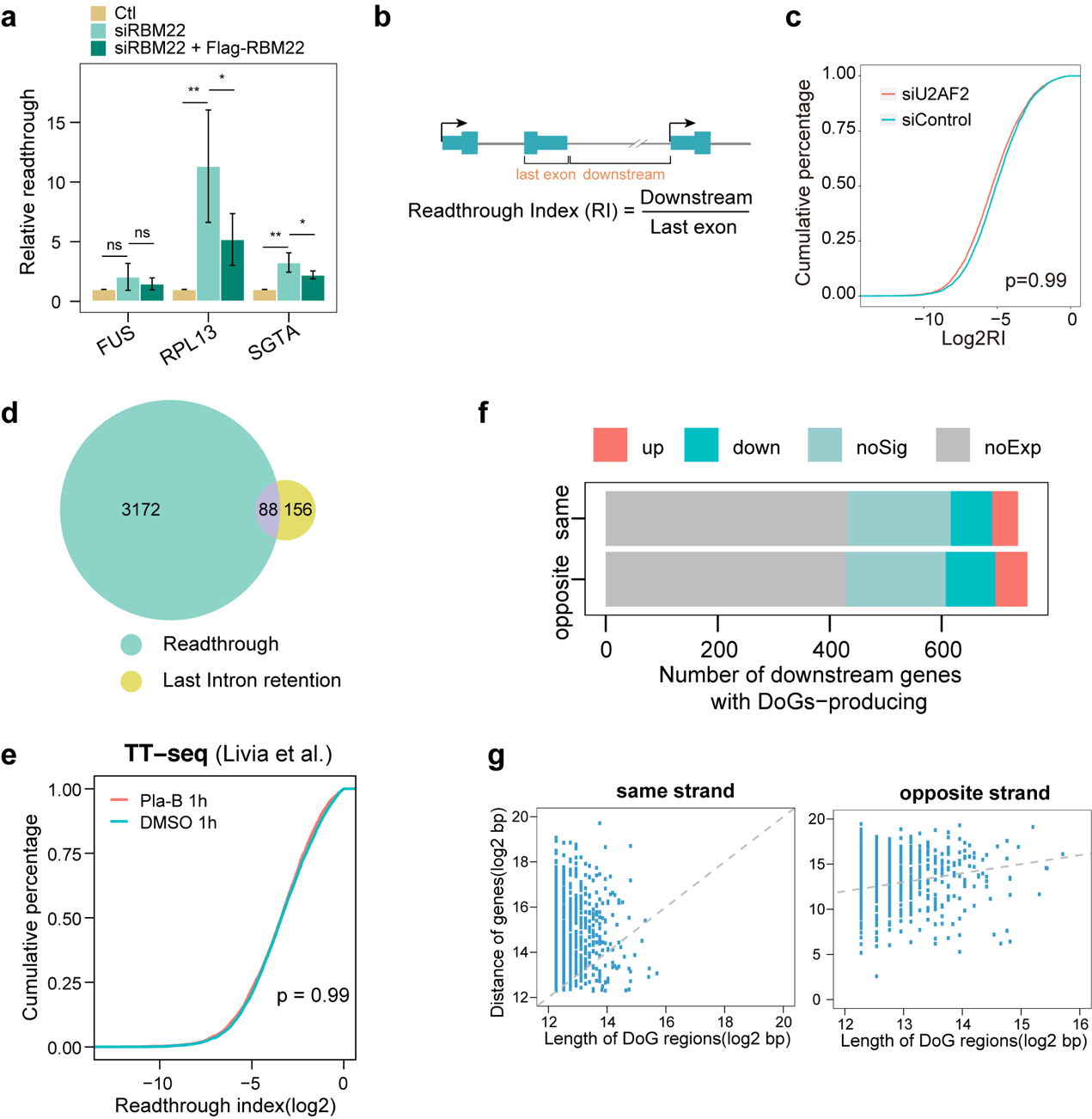


**Additional file 1: Fig. S4 Functional feature of readthrough transcription.**

**(a)** TT-qPCR quantification of transcriptional readthrough at three example protein-coding genes in control, RBM22 knockdown or re-expression of siRNA-resistant wild-type RBM22 after depletion of RBM22 HepG2 cells. Error bars represent the SD. The p values are determined using the two-tailed unpaired t-test (*p<=0.05; **p<=0.01; ns, not significant).

**(b)** Readthrough index (RI) defined as the ratio of GRO-seq read density in the region from TES to the TSS of downstream gene to the GRO-seq read density in the terminal exon.

**(c)** RI distribution in control and U2AF2 knockdown HepG2 cells, showing the no change upon U2AF2 knockdown. The p value was determined using the Kolmogorov–Smirnov test.

**(d)**Venn diagram showing the overlap between the genes with readthrough changes and last intron retention.

**(e)** RI distribution for protein-coding genes in K562 cells treated with DMSO or Pla-B for 1h. The p value was determined using the Kolmogorov–Smirnov test.

**(f)** Barplot showing the number of differentially expression genes for downstream genes of DoGs-producing genes in both directions after RBM22 knockdown. Unexpressed genes (noExp), genes with no significant change (noSig).

**(g)** Comparison between the length of DoGs region and the distance between the DoGs-producing genes and their nearest downstream genes.

**
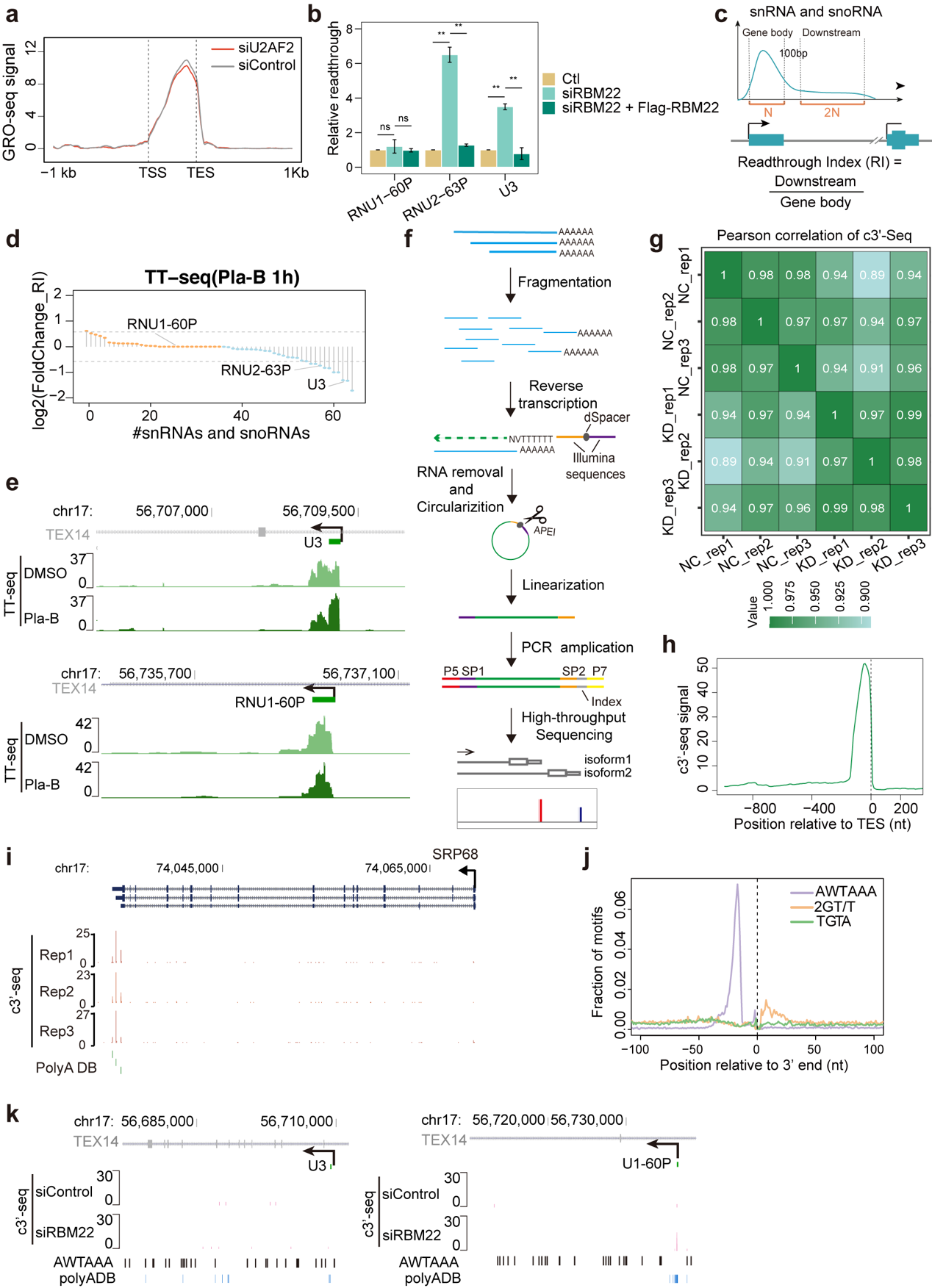
**

**Additional file 1: Fig. S5 RBM22-mediated transcriptional control at sno/snRNA genes and quality control of the c3’-seq.**

**(a)** Metagene analysis showing the no change in GRO-seq signals at independently transcribed snoRNA and snRNA genes upon U2AF2 knockdown.

**(b)** TT-qPCR quantification of transcriptional readthrough at three representative sno/snRNA genes (*U3, RNU1-60P* and *RNU2-63P*) in control, RBM22 knockdown and re-expression of siRNA-resistant wild-type RBM22 after depletion of RBM22 HepG2 cells. Graphs show the ratios of relative readthrough, normalized to control. Error bars represent the SD. The p values are determined using the two-tailed unpaired t-test (*p<=0.05; **p<=0.01; ns, not significant).

**(c)** Schematic representation describing the special calculation used to determine the readthrough index (RI) for sno/snRNA and intronless histone genes. The downstream region is defined as the region of twice the gene length located 100 bp downstream of the TES. The RI of sno/snRNA and intronless histone genes is the ratio of GRO-seq read density in the downstream region to the GRO-seq read density in the gene body.

**(d)** RI distribution for sno/snRNA genes in K562 cells treated with DMSO or Pla-B for 1h. The p value was determined using the Kolmogorov–Smirnov test.

**(e)** Examples of TT-seq signal at two representative sno/snRNA genes (*U3* and *RNU1-60P*) upon 1h DMSO or Pla-B.

**(f)** Schematic illustration of c3’-seq for detecting polyA sites of mRNAs.

**(g)** Pearson correlation analysis of c3’-seq data.

**(h)** Metagene analysis showing the enriched c3’-seq signals near the TES of annotated protein-coding genes in HepG2 cells.

**(i)** Example of c3’-seq signals at *SRP68* locus showing the robust detection of the polyA sites of the mRNA, referred by polyA database (polyADB).

**(j)** Positional distribution of polyA site related motifs (AWTAAA, 2GT/T, TGTA) within ±100 bp windows around 3′ ends of mRNAs.

**(k)** Example of c3’-seq signals at *U3* and *RNU1-60P* showing no polyA signals in their sRDoG regions.

**
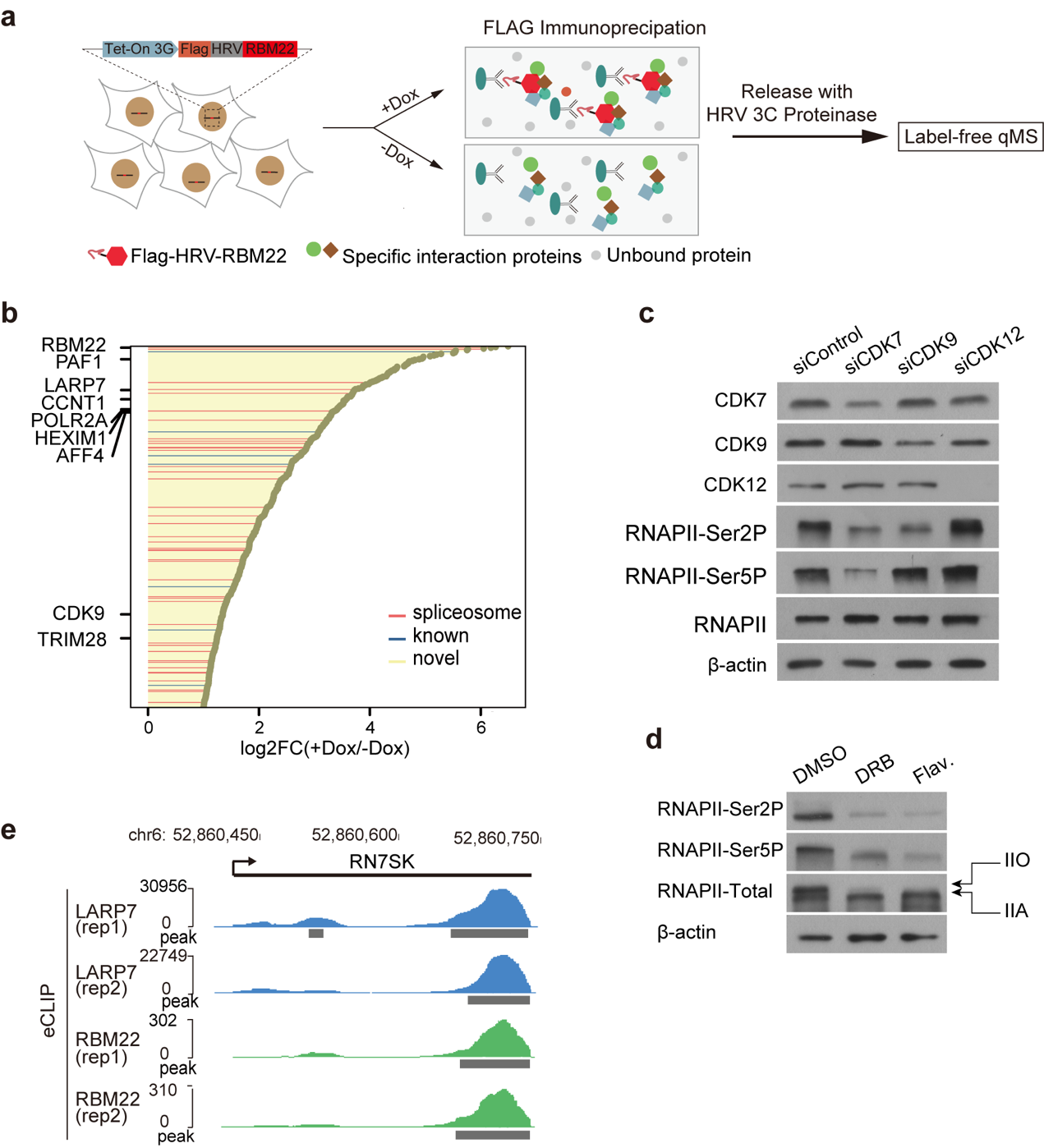
**

**Additional file 1: Fig. S6 Identification of interaction for RBM22.**

**(a)** Graphic displaying the method used to identify RBM22-associated proteins by FLAG-directed immunoprecipitation (IP) followed by label-free quantitative mass spectrometry (qMS).

**(b)** Histogram showing the enriched partners of RBM22 interaction identified by label-free qMS. Red line indicated the spliceosome proteins, blue line indicated the known interacting proteins with binding evidence in STRING database and light yellow lines indicated the novel interacting proteins. The x axis displays the enrichment (log2FC) of proteins in Flag-RBM22 expressing cells compared to control cells. +Dox/-Dox indicates LFQ indensity before and after induction of RBM22 expression.

**(c)** Western blot results showing the knockdown efficiency of the siRNAs against CDK7, CDK9, CDK12 and the effect of CDK7, CDK9, CDK12 knockdown on the levels of Pol II phosphorylation. β-actin serves as loading control.

**(d)** Western blot results showing the proteins level of RNAPII-Ser2P, RNAPII-Ser5P and RNAPII-Total in HepG2 cells with DMSO, DRB or flavopiridol treatment. Hypophosphorylated (IIa) and hyperphosphorylated (IIo) RNAPII are indicated.

**(e)** Genome browser track showing the directing binding of RBM22 and LARP7 on 7SK noncoding RNA, determined by eCLIP.


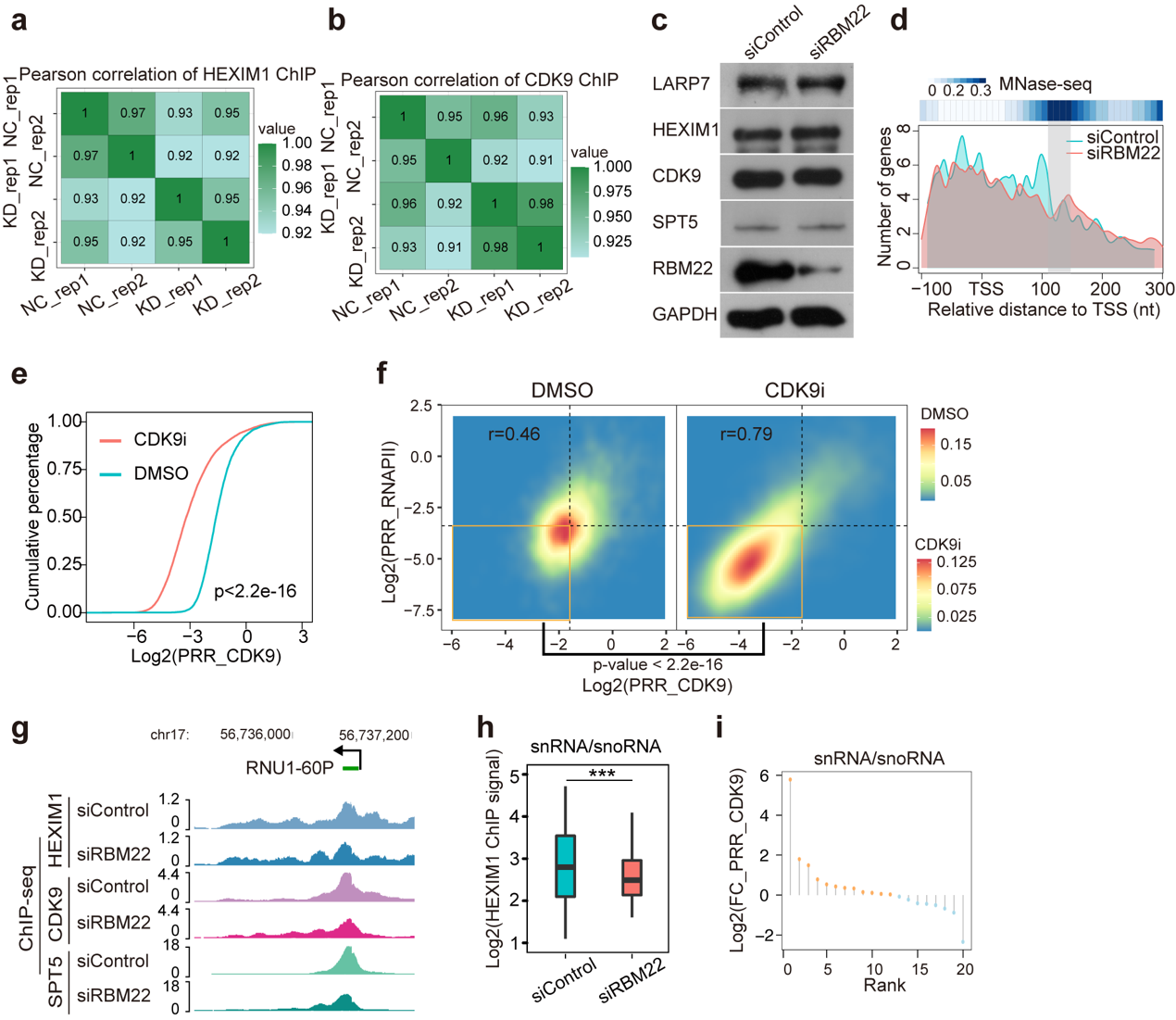


**Additional file 1: Fig. S7 Additional characterization of 7SK-P-TEFb and SPT5 dynamics regulated by RBM22.**

**(a-b)** Pearson correlation analysis of HEXIM1 (**a**) and CDK9 ChIP-seq (**b**).

**(c)** Western blot results showing the unchanged protein level of LARP7, HEXIM1 and CDK9 in RBM22 knockdown HepG2 cells.

**(d)** Distance distribution of HEXIM1 ChIP-seq peak summit relative to TSS and +1nucleosome dyads. The +1nucleosome position is determined by MNase-seq. The y axis represents the gene number of HEXIM1 accumulation at relative positions from summit to TSS in control and RBM22 knockdown cells.

**(e)** CDK9 PRR distribution showing that CDK9 inhibitor strongly suppressed P-TEFb pause release at protein-coding gene promoters in THP-1 cells. The p-value was determined using the Kolmogorov–Smirnov test.

**(f)** 2D density plot displaying the positvie correlation between CDK9 PRR and RNAPII PRR in control THP-1 cells and correlated changes in CDK9 PRR and RNAPII PRR in response to CDK9 inhibitor. The yellow line box represents the gene with PRR values higher than the median, which were used to compare the PRR change for CDK9 and RNAPII before and after RBM22 knockdown. The p-value was determined using the fisher test.

**(g)** Boxplot displaying the decreased HEXIM1 ChIP-seq signals at snRNA/snoRNA genes in response to RBM22 knockdown in HepG2 cells.

**(h)** An example of HEXIM1, CDK9 and SPT5 ChIP-seq signals at a representative snoRNA/snRNA gene (*RNU1-60P*) in control and RBM22 knockdown HepG2 cells.

**(i)** Histogram showing the CDK9 PRR fold changes of sno/snRNA genes upon RBM22 knockdown, ranked according to PRR value.


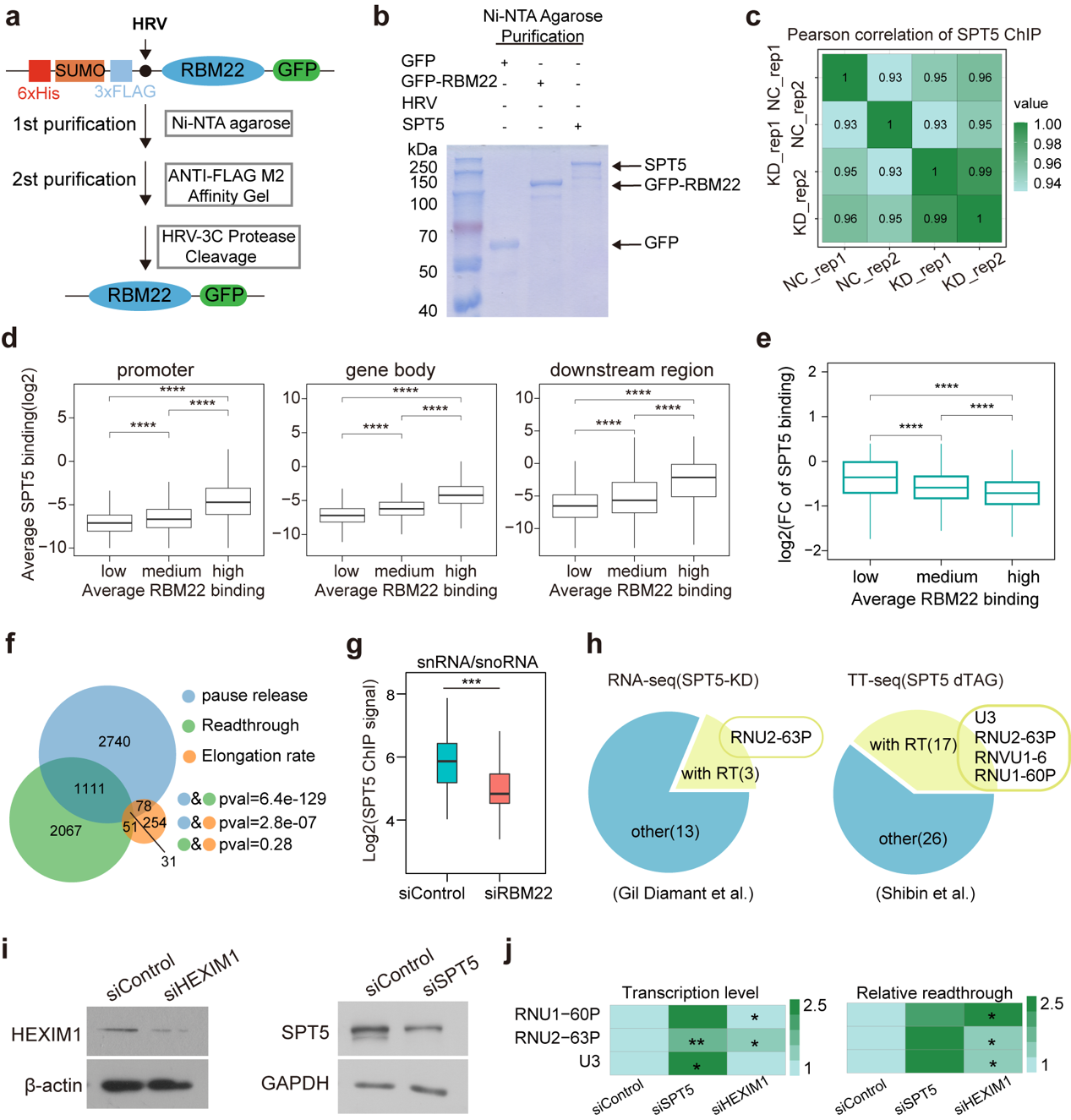


**Additional file 1: Fig. S8 RBM22 depletion leads to reduced SPT5 occupancy on chromatin.**

**(a)** Scheme of the construct and affinity purification procedure. The two-step affinity purification consists of gravity flow with Ni-NTA agarose and incubation with ANTI-FLAG M2 Affinity Gel. Pull-down was eluted using HRV 3C protease.

**(b)** Polyacrylamide gel electrophoresis (PAGE) of proteins from Ni-NTA agarose purification visualized by Coomassie Brilliant Blue.

**(c)** Pearson correlation analysis of SPT5 ChIP-seq data.

**(d)** Boxplot analysis of SPT5 occupancy signals at gene promoters, gene body or downstream regions with different RBM22 binding signals. The genes were divided into three groups, respectively, based on RBM22 occupancy at gene promoters, gene body or downstream regions.

**(e)** Boxplot analysis of the changes in SPT5 ChIP-seq signals at genes with different RBM22 occupancy upon RBM22 knockdown. The genes were divided into three groups based on RBM22 binding signals across genes in control cells.

**(f)** Venn diagram showing the overlap between the genes with the changes in pause release, readthrough and elongation rate.

**(g)** Boxplot displaying the decreased SPT5 ChIP-seq signals at snRNA/snoRNA genes upon RBM22 knockdown in HepG2 cells.

**(h)** Pie plot displaying the number of genes with increased readthrough upon SPT5 knockdown (RNA-seq) in HeLa cells (left panel) and SPT5 degradation (TT-seq) in DLD1 cells (right panel), respectively. The increased readthrough at *U3*, *RNU1-60P*, *RNU2-63P*, *RNVU1-6* was repeated in these datasets.

(**i**) Western blot results showing the efficiency of the siRNA against HEXIM1 or SPT5 in HepG2 cells.

**(j)** TT-qPCR quantification of transcriptional level and readthrough at three representative protein-coding genes in control, SPT5 knockdown and HEXIM1 knockdown HepG2 cells. Graphs show the ratios of relative readthrough, normalized to control. The p values are based on a two-tailed unpaired t test; *P < 0.05, **P < 0.01.

**Additional file 1: Fig. S9 Uncropped images for the blots in Fig. 1, 3, 5, 6, 7 and supplementary Fig. 1, 2, 3, 6, 7, 8.**

**Fig1.f**


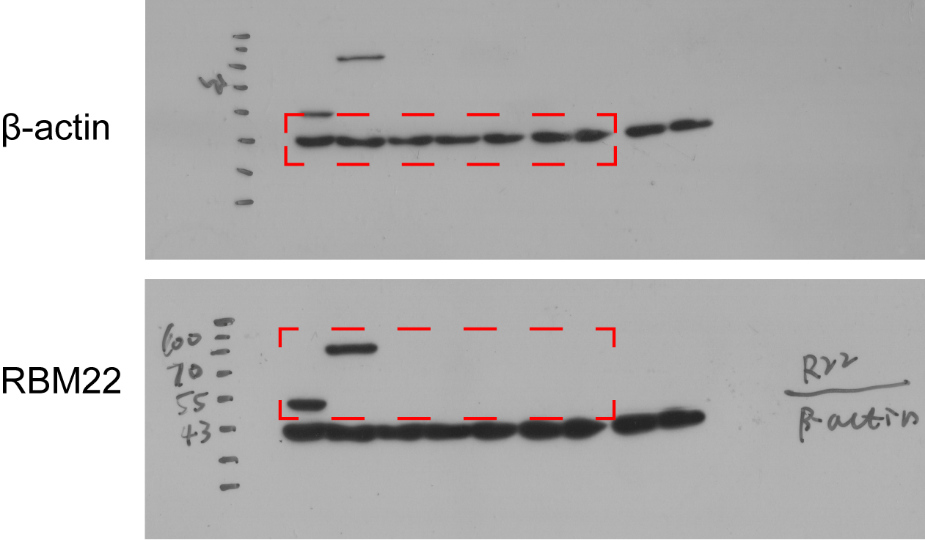


**Fig5.b**

**
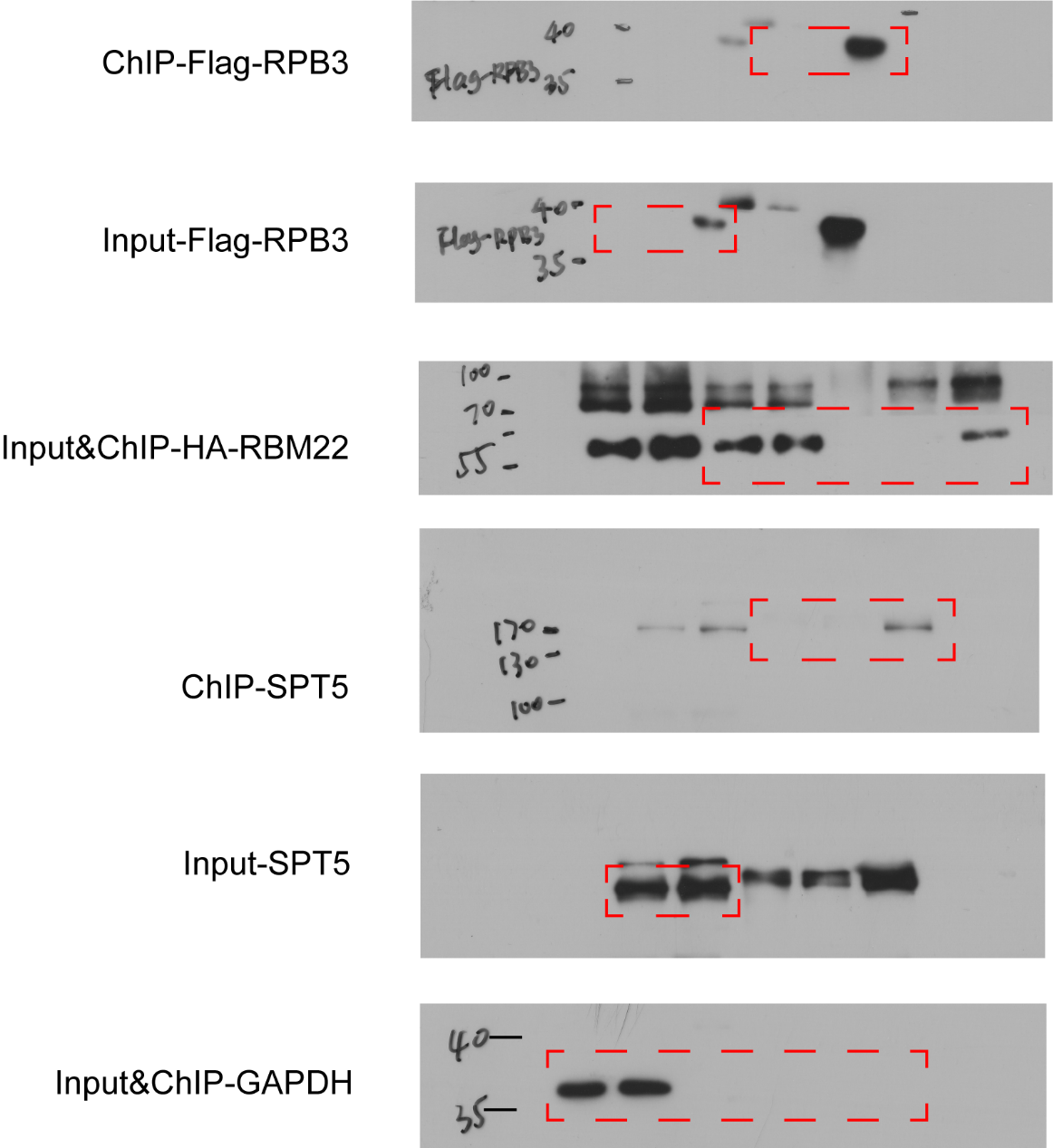
**

**Fig5.c**

**
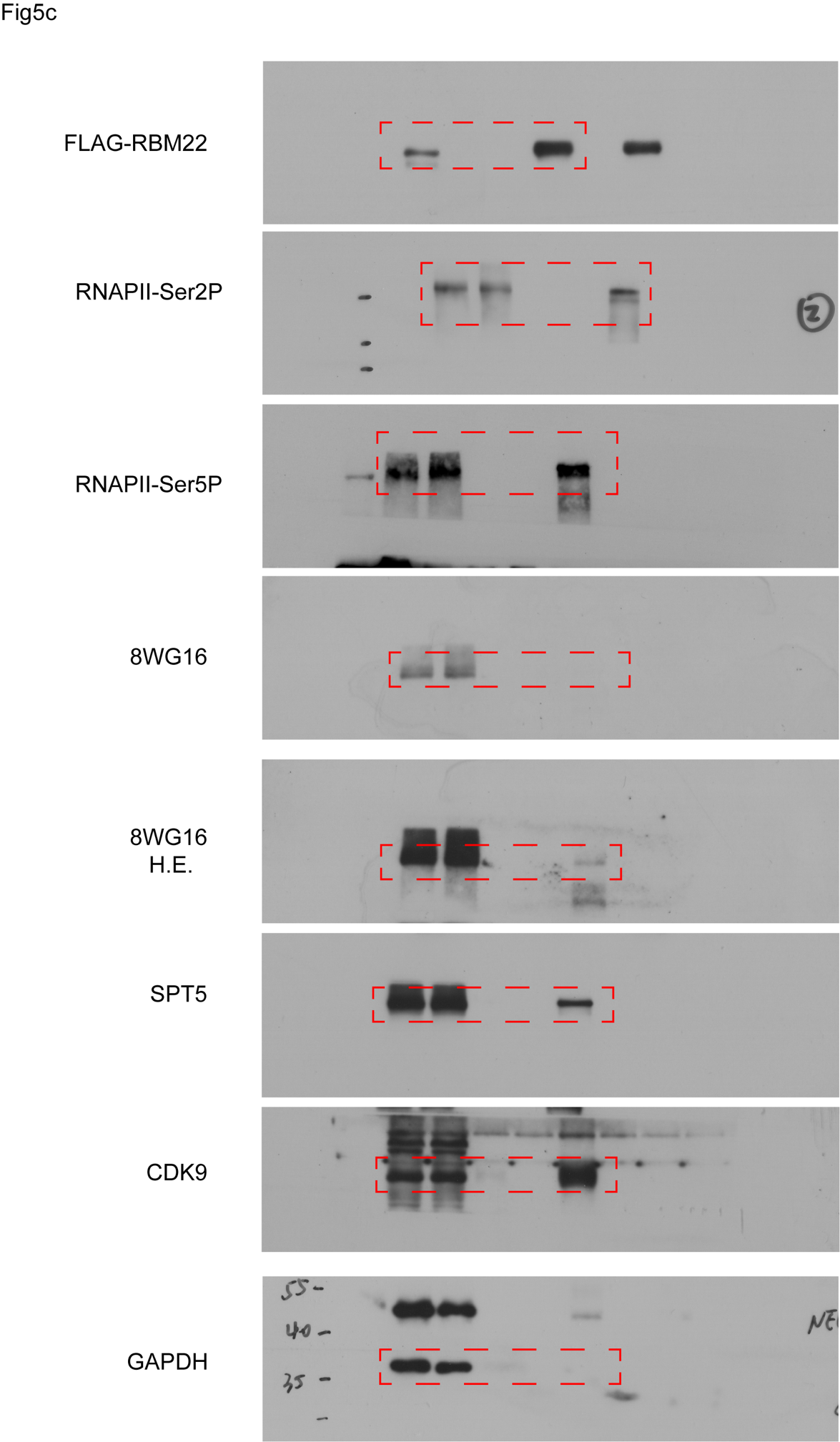
**

**Fig5.d**


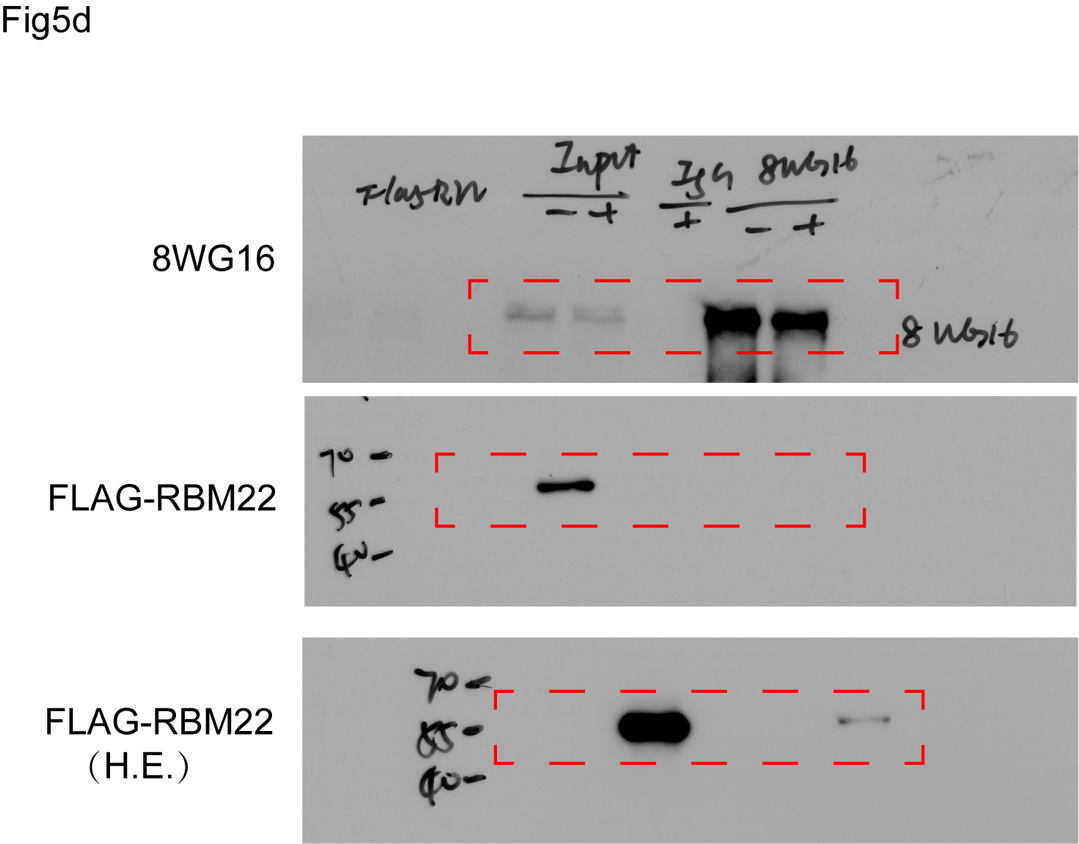


**Fig5.e**

**
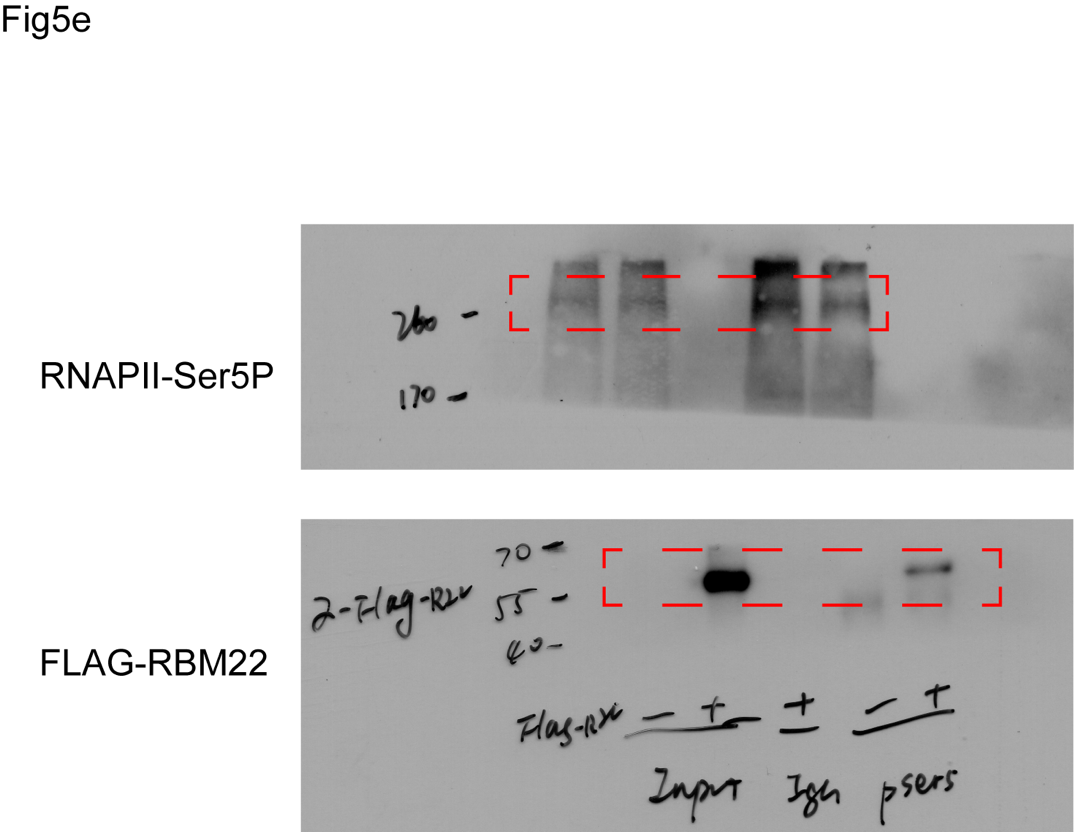
**

**Fig5.f**


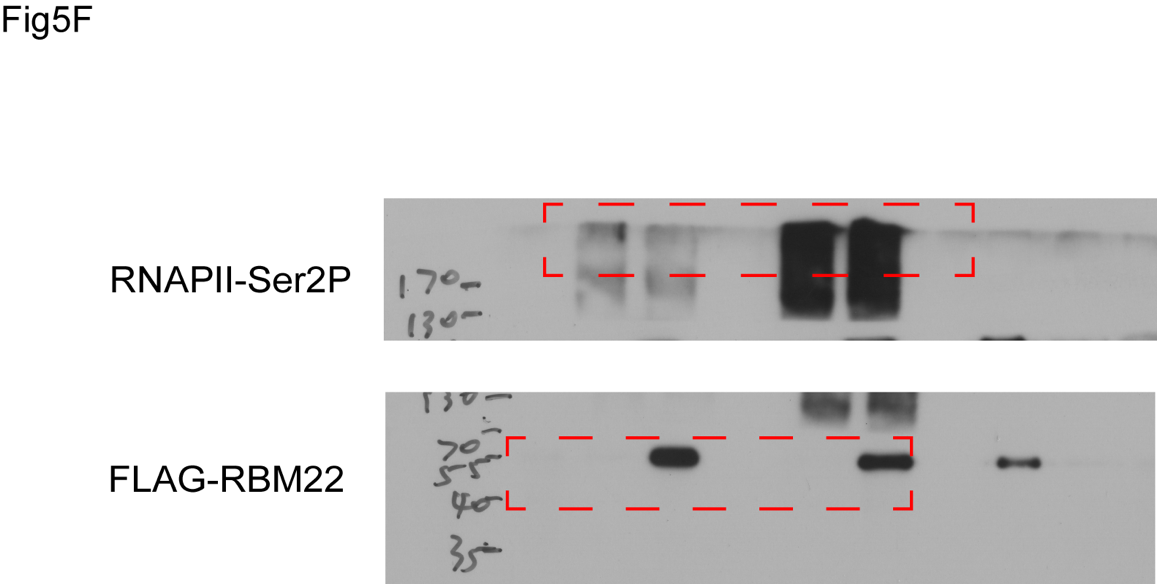


**Fig5.g**

**
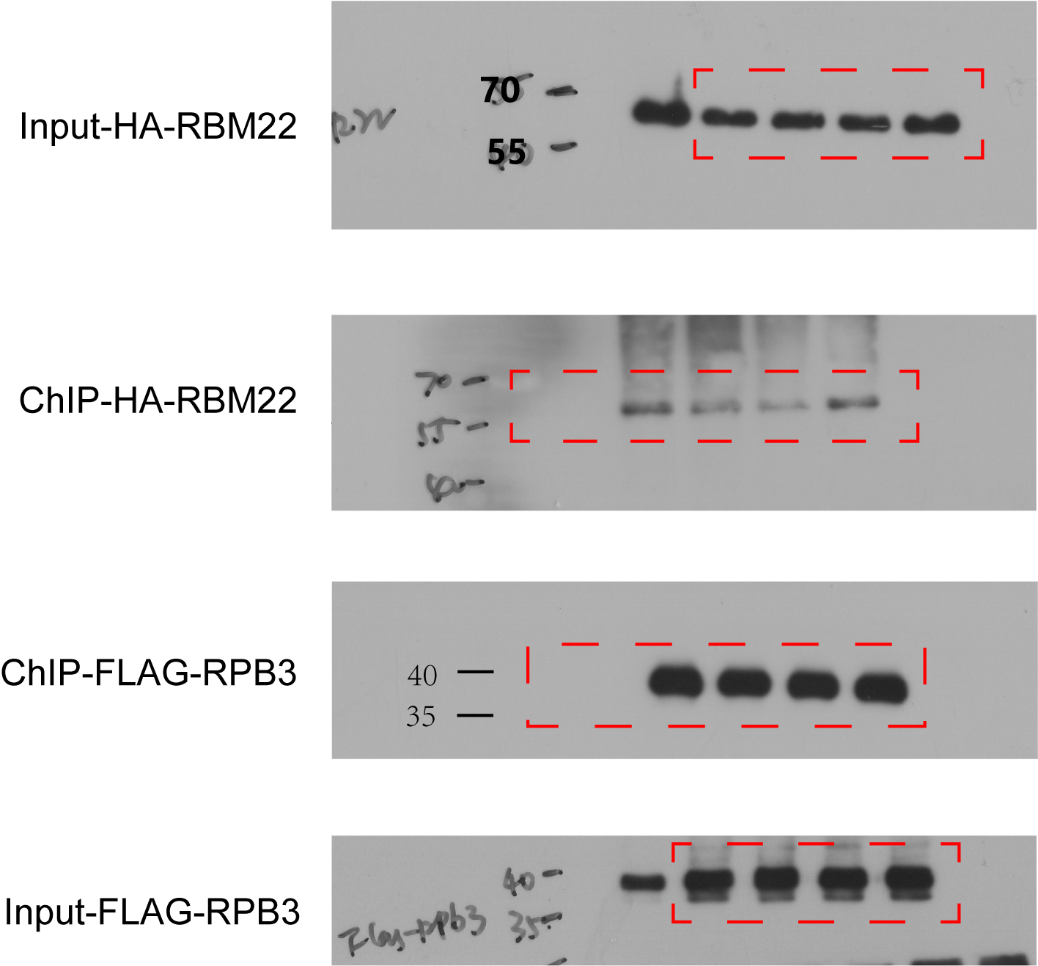
**

**Fig5.h**

**
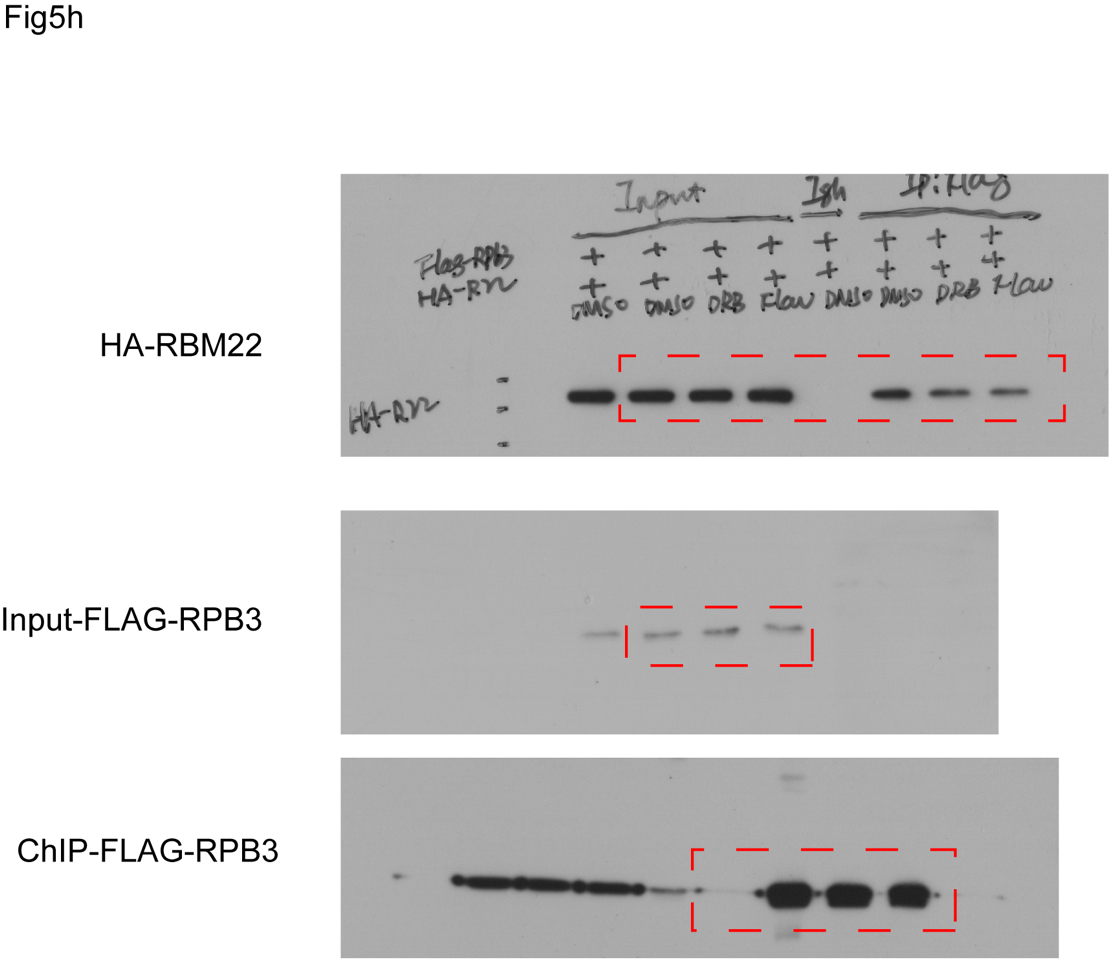
**

**Fig5.i**


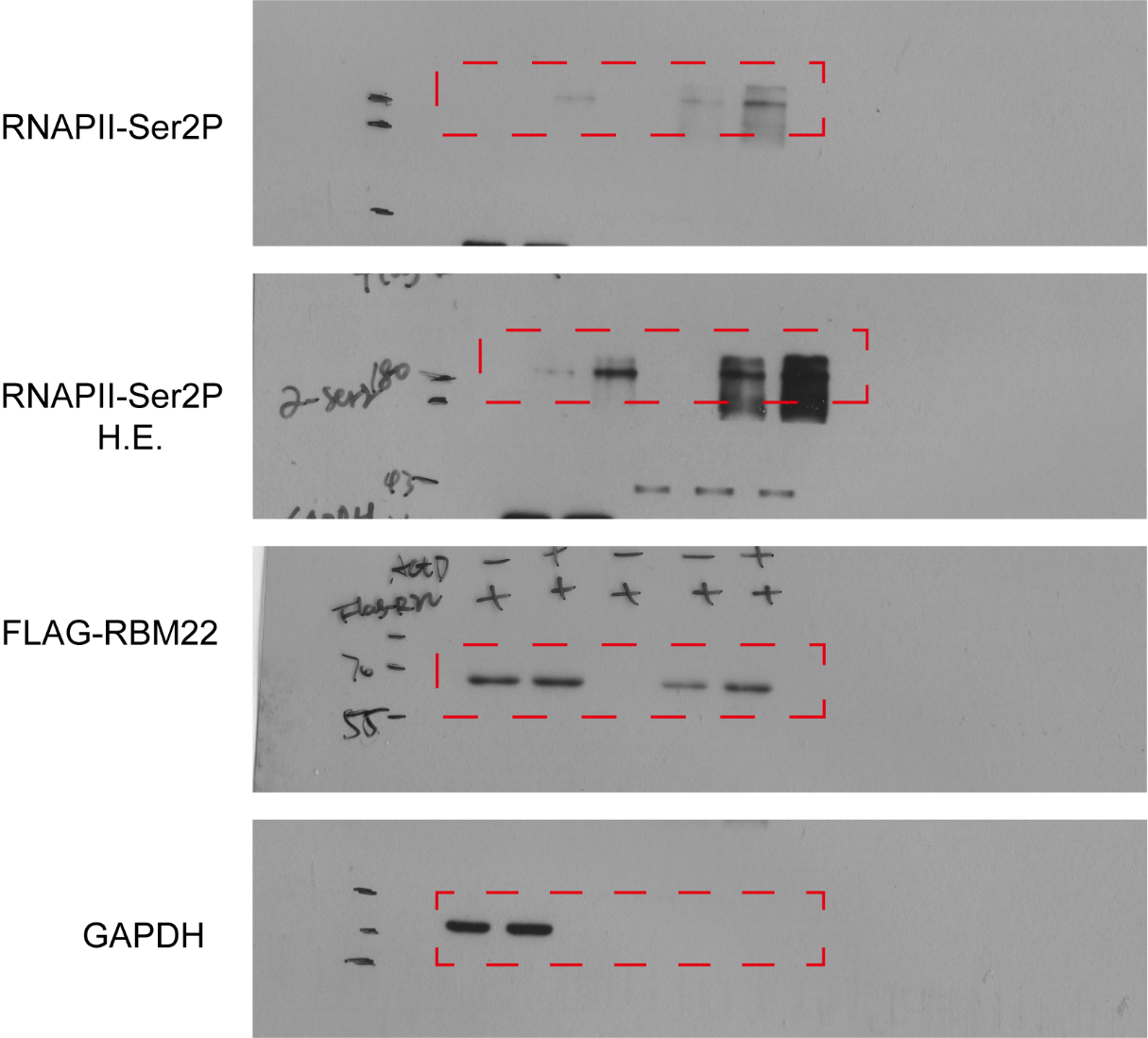


**Fig5.j**

**
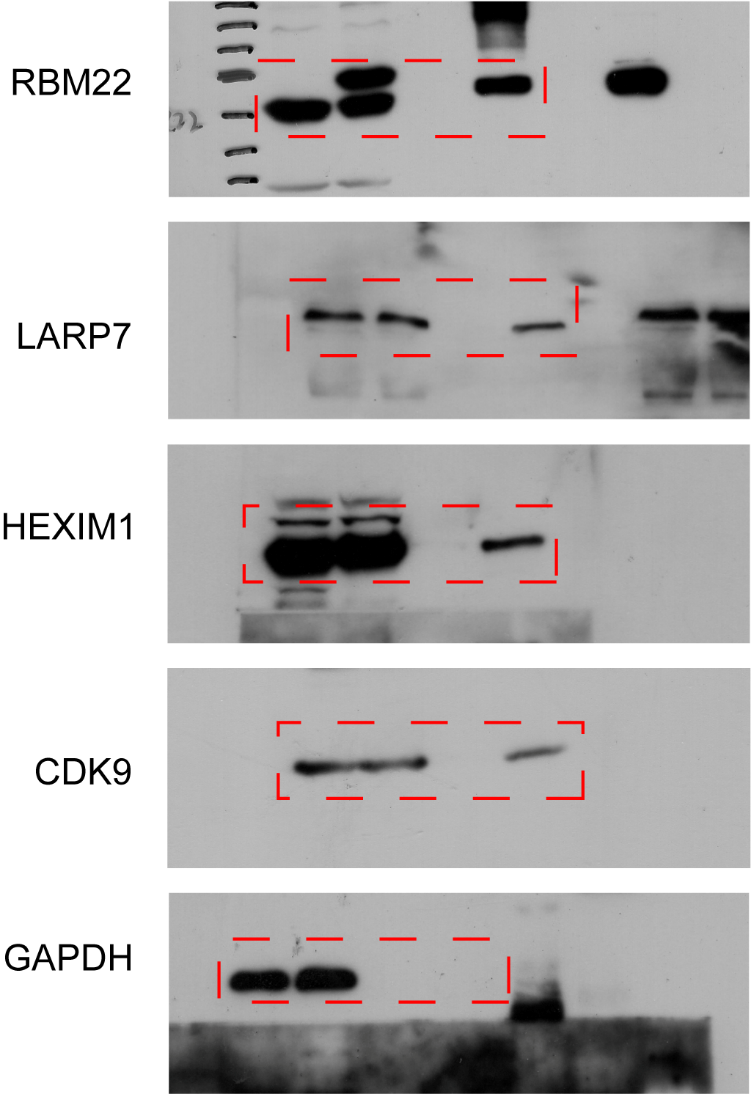
**

**Fig5.k**

**
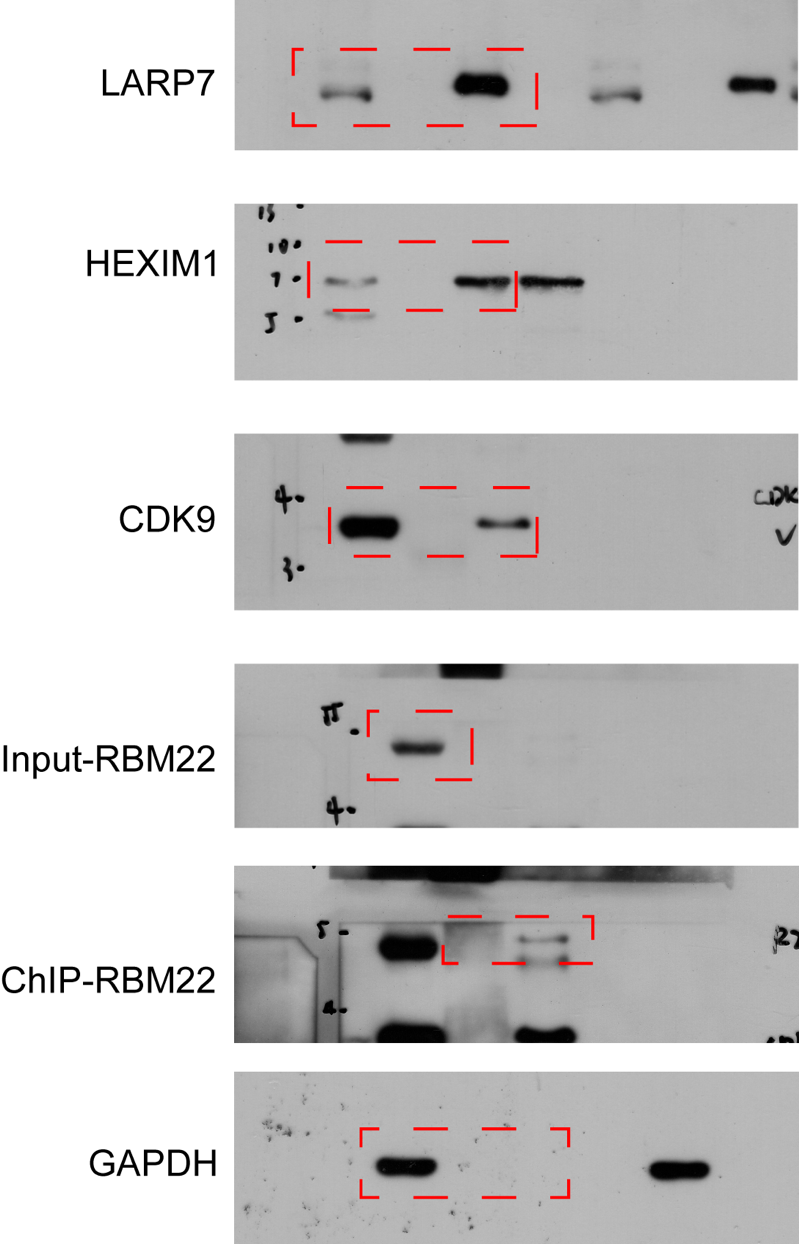
**

**Fig5.l**

**
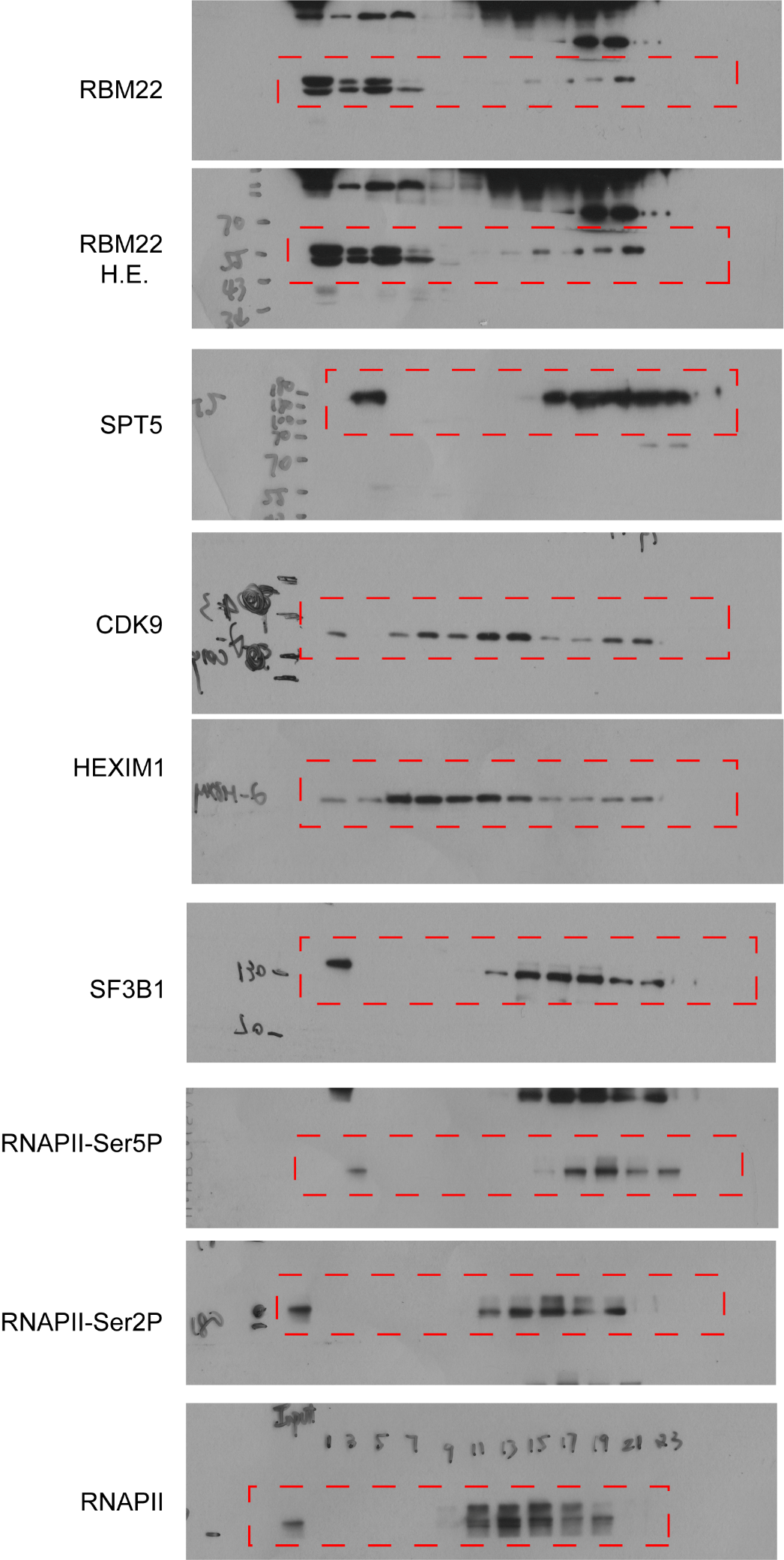
**

**Fig6.e**

**
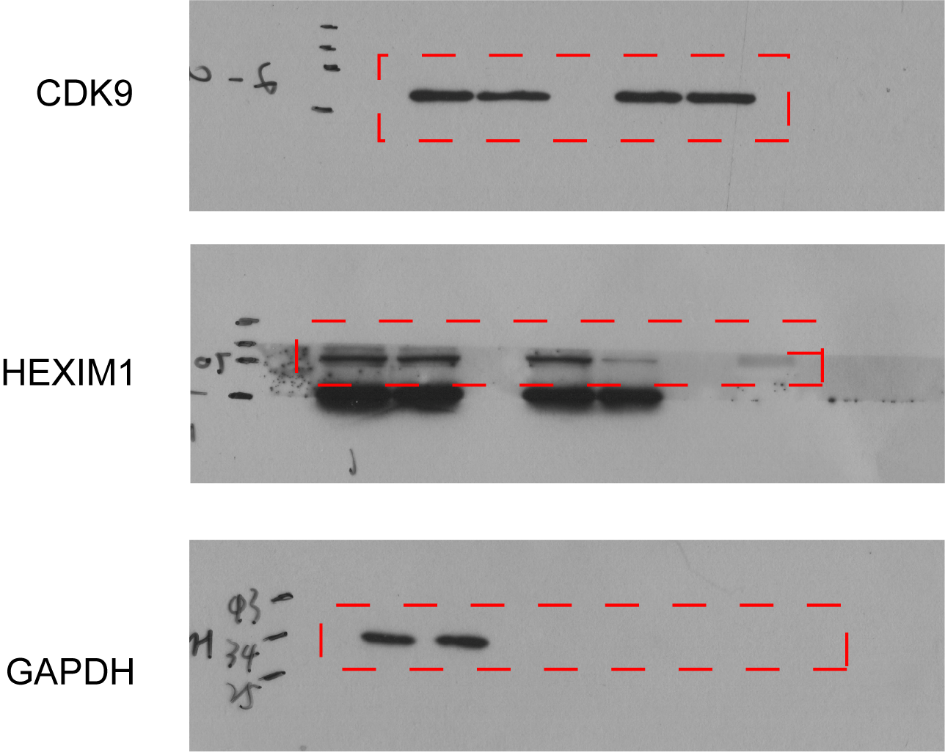
**

**Fig7.a**

**
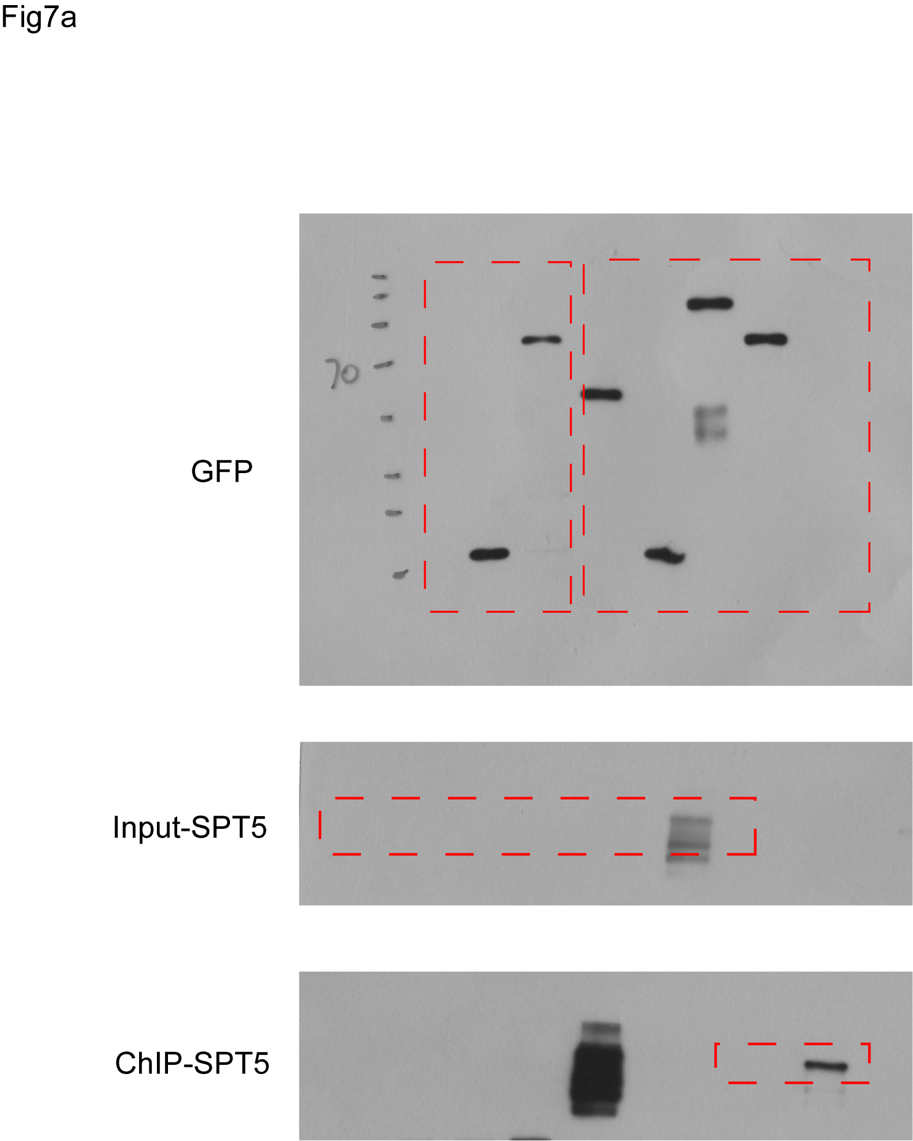
**

**Fig7.e**

**
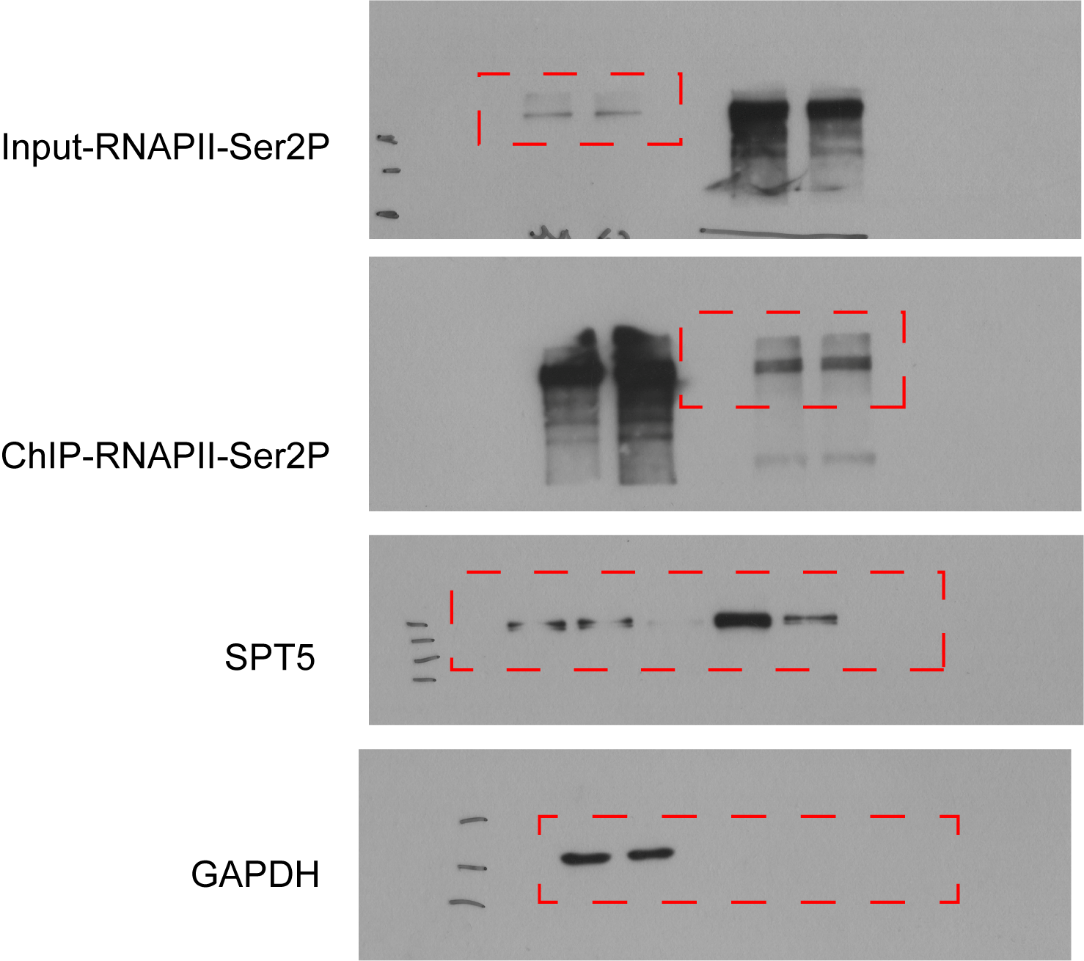
**

**Fig1S.h**

**
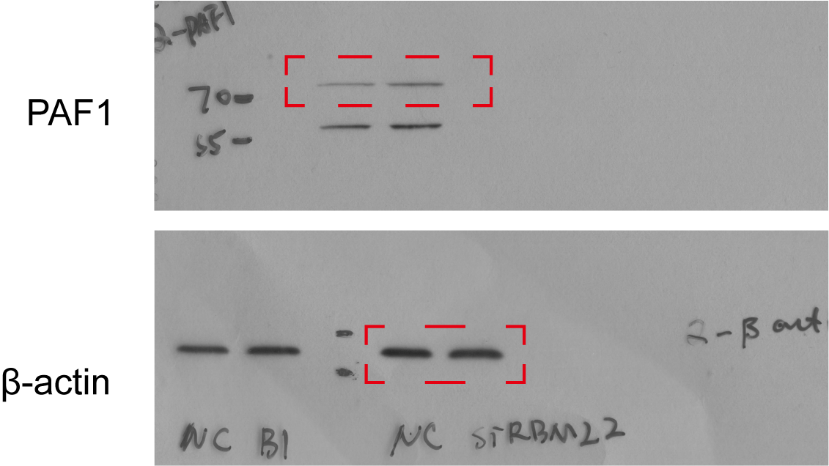
**

**Fig2S.a**

**
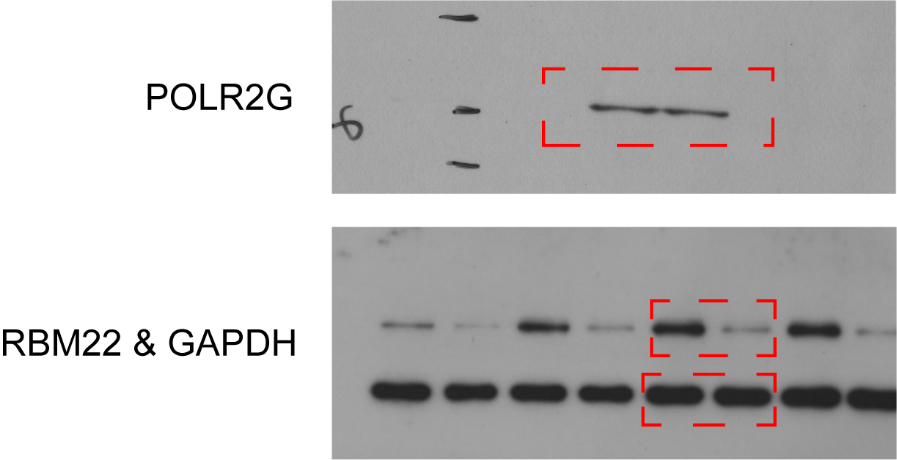
**

**Fig2S.f**

**
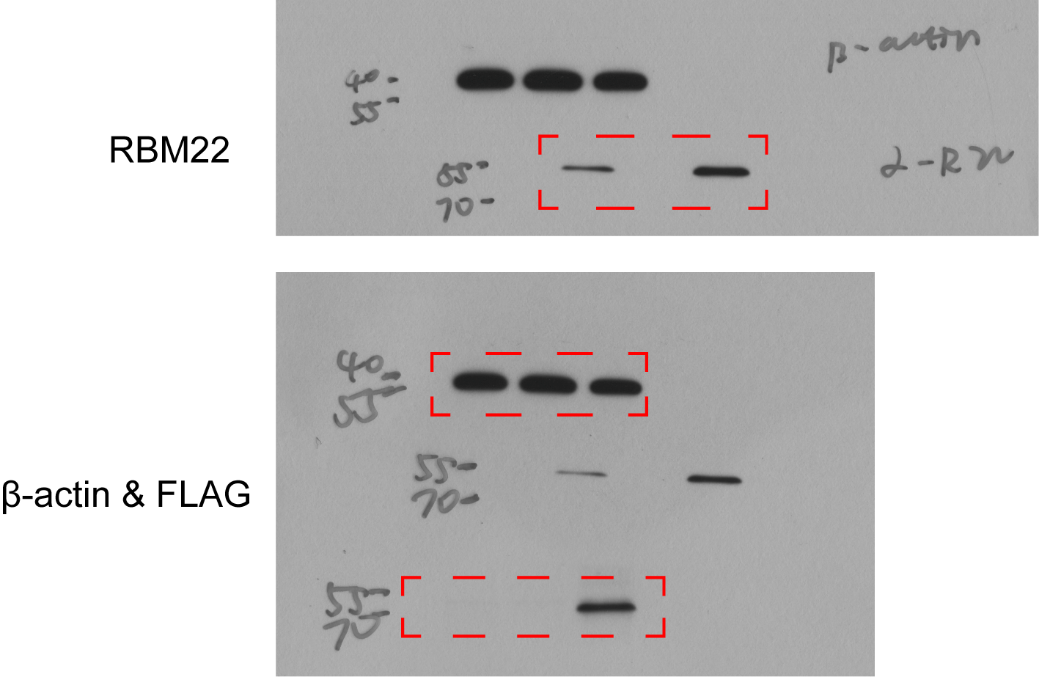
**

**Fig2S.j**

**
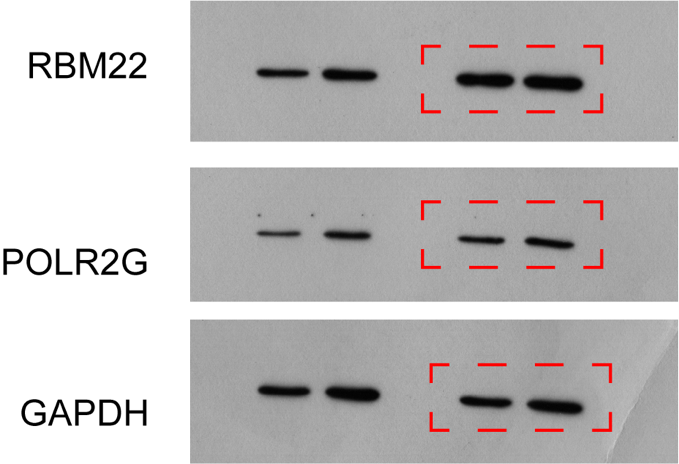
**

**Fig2S.n**

**
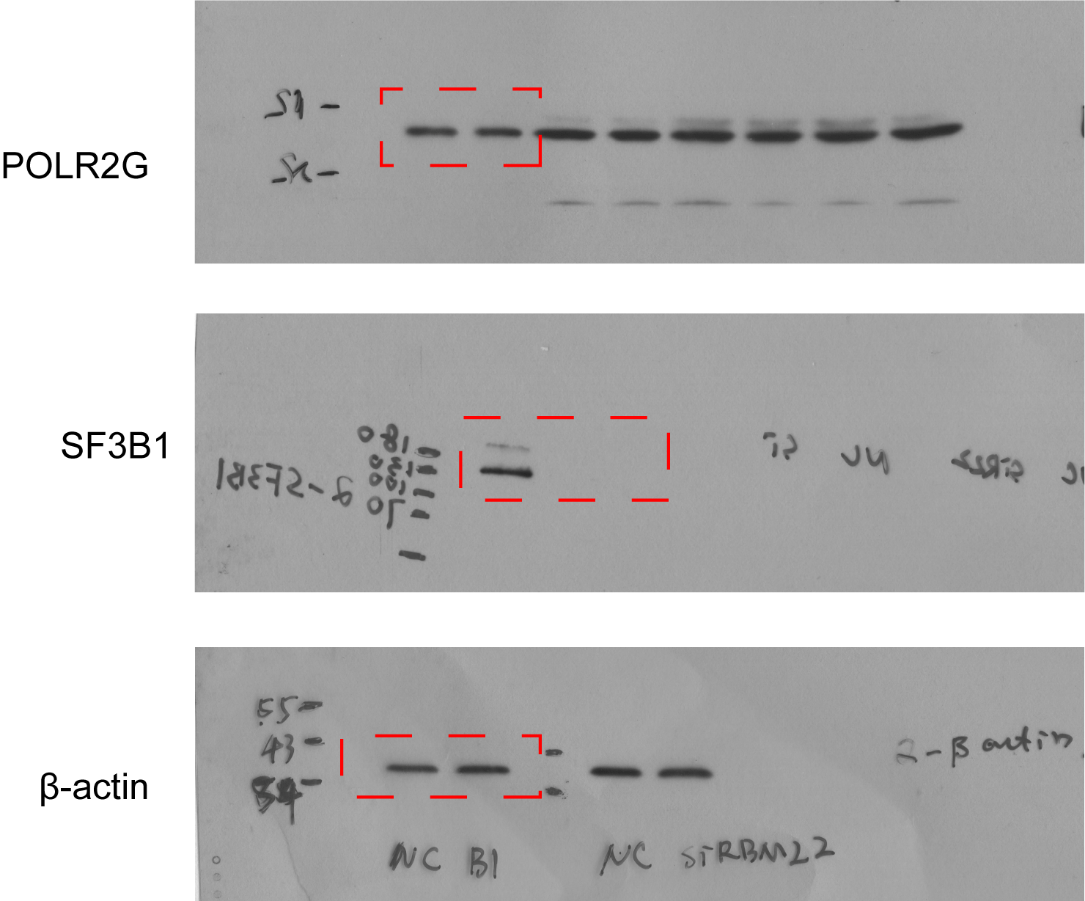
**

**Fig3S.b**

**
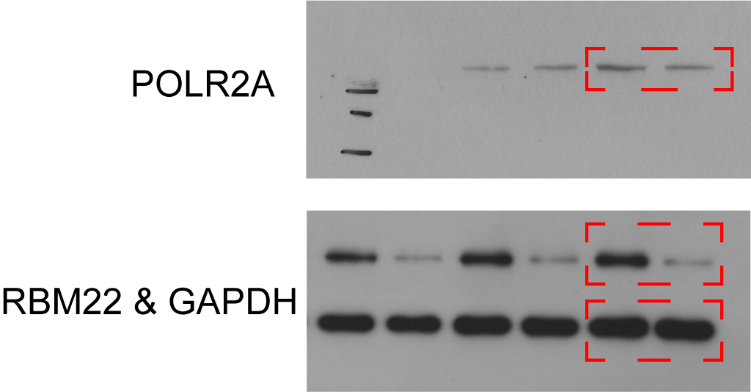
**

**Fig6S.c**

**
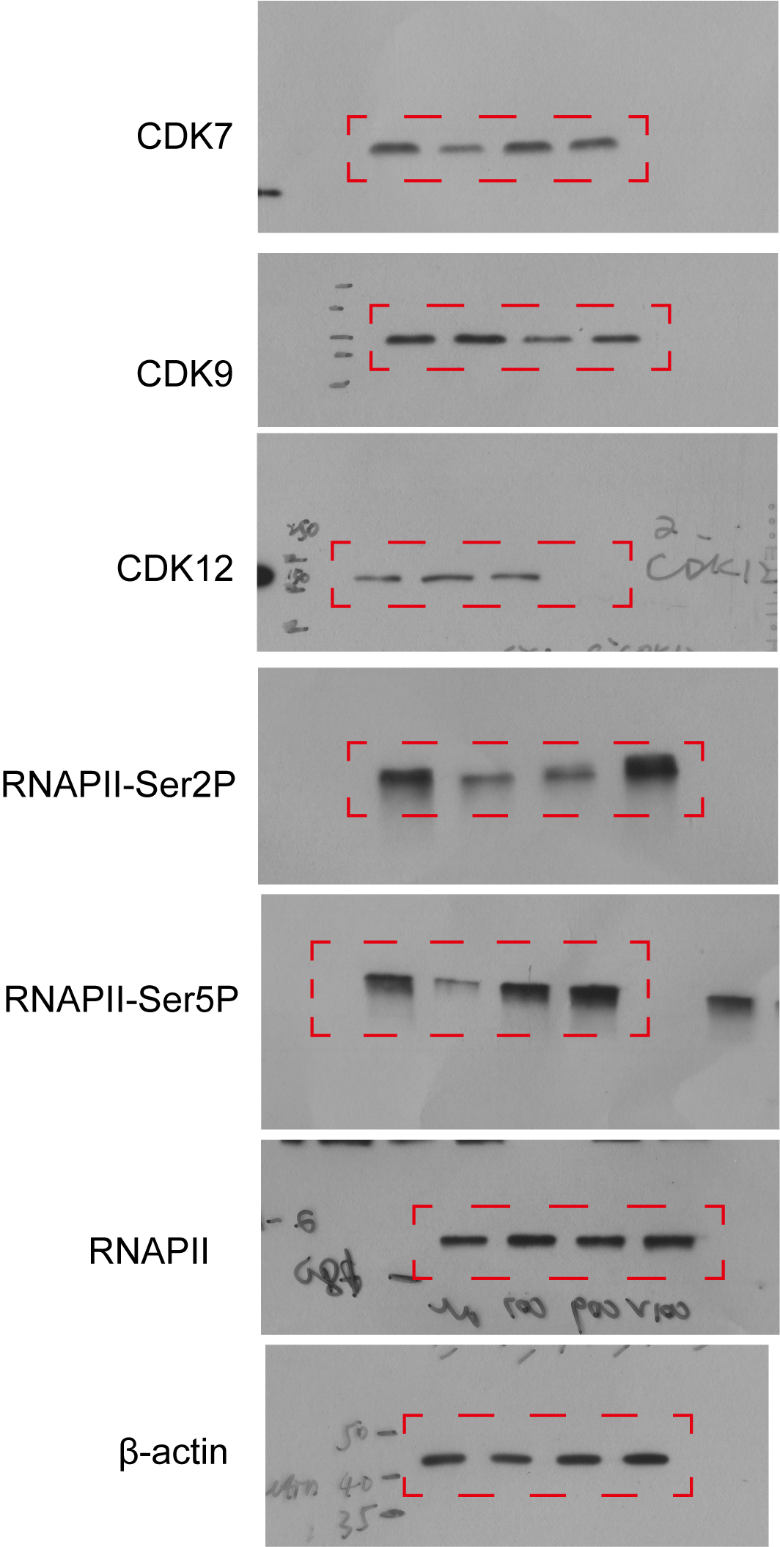
**

**Fig6S.d**

**
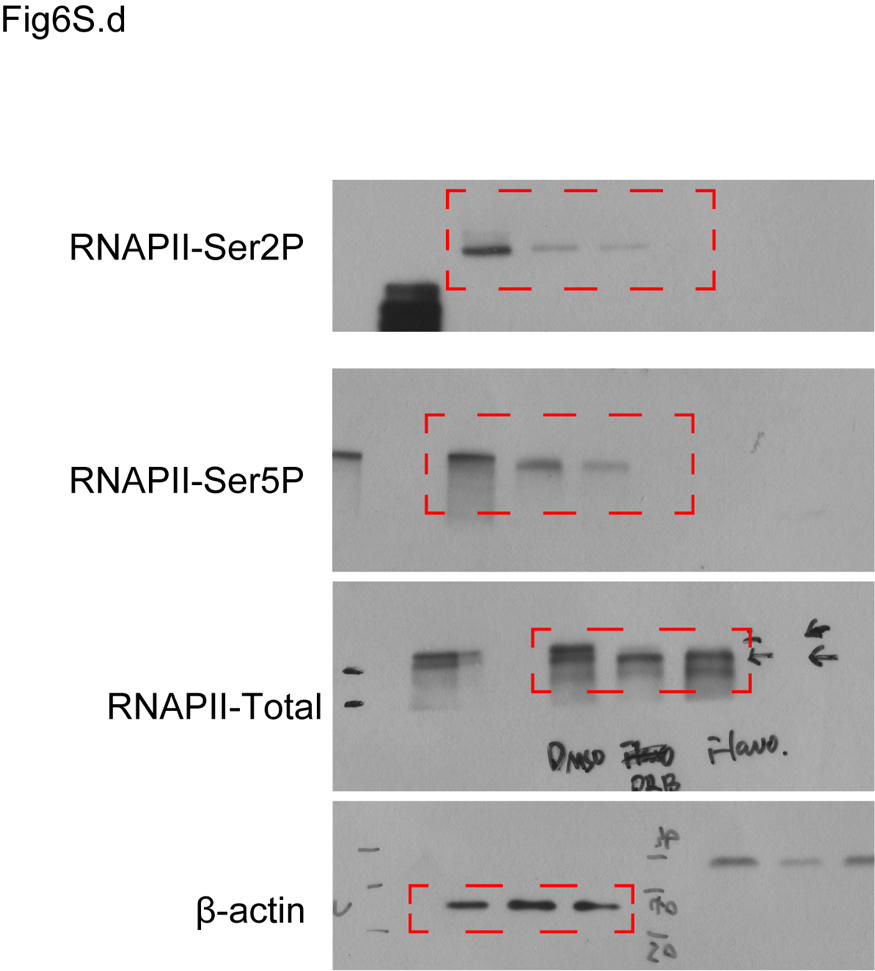
**

**Fig7S.c**

**
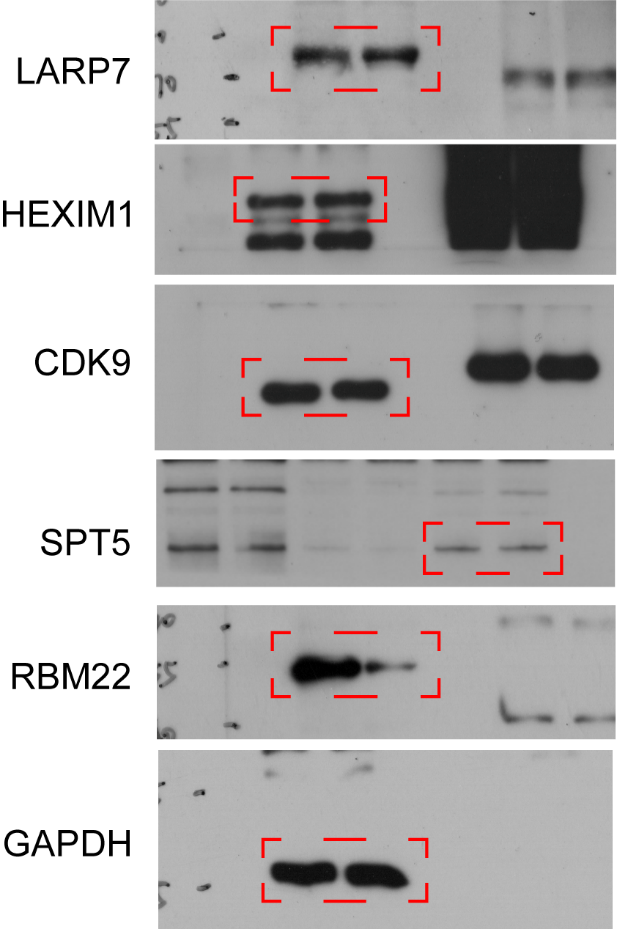
**

**Fig8S.i**

**
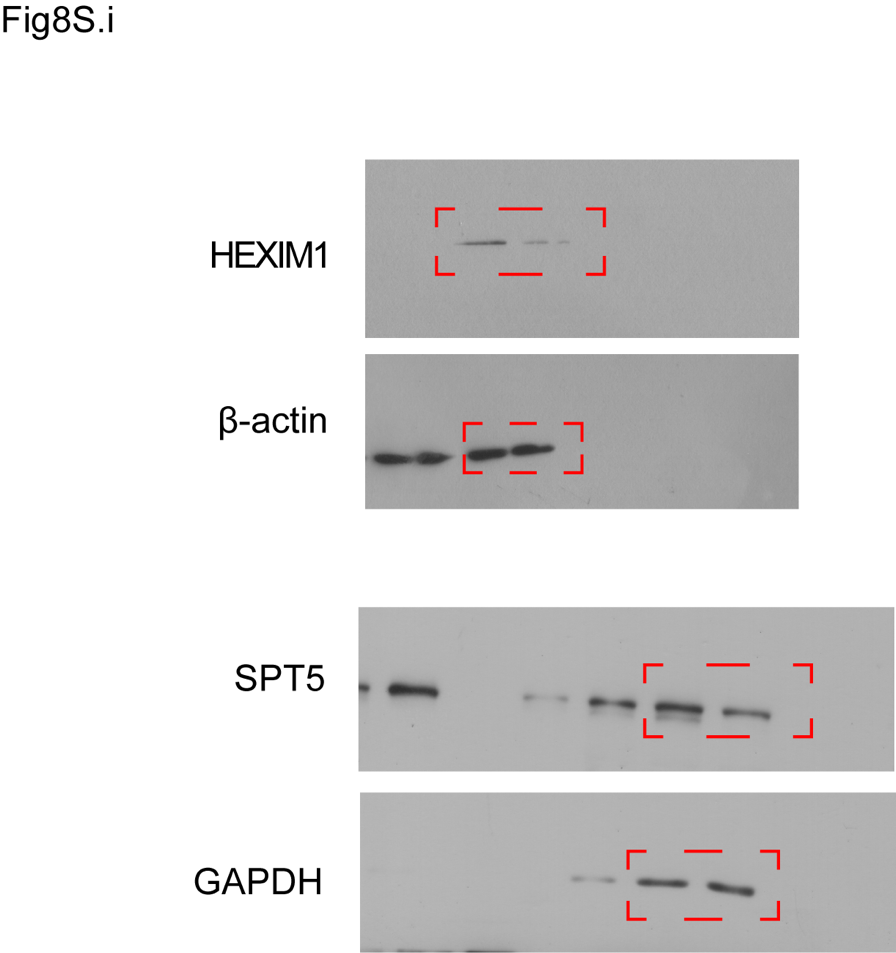
**
