# Supplementary material for: RBM22 regulates RNA polymerase II 5′ pausing, elongation rate, and termination by coordinating 7SK-P-TEFb complex and SPT5
Source: Genome Biol. 2024 Apr 19;25:102. doi: 10.1186/s13059-024-03242-6 (PMC11027413; doi:10.1186/s13059-024-03242-6)
Supplement: Supplementary file 3 — Additional file 3. [file 13059_2024_3242_MOESM3_ESM.docx]

**First round of review**

**Reviewer 1**

In the manuscript by Du et al., the researchers investigate the role of RBM22 in RNA polymerase II transcription. They provide compelling evidence that RBM22 knockdown affects Pol II transcription, as shown by analysis of Pol II distribution, nascent transcription, and elongation rate. Acute depletion of RBM22 also recapitulates these phenotypes. RBM22 forms physical associations with Pol II, 7SK-P-TEFb complex, and SPT5. Their model suggests that RBM22 represses Pol II pause release by binding and stabilizing the inhibitory 7SK-P-TEFb complex and regulates transcription elongation and termination through SPT5. The authors also describe RBM22's function in controlling transcription at snRNA and snoRNA genes, revealing a previously unidentified role of this splicing factor in transcription. While the conceptual framework and experimental design are persuasive, improvements are needed to enhance the manuscript's quality.

Major points:
1. Clarify whether the RNAPII elongation rate observed in Figure 2e (2 kb/min) is consistent with previous studies. Include relevant literature citations.

2. Explain why the authors used 415 genes for the analysis in Figure 2f while mentioning that 2217 genes longer than 80 kb were used to calculate the elongation rate (as stated in the Methods section, line 833).

3. In Figure 5, mention if any termination factors are present in the protein interactome of RBM22, considering its role in transcription termination along with the identified spliceosome components, transcription regulators, and elongation factors.

4. Address whether HEXIM1 is affected similarly to CDK9 pause release upon RBM22 depletion, as indicated in Figure 6f.

Minor points:

1. Replace "FLAG" with "α-FLAG" in Figure 5j.

2. Correct "SPT5 target/Not SPT5 target" to "SPT5 targets/non-SPT5 targets" in Figure 7b.

**Reviewer 2**

RNA-binding proteins are essential regulators at the post-transcriptional stage, such as RNA splicing. Unexpectedly, the study by Du et al. provides evidence that the splicing factor RBM22 may also contribute to transcription regulation on chromatin. Although the work is very interesting, the authors are suggested to address the concerns listed below before publication.

Fig. S1a,b - It is possible that RBM22 functions indirectly in transcription. Did knockdown of RBM22 affect the expression level/splicing of certain transcription factors?
Fig. S1c& line 113-115 - The reviewer disagrees with this explanation. How could RBM22 regulate transcription at the STEADY-STATE level?
Fig. 1b - Did the increased GRO-seq signals come from pre-mRNA accumulation? The authors may consider examining the overlap between differentially expressed genes from the GRO-seq data and differential splicing genes from the RNA-seq data.
Fig. S1e - Please include the rationale of examining snoRNA and snRNA in your main text. Clearly, RBM22 occupancy on snoRNA and snRNA genes was not identical to that on protein-coding genes.
Fig. 1c - It is an interesting and important observation, but pol II density only showed a very mild increase downstream of TSS. To further support the conclusion, the authors may consider showing the correlation between pol II and RBM22 density at TSS, gene body and TES.
Fig. 1e - (i) what about gene sets with different expression levels? It seems that genes with higher pausing are simply highly-expressed genes. (ii) How many genes in each group? Same concerns for 2g, 2h, 6c, 7d, etc......
Fig. S1m - Since the PRR ratio is an algorithm using an absolution position relative to TSS/TES (S1h), the difference between intron-less and intron containing genes in the genomic length may affect the result.
Fig. 1 and Fig. S1 - knockdown of components of U1/U2 snRNP can be a good control for ChIP experiments described in Fig.1 and S1.
Fig. 1i - It is interesting that uaRNA signals downstream of TSS were also increased. This may suggest that RBM22 functions by controlling chromatin conformation near TSS? The authors may include this in the discussion section.
Fig. 2 - It is very odd that RBM22 arrests pol II near TSS, but unexpectedly promotes rapid pol II elongation. The authors should provide a hypothesis for the conflicting functions of RBM22 in transcription regulation.
Fig. 2d - the pol II signals were also reduced in the upstream region (Primer1, pausing site?) upon RBM22 knockdown. Why? It seems that RBM22 enhances the overall binding of pol II to the genome. But this assumption is not consistent with the conclusion from Fig. 1.
Fig. 2f - Please include the rationale of selecting the 415 genes in the analysis. What about other genes?
Fig. 2g, h - Did the three gene sets belong to the 415 genes described in Fig. 2f?
Fig. S2h - is there a difference in the pol II elongation rate between intron-less and intron-containing genes?
Fig. 3 - please define active genes (e.g. line 266) and include the number of genes analyzed (e.g. 3b-d).
Fig. S3d - is there a difference in the level of DoGs between intron-less and intron-containing genes?
Fig. 3g and line 296-298 - sorry, I didn't get the point.
Fig. S3g and line 303-305 - the expression of DoG downstream genes was largely unaffected by RBM22 knockdown, which is inconsistent with the prior conclusion that RBM22 plays a general role in transcription regulation. RBM22-mediated pol II pausing does not occur at these downstream genes??? Why?
Fig. 4 - Again, please include the rationale of testing snoRNA and snRNA.
Fig. 4a, b - Pol II density around TSS was similarly increased upon RBM22 knockdown. This suggests that RBM22 does not function in paused pol II release and that RBM22 simply enhances the overall binding of pol II to the locus.
Fig. 4c and line 318-322 - I didn't observe a large increased readthrough signal right after TES (100 bp from TES).
Fig. S4k - Unlike regular DoGs, sRDoGs are unstable transcripts that might be subjected to quick degradation by nuclear quality control. The authors may consider providing a discussion about this phenotype.
Fig. 5b-f - Does the interaction depend on transcription? The author may consider performing the indicated coIP experiments (at least for the important one) using Actinomycin D-treated cells.
Fig. 5g and S5c - The authors concluded that CDK7 and CDK9 (but not CDK12) are required for the RBM22-polII interaction. But knockdown of CDK12 obviously upregulated the level of CDK7 as shown in S5c, which may enhance the interaction. These data somehow are not consistent. Why?
Fig. 5i, j - It is a great experiment that demonstrates the connection between RBM22 and the inhibitory 7SK complex. Since RBM22 is a RNA-binding protein, it is also important to determine whether RBM22 functions through its RNA-binding activity. The authors may consider (a.) checking the interaction between 7SK RNA and RBM22; (b.) performing the indicated coIP experiments using 7SK-KD or RNase A-treated samples.
Fig. 6a, b - It is odd that the binding of inhibitory and active components of 7SK-P-TEFb to the active loci exhibited a SIMILAR trend to each other upon RBM22 knockdown, which is also inconsistent with 6d. Actually, 6b showed a slight increase in HEXIM1 density downstream of TSS. Why?
Fig. 6f - Please perform a same analysis using the HEXIM1 and SPT5 ChIP data as controls.
Fig. 7a - Please see the comments for 5b-f and 5i, j.

Please carefully check the grammar errors.

**Reviewer 3**

Remarks to the authors:
The report by Du et al documents an activity by an established splicing factor and an RNA-binding protein (RBP), RBM22, in regulating multiple stages of gene transcription by RNA polymerase II (Pol II) in human cells. The authors provide several lines of evidence that the transcription-regulatory activity of RBM22, which, as reported, plays critical roles in the control of transcriptional pause release, elongation, and termination, is independent of its role in splicing. They also identify RBM22 as the first non-canonical component of the 7SK complex that promotes its inhibitory activity, thereby antagonizing Pol II pause release.
This is a well-designed and well-executed study with important implications for the fields of transcription and splicing. The study also makes an important new case for a general RBP in regulating gene transcription and, potentially, coordination of transcription with post-transcriptional processing. The main strength is discovery of new activity via genome-wide approaches and the key conclusions are sufficiently supported biochemically.
I congratulate the authors for their findings and have no serious concerns that would preclude publication of this study. However, I provide some recommendations, grouped below into major and minor comments, that might help clarify several points and should be fairly easy to address. I also recommend that the manuscript be edited for grammatical errors.

Major comments:

1) A critical finding of this work is that a splicing factor regulates transcription independent of its role in splicing. Since RBM22 is not the first such case, can the authors speculate on why splicing factors might be particularly well suited to 'moonlight' as transcriptional regulators? Some discussion of this would give the motivation for this work and its conclusions more context.

2) RBM22 presumably travels with Pol II as it regulates its activity. Since much of the pre-mRNA splicing occurs co-transcriptionally, one wonders if the same Pol II-associated molecule of RBM22 also facilitates splicing (e.g., by transiently becoming part of the spliceosome). While experiments to resolve this in detail may be challenging, it would nevertheless be important to understand whether the bulk of RBM22 forms just one large protein complex with Pol II and components of the spliceosome, or it forms separate complexes, one with Pol II and associated factors and one with the spliceosome. A relatively simple experiment to address this would be density (e.g. glycerol) gradient-based fractionation of cell lysates followed by western analysis of protein subunits that are specific to each machinery. Such analysis might also find RBM22-7SK complex as a separate species. Since this could provide support for - and clarify - the key arguments about coordination of different Pol II activities and splicing, I recommend that the authors give this a try. All the necessary antibodies are at hand.

3) Approximately 30%-40% of metazoan genes are regulated via promoter-proximal Pol II pausing, particularly genes participating in signal-responsive pathways (see e.g. Adelman & Lis, Nat Rev Genet 2012). Pol II pausing at only a fraction of all paused genes is regulated by the inhibitory 7SK complex. However, the authors report on the role of RBM22 in controlling Pol II pause release "at most gene promoters". I suggest that this be better explained, given that most genes are likely not regulated by pausing. Are the genes known to be regulated by the 7SK complex (e.g. study in ESCs by Castelo-Branco et al, Genome Biol 2013) particularly strongly regulated by depletion of RBM22? Is regulation by RBM22 via 7SK likely to be mandatory for the control of pausing? Also, are genes whose pausing is strongly regulated by RBM22 enriched in any specific gene ontology (GO) categories?

4) I suggest that the authors demonstrate the specificity of their RBM22 ChIP signal using either siRBM22 or inducible degradation of mAID-RBM22. A ChIP-qPCR experiment should suffice.

5) On the positive correlation between elongation velocity and RBM22 occupancy (Fig. 2g): can the authors speculate on what recruits RBM22 to genes with higher elongation velocity? I recommend that the same bins of genes (low/medium/high elongation rates) be analyzed for intron density, Pol II S5P vs S2P, and SPT5 occupancy.

6) How do the authors explain the observation that Pol II pause release is much more efficient upon RBM22 knockdown by siRNA (which is good but incomplete; Fig. S1f) than after acute loss of RBM22 (which is essentially complete; compare Figs. S1k and 1g)?

7) Readthrough index (R1) uses as the numerator GRO-seq read density in the region from TES to the TSS of a downstream gene (Fig. S3b). I find this problematic since this distance may vary, depending on the location of a gene, by several orders of magnitude. Instead, a fixed distance 3' of TES should be defined and used to calculate R1.

8) It is not immediately obvious why the authors liken the readthrough seen upon loss of RBM22 specifically to stress-induced transcriptional readthrough observed by Steitz and colleagues (lines 267 and 301/302)? With numerous other reported cases of induced transcriptional readthrough, is there anything that makes osmotic stress-induced readthrough particularly "reminiscent" of the current results? A comparison is made between the functions of "DoG-producing" genes in this and the Steitz's study, and a difference is found, but what is the relevance of this finding? What does the enrichment of genes with roles in viral gene expression imply? This should be either clarified or removed.

9) In what sense are readthrough transcripts downstream of sno/snRNA genes of a "new type" (chapter starting with line 341)? The authors find that these transcripts are not polyadenylated, but are DoG transcripts of protein-coding genes polyadenylated?

10) It is stated that Ser5 and Ser2 phosphorylation was "eliminated" by siRNA-mediated KD of CDK7, CDK9, and CDK12 (Fig. 5; lines 389-391), but the KD efficiency of CDK7 and CDK9 is quite weak, maybe 60% (Fig. S5c), unlikely to suffice for a very significant reduction in phosphorylation. It would help if the authors showed the actual remaining levels of phosphorylation, as they do (and that is convincing) for the use of inhibitors (Fig. S5d). In addition, text should clarify what KD of each CDK7, CDK9, and CDK12 is expected to do and why a very good KD of CDK12 shows no effect on Pol II - RBM22 association.


Minor comments:

- The way that NGS data were normalized should be included in the methods section.

- Please cite figure panels in their alphabetical order. Cite S1d before S1e (line 128), cite Figs. 6c and 6d before 6e (line 417).

- All co-IP/western experiments should state what % of total material was loaded in "input" and what % was loaded in "IP" lanes. This is important to gauge the efficiency of co-IP.

- Were the co-IP/western experiments in Figs. 5i and 5j done in the presence of RNase? Do the authors expect that the association of RBM22 with the 7SK protein components relies on RBM22 - 7SK ncRNA binding?

- Line 161, correct Fig 1Sl to Fig S1l

- Line 226: the reference, I believe, should be to Fig. 2d, not 3d.

- Line 233 and Fig. 2f: which 415 genes were analyzed here?

- Line 298: increased "intensity" of DoG transcripts should be replaced with "expression".

- In Fig. S5b, what does log2FC(+Dox/-Dox) pertain to? Fold change (FC) of what? This should be explained in the figure legend.

- Line 417: "Activating" would seem better than "active".

- Line 435: Cite literature documenting CDK9-HEXIM1 interaction by co-IP.

- Fig. 6f, y-axis: are these numbers of genes in thousands? Please fix.
- Fig. 6h: what are the yellow lines for? It should not be necessary to have two legends.

- Lines 470-472: the conclusion should mention RBM22.

**Point-to-point Response to Reviewers:**

**Reviewer #1:**

In the manuscript by Du et al., the researchers investigate the role of RBM22 in RNA polymerase II transcription. They provide compelling evidence that RBM22 knockdown affects Pol II transcription, as shown by analysis of Pol II distribution, nascent transcription, and elongation rate. Acute depletion of RBM22 also recapitulates these phenotypes. RBM22 forms physical associations with Pol II, 7SK-P-TEFb complex, and SPT5. Their model suggests that RBM22 represses Pol II pause release by binding and stabilizing the inhibitory 7SK-P-TEFb complex and regulates transcription elongation and termination through SPT5. The authors also describe RBM22's function in controlling transcription at snRNA and snoRNA genes, revealing a previously unidentified role of this splicing factor in transcription. While the conceptual framework and experimental design are persuasive, improvements are needed to enhance the manuscript's quality.

We highly appreciate the Reviewer’s recognition of importance of our work and positive comments. Below, we addressed the issues raised by the Reviewer.

Major points:

1. Clarify whether the RNAPII elongation rate observed in Figure 2e (2 kb/min) is consistent with previous studies. Include relevant literature citations.

We appreciate the Reviewer’s suggestion. In the control cells, we noted an estimated Pol II elongation rate of approximately 2 kb/min, consistent with findings reported in previous studies [1, 2]. We have provided clarification and appropriately cited the relevant literature in the revised manuscript.

2. Explain why the authors used 415 genes for the analysis in Figure 2f while mentioning that 2217 genes longer than 80 kb were used to calculate the elongation rate (as stated in the Methods section, line 833).

To compare Pol II elongation rate at individual gene level, we deliberately selected 415 genes with robust Pol II ChIP-seq signals at all three time points in both control and RBM22-depleted cells for calculation of Pol II elongation rate. The remaining genes did not meet these strict criteria. The rationale for selecting these specific 415 genes has been included in the revised manuscript.

We selected 2217 genes longer than 80 kb with non-overlapping transcription units were for the metagene analysis in Fig. 2b. To increase inclusivity, we have relaxed the criteria, as detailed in the revised manuscript, and emphasized this difference.

3. In Figure 5, mention if any termination factors are present in the protein interactome of RBM22, considering its role in transcription termination along with the identified spliceosome components, transcription regulators, and elongation factors.

We have meticulously examined the protein interactome of RBM22 and confirmed the absence of the termination factor.

4. Address whether HEXIM1 is affected similarly to CDK9 pause release upon RBM22 depletion, as indicated in Figure 6f.

We appreciate the Reviewer’s suggestion. We performed a same analysis using the HEXIM1 ChIP data, as shown in Fig. R1. Unlike CDK9 accumulation (Fig. 6f), HEXIM1 showed a decrease in accumulation before the +1 nucleosome, with only a subtle increase after the +1 nucleosome, consistent with its disassociation from the promoter (Fig. 6b). To enhance clarity, we have incorporated this control as a new Fig. S7d in the revised manuscript.


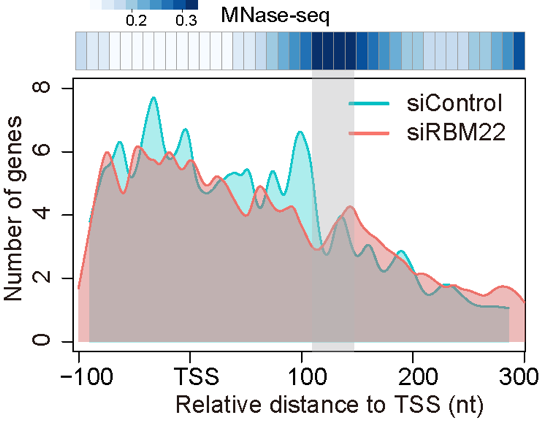


**Fig. R1.** Distance distribution of HEXIM1 ChIP-seq peak summit relative to TSS and +1 nucleosome dyads. The +1 nucleosome position is determined by MNase-seq. The y axis represents the gene number of HEXIM1 accumulation at relative positions from summit to TSS in control and RBM22 knockdown cells.

Minor points:

1. Replace "FLAG" with "α-FLAG" in Figure 5j.

We have fixed it accordingly.

2. Correct "SPT5 target/Not SPT5 target" to "SPT5 targets/non-SPT5 targets" in Figure 7b.

We have fixed it accordingly.

**Reviewer #2:**

RNA-binding proteins are essential regulators at the post-transcriptional stage, such as RNA splicing. Unexpectedly, the study by Du et al. provides evidence that the splicing factor RBM22 may also contribute to transcription regulation on chromatin. Although the work is very interesting, the authors are suggested to address the concerns listed below before publication.

The Reviewer highlights the unexpected finding regarding the involvement of the splicing factor RBM22 in transcriptional regulation on chromatin in our study. Below, we addressed the concerns raised by the Reviewer.

Fig. S1a,b - It is possible that RBM22 functions indirectly in transcription. Did knockdown of RBM22 affect the expression level/splicing of certain transcription factors?

The Reviewer expressed concern about the possibility of RBM22 indirectly regulating transcription by modulating expression level or splicing of certain transcription factors. Firstly, as illustrated in Fig. S7c, the protein levels of key regulatory transcription factors, including LARP7, HEXIM1, CDK9, and SPT5, remained unchanged upon RBM22 knockdown. Furthermore, upon re-evaluating differentially expressed genes and differential splicing genes in Fig. S1a-b, we observed no significant alternations in the expression level (Fig. R2a) as well as splicing patterns (Fig. R2b) of the vast majority of documented regulatory transcription factors[3-9] following RBM22 knockdown. Notably, only *PAF1* showed a marginal expression change (log2FoldChange =0.66). To ensure accuracy, we further confirmed its unchanged protein expression upon RBM22 knockdown through western blotting, as illustrated in Fig. R2c.

Please note that we have already performed acute depletion of RBM22 (Fig. 1f), successfully recapitulating the observed phenotypes and thereby ruling out the possibility of an indirect effect, as depicted in Fig. 1g, 2d and 3f. Consequently, it is unlikely that RBM22 indirectly regulates transcription through modulating the expression level or splicing of specific transcription factors. We have incorporated these new findings as new Fig. S1f-h and emphasized this point in the revised manuscript for clarity.


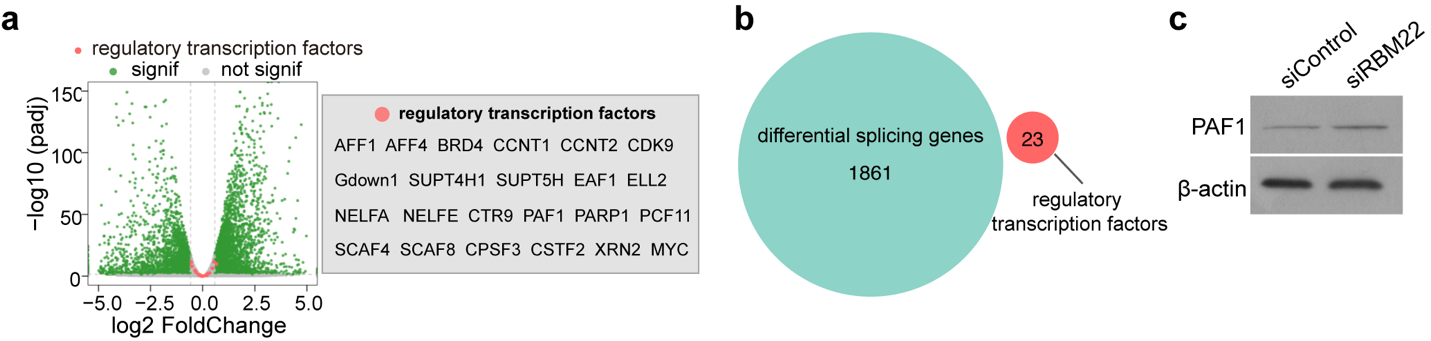


**Fig. R2.** Limited effect of RBM22 knockdown on the expression level or splicing of certain transcription factors. (a) Volcano plot showing the changes in gene expression profiled by RNA-seq (left) and a list of documented regulatory transcription factors (right). Significantly regulated genes are determined by padj of < 0.05 and log2FoldChange of < -0.58 or > 0.58. (b) Venn diagram showing the overlap of differential splicing genes and the certain regulatory transcription factors. (c) Efficient knockdown of RBM22 without affecting the PAF1 protein.

Fig. S1c& line 113-115 - The reviewer disagrees with this explanation. How could RBM22 regulate transcription at the STEADY-STATE level?

We apologize for the inaccurate description. We have made changes accordingly by removing “at the steady state” and substituting “RNA level” with “mRNA level” in the revised version.

Fig. 1b - Did the increased GRO-seq signals come from pre-mRNA accumulation? The authors may consider examining the overlap between differentially expressed genes from the GRO-seq data and differential splicing genes from the RNA-seq data.

The Reviewer might mention the GRO-seq signals in Fig. 1h, not in Fig. 1b. Following the reviewer’s suggestion, we have conducted a thorough analysis and identified a very limited subset of genes (265 genes) that exhibited overlap between differentially expressed genes from the GRO-seq data and differential splicing genes from the RNA-seq data (Fig. R3a). This finding suggests that the GRO-seq signals at these particular genes may be potentially affected by pre-mRNA accumulation. Furthermore, upon closer examination, we observed a significant downregulation in the GRO-seq signals for these 265 overlapped genes (Fig. R3b). Consequently, the increased GRO-seq signals depicted in Fig. 1h does not arise from pre-mRNA accumulation.


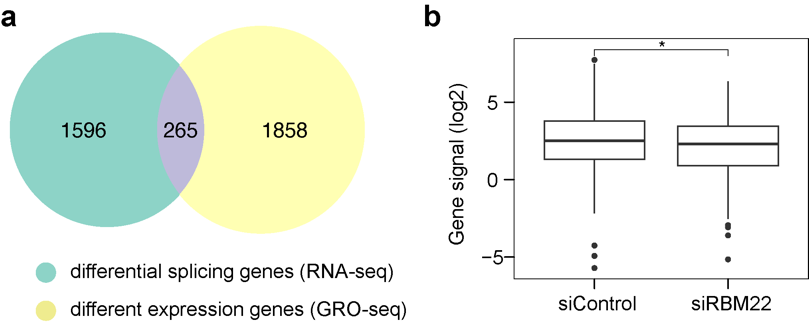


**Fig. R3.** (a) Venn diagram showing the overlap of differential splicing genes quantified by RNA-seq and different expression genes quantified by GRO-seq. (b) Boxplot displaying the decreased nascent RNA signals at 265 genes in response to RBM22 knockdown in HepG2 cells. The p value was determined using the wilcoxon test

Fig. S1e - Please include the rationale of examining snoRNA and snRNA in your main text. Clearly, RBM22 occupancy on snoRNA and snRNA genes was not identical to that on protein-coding genes.

We appreciate the Reviewer's insightful comment. The examination of snoRNA and snRNA in our study stems from the recognition that RBM22 occupancy on these non-coding RNA genes may exhibit distinct patterns compared to protein-coding genes. Understanding the regulatory interactions between RBM22 and non-coding RNAs is essential for comprehensively characterizing its functional role. We have incorporated a rationale for investigating snoRNA and snRNA in the revised manuscript, highlighting the potential differences in RBM22 occupancy and its implications on gene regulation.

Fig. 1c - It is an interesting and important observation, but pol II density only showed a very mild increase downstream of TSS. To further support the conclusion, the authors may consider showing the correlation between pol II and RBM22 density at TSS, gene body and TES.

Following the reviewer’s suggestion, we conducted the analysis and presented the data through a scatterplot, illustrating positive correlations between Pol II and RBM22 density at TSS, gene body and TES, as depicted in Fig. R4a. Considering the limited space in the figure, we have presented it in an alternative display (Fig. R4b) and included it as Fig. S2c in the revised manuscript.


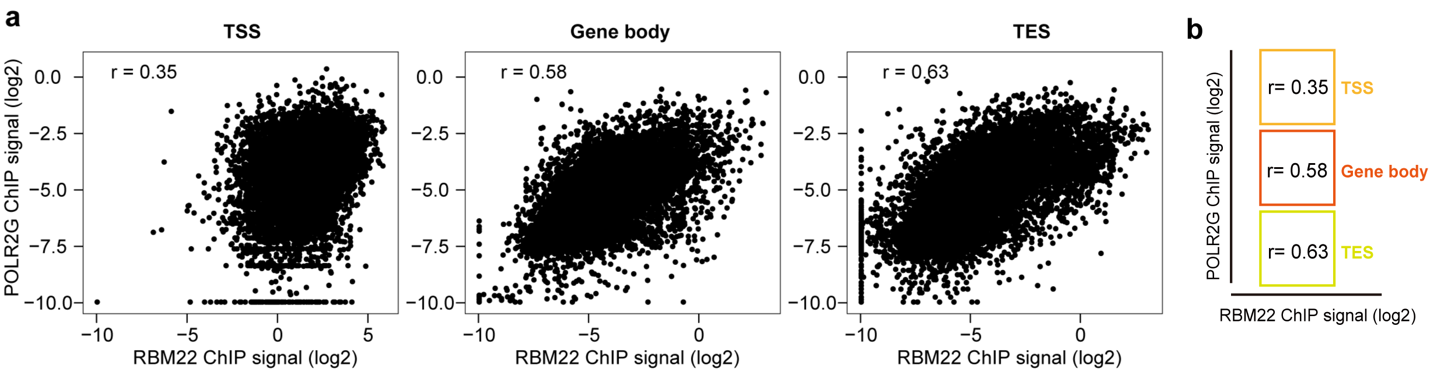


**Fig. R4.** Scatterplot showing the correlation between the POLR2G ChIP-seq signal and RBM22 ChIP-seq signal at promoter, gene body and TES.

Fig. 1e - (i) what about gene sets with different expression levels? It seems that genes with higher pausing are simply highly-expressed genes. (ii) How many genes in each group? Same concerns for 2g, 2h, 6c, 7d, etc......

We conducted a similar analysis as shown in Fig. 1e, revealing a weak reverse correlation between the degree of RBM22-regualted pause release and the expression levels of genes (Fig. R5a). This contracts with the positive correlation observed in Fig. 1e between the degree of RBM22-regualted pause release and Pol II pausing. Additional analysis showed no correlation between the gene expression levels and Pol II pausing, as illustrated in Fig. R5b. We have included these findings in the revised manuscript as a new supplementary figure, Fig. S2i.

Moreover, we have incorporated the gene numbers into the legends of Fig 2g, 2h, 6c, 7d, etc. in the revised manuscript.


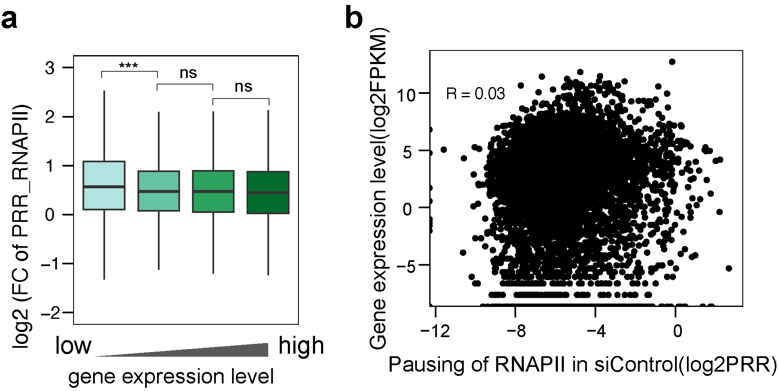


**Fig. R5.** Correlation between gene expression levels and RBM22-regualted pause release. (a) Boxplot showing the fold change (FC) of PRR (POLR2G ChIP-seq) at genes with different degree of gene expression in response to RBM22 depletion. The 9065 genes were equally divided into four groups based on the gene expression levels. The p value was determined using the wilcoxon test. (b) Scatterplot showing no correlation between the gene expression profiled by RNA-seq and pausing of RNAPII in control HepG2 cells.

Fig. S1m - Since the PRR ratio is an algorithm using an absolution position relative to TSS/TES (S1h), the difference between intron-less and intron containing genes in the genomic length may affect the result.

We apologize for any confusion caused by the inadequate description of our analysis. In Fig. S1m, we actually conducted a comparison of the PRR ratios of 70 intron-less genes and 70 intron-containing genes, both within the length range of 2 kb to 7 kb, ensuring a similar distribution, as depicted in Fig. R6. This analysis confirms that our results remain unaffected by variations in the gene length. To enhance transparency, we have included this information in the revised manuscript.


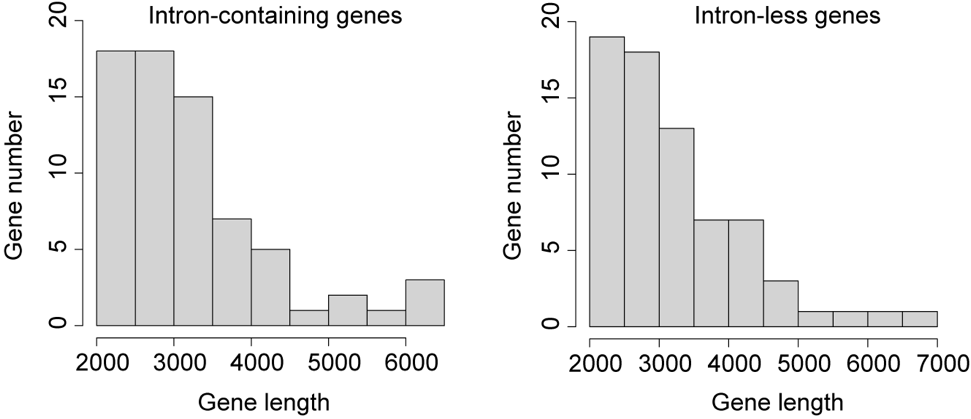


**Fig. R6.** Histogram showing the gene length distribution of intron-containing genes (left) and intron-less genes (right).

Fig. 1 and Fig. S1 - knockdown of components of U1/U2 snRNP can be a good control for ChIP experiments described in Fig.1 and S1.

We appreciate the Reviewer’s valuable suggestion. We investigated the impact of SF3B1, a core component of U2 snRNP, on Pol II pause release using Pol II ChIP-qPCR. As anticipated, knockdown of SF3B1 resulted in a decrease in Pol II pause release, as illustrated in Fig. R7. These findings agree with results obtained under splicing inhibition by Pla-B, a SF3B1-targeting agent [10]. Importantly, these findings further support the idea of a splicing-independent role of RBM22 in Pol II pause release. We have included these findings in the revised manuscript as new supplementary figures, Fig. S2k and l.


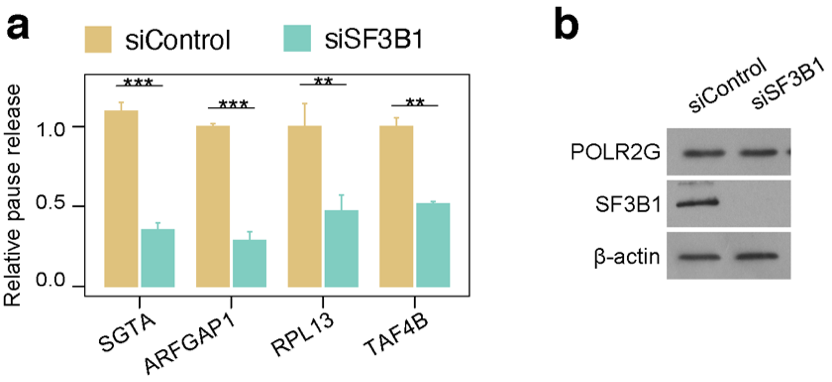


**Fig. R7.** ChIP-qPCR analysis of RNAPII pause release upon SF3B1 knockdown. (a) POLR2G ChIP-qPCR quantification of RNAPII pause release at four representative protein-coding genes in control and SF3B1 knockdown HepG2 cells. Graphs show the ratios of relative pause release. The p values are based on a two-tailed unpaired t test; *P < 0.05, **P < 0.01, ***P < 0.001. (b) Western blot showing the protein abundance of SF3B1 in wild-type cells or in SF3B1 depletion HepG2 cells.

Fig. 1i - It is interesting that uaRNA signals downstream of TSS were also increased. This may suggest that RBM22 functions by controlling chromatin conformation near TSS? The authors may include this in the discussion section.

We appreciate the reviewer's keen observation of the increased uaRNA signals downstream of the TSS upon RBM22 depletion. This interesting finding indeed raises the possibility that RBM22 may have a role in influencing chromatin conformation near the TSS. We have incorporated this valuable insight into the discussion section to explore potential connections between RBM22 function and chromatin dynamics. Thank you for pointing out this intriguing aspect of our data.

Fig. 2 - It is very odd that RBM22 arrests pol II near TSS, but unexpectedly promotes rapid pol II elongation. The authors should provide a hypothesis for the conflicting functions of RBM22 in transcription regulation.

In the original version, we had already proposed a hypothesis to account for the conflicting functions of RBM22 in transcription regulation within the discussion section. The observations that loss of RBM22 increases pause release and that RBM22 is required to maintain rapid elongation seem contradictory. Probably, enhanced pause release results in transcription-induced DNA supercoiling accumulation, which might impede RNAPII elongation and reduce its rate in turn [11, 12]. It is worth noting that a converse effect was observed on the negative elongation factor RECQL5, which promotes RNAPII pause release but decreases its elongation rate [13]. These observations may reflect a transcriptional balance between pause release and elongation rate.

Fig. 2d - the pol II signals were also reduced in the upstream region (Primer1, pausing site?) upon RBM22 knockdown. Why? It seems that RBM22 enhances the overall binding of pol II to the genome. But this assumption is not consistent with the conclusion from Fig. 1.

We appreciate this criticism. The reduced Pol II signals detected in the upstream region (Primer 1) may be attributed to the proximity of this region to the pausing site. In response, we redesigned Primer 1 at a more distal location from the pausing site and conducted qPCR again. As expected, this redesign resulted in a modest increase in Pol II signals in these regions. These results are consistent with the conclusion drawn from Fig. 1. We have incorporated these new data into the revised Fig. 2d in the revised manuscript, as shown in Fig. R8.


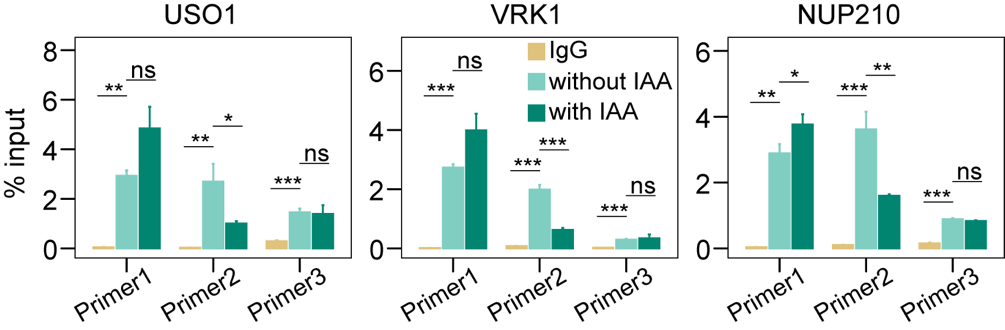


**Fig. R8.** POLR2A ChIP-qPCR showing the Pol II enrichment in different areas of example long genes in mAID-RBM22 cells with IAA or without IAA treatment. Values are mean ± SD (n=4). The p values are determined using the two-tailed unpaired t-test (*p<=0.05; **p<=0.01; ***p<=0.001; ns, not significant).

Fig. 2f - Please include the rationale of selecting the 415 genes in the analysis. What about other genes?

To compare Pol II elongation rate at individual gene level, we deliberately selected 415 genes with robust Pol II ChIP-seq signals at all three time points in both control and RBM22-depleted cells for calculation of Pol II elongation rate. The remaining genes did not meet these strict criteria. The rationale for selecting these specific 415 genes has been included in the revised manuscript.

Fig. 2g, h - Did the three gene sets belong to the 415 genes described in Fig. 2f?

Yes, they did.

Fig. S2h - is there a difference in the pol II elongation rate between intron-less and intron-containing genes?

Regrettably, we are unable to perform the suggested analysis. To accurately calculate Pol II elongation rate at the individual gene level, a minimal requirement is robust Pol II ChIP-seq signals at least at two time points (5 and 10 mins). Considering the estimated Pol II elongation rate of approximately 2 kb/min, as we presented in Fig. 2e-f, the minimal length of genes needed for this analysis is 20 kb. However, in the human genome, the longest intron-less gene (*PURB*) is approximately 9 kb long. Consequently, no intron-less genes meet the criteria for calculating elongation rate in our study.

Fig. 3 - please define active genes (e.g. line 266) and include the number of genes analyzed (e.g. 3b-d).

We apologize for the inaccurate description. To rectify this, we have made clarified by replacing “active genes” by “actively transcribed genes” and included the number of genes analyzed in Fig. 3b-d in the revised manuscript.

Fig. S3d - is there a difference in the level of DoGs between intron-less and intron-containing genes?

We quantified the levels of DoGs between intron-less and intron-containing genes, and our analysis revealed no significant difference was observed between them in both control and RBM22-depleted cells, as illustrated in Fig. R9. These results suggest that splicing may not be a factor influencing the production of DoGs.


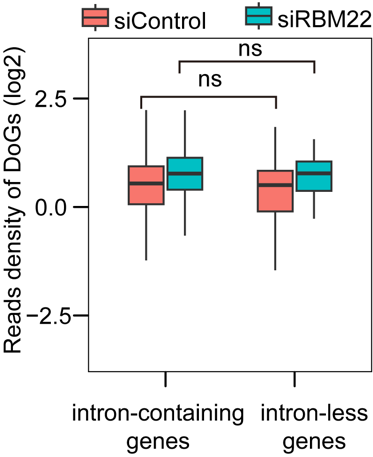


**Fig. R9.** Boxplot showing the GRO-seq signal at DoGs regions between intron-less genes and intron-containing genes in control and RBM22 knockdown HepG2 cells.

Fig. 3g and line 296-298 - sorry, I didn't get the point.

We appreciate the Reviewer’s diligence in catching the inaccuracy. In the revised manuscript, we have replaced the mentioned sentence with “We observed an increase in the number of DoG regions (>5 kb) from 939 in the control cells to 1416 in the RBM22-depleted cells, with 256 regions lost and 733 new regions gained (Fig. 3g).”

Fig. S3g and line 303-305 - the expression of DoG downstream genes was largely unaffected by RBM22 knockdown, which is inconsistent with the prior conclusion that RBM22 plays a general role in transcription regulation. RBM22-mediated pol II pausing does not occur at these downstream genes??? Why?

We apologize for any confusion caused by our analysis. Please note that the analysis in original Fig. S3g aimed to access the impact of DoG transcripts on downstream gene expression particularly using RNA-seq data instead of GRO-seq data, because GRO-seq signals at downstream genes can be influenced by DoG transcripts. Moreover, it’s crucial to emphasize that the gene expression levels obtained from RNA-seq data cannot accurately represent the level of transcription.

Upon re-analysis of the data, we observed that approximately 60% of DoG downstream genes remain consistently unexpressed before and after RBM22 knockdown, as illustrated in Fig. R10a. Additionally, more than half of DoG downstream genes showing expression were unaffected by RBM22 knockdown (Fig. R10a). Further analysis of the Pol II PRR of DoG downstream genes with expression, whether on the same or opposite strands, revealed an increase in Pol II PRR upon RBM22 knockdown (Fig. R10b), in agreement with our prior conclusion that RBM22 facilitates Pol II pausing. The Fig. R10a has been incorporated into new Fig. S4f in the revised manuscript.


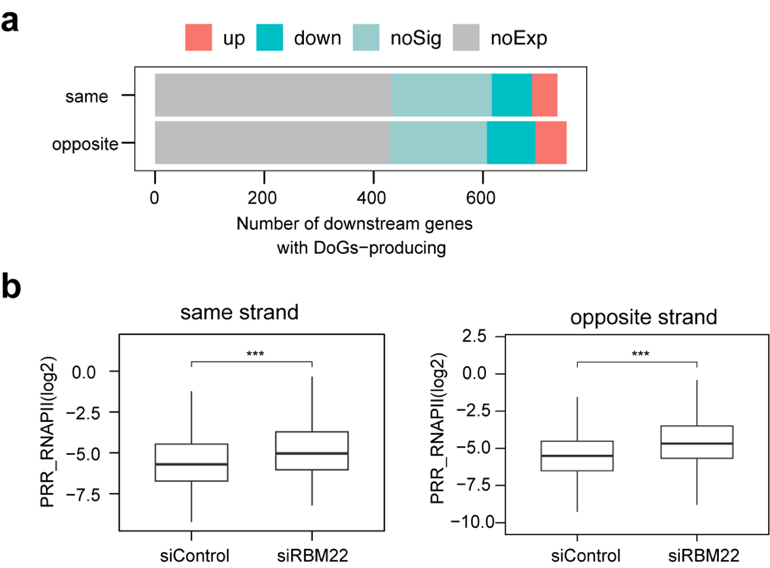


**Fig. R10.** Gene expression and RNAPII pause release for downstream genes of DoGs-producing genes. (a) Barplots showing the number of unexpressed genes (noExp), genes with no significant change (noSig) and genes showing significant upregulation or downregulation among downstream genes associated with DoGs-producing genes in both directions after RBM22 knockdown. (b) Boxplots showing the PRR of downstream genes associated with DoGs-producing genes after RBM22 knockdown in same (left) or opposite (right) strand in control or RBM22 knockdown HepG2 cells. The p value was determined using the wilcoxon test.

Fig. 4 - Again, please include the rationale of testing snoRNA and snRNA.

The testing snoRNA and snRNA in Fig. 4 is justified by the evident occupancy of RBM22 on these genomic regions. Our original version already provided the rationale for the analyses in Fig. 4.

Fig. 4a, b - Pol II density around TSS was similarly increased upon RBM22 knockdown. This suggests that RBM22 does not function in paused pol II release and that RBM22 simply enhances the overall binding of pol II to the locus.

We appreciate the Reviewer's attention to the observed increase in Pol II density around TSS of snoRNA and snRNA genes in Fig. 4a, b upon RBM22 knockdown. The Pol II density around TSS indeed appears unusual and upon careful examination, we identified that the strong but abnormal signals at *RNU2-64P*, characterized by signals only at TSS without signals in the gene body, contributed to this anomaly. By excluding this gene from the analysis, we observed an increase in Pol II density across the remaining snoRNA and snRNA genes (Fig. R11). We have made revisions to the relevant figures, specifically in Fig. 4, in the revised manuscript.


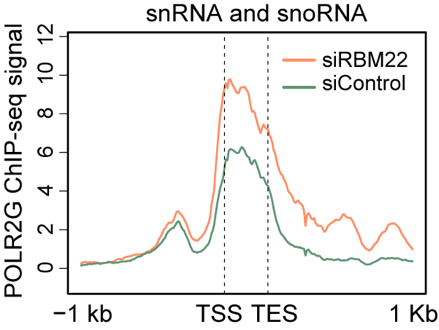


**Fig. R11.** Metagene analysis showing the change in POLR2G ChIP-seq signal at independently transcribed snoRNA and snRNA genes (N=25) upon RBM22 knockdown.

In response to the Reviewer’s suggestion, our interpretation is that the increased Pol II density does not imply a simple enhancement of overall Pol II binding to these loci. Instead, our data suggest a nuanced role for RBM22 in regulating transcription of short non-coding genes. Given that all transcription processes take place in a very short region at this special category of short non-coding genes, the increased Pol II density around TSS may be indicative of a complex interplay involving the release of paused Pol II and potential downstream processes or effects.

Fig. 4c and line 318-322 - I didn't observe a large increased readthrough signal right after TES (100 bp from TES).

We agree. Accordingly, we have replaced “large” with “significant” in the revised manuscript.

Fig. S4k - Unlike regular DoGs, sRDoGs are unstable transcripts that might be subjected to quick degradation by nuclear quality control. The authors may consider providing a discussion about this phenotype.

Following the Reviewer’s suggestion, we have provided a discussion about this phenotype in the revised manuscript.

Fig. 5b-f - Does the interaction depend on transcription? The author may consider performing the indicated coIP experiments (at least for the important one) using Actinomycin D-treated cells.

To address this question, we performed the coIP experiments upon Actinomycin D treatment. We observed that Actinomycin D treatment leads to an increase in the levels of Ser2P Pol II (Fig. R12), consistent with previous observation [14, 15], however, the relative level of RBM22 associated with Ser2P Pol II remains unchanged (Fig. R12). These results suggest a transcription-independent interaction between RBM22 and Pol II. These findings have been included as a new Fig. 5i in the revised manuscript.


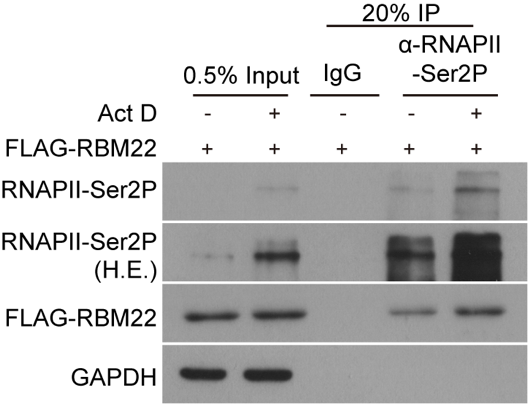


**Fig. R12.** Effect of Actinomycin D treatment on the interaction between RBM22 and RNAPII in HepG2 cells.

Fig. 5g and S5c - The authors concluded that CDK7 and CDK9 (but not CDK12) are required for the RBM22-polII interaction. But knockdown of CDK12 obviously upregulated the level of CDK7 as shown in S5c, which may enhance the interaction. These data somehow are not consistent. Why?

We appreciate the Reviewer’s attention to Fig S5c; however, there might be a misunderstanding. Contrary to the concern raised, the level of CDK7 remained unchanged upon the knockdown of CDK12, as accurately depicted in the figure.

Fig. 5i, j - It is a great experiment that demonstrates the connection between RBM22 and the inhibitory 7SK complex. Since RBM22 is a RNA-binding protein, it is also important to determine whether RBM22 functions through its RNA-binding activity. The authors may consider (a.) checking the interaction between 7SK RNA and RBM22; (b.) performing the indicated coIP experiments using 7SK-KD or RNase A-treated samples.

In the original version, we have already accessed the published RBM22 eCLIP data in HepG2 cells [16] and confirmed the interaction between RBM22 and 7SK noncoding RNA, as illustrated in Fig. S6e.

We apologize for the inadequate description of the co-IP experiment. In fact, all the co-IP experiments were conducted using benzonase-treated cell lysates, consistent with the approach used in the IP-MS experiment. This step was taken to eliminate any nucleic acid-dependent interactions. Consequently, we can rule out the possibility that RBM22 interacts with the inhibitory 7SK complex proteins through its RNA-binding activity. Importantly, we have included this crucial information in the revised method section.

Fig. 6a, b - It is odd that the binding of inhibitory and active components of 7SK-P-TEFb to the active loci exhibited a SIMILAR trend to each other upon RBM22 knockdown, which is also inconsistent with 6d. Actually, 6b showed a slight increase in HEXIM1 density downstream of TSS. Why?

The clear HEXIM1 and CDK9 ChIP-seq signals observed at TSS in control cells (Fig. 6a, b, e) suggest a genome-wide inhibition of P-TEFb within the 7SK-P-TEFb complex at gene promoters. Upon RBM22 knockdown, we indeed observed a similar decrease in the binding of both inhibitory (HEXIM1) and active (CDK9) components of 7SK-P-TEFb to the TSS. However, their functional consequences differ. The decrease in HEXIM1 binding at TSS (Fig. 6a-b), coupled with its disassociation with CDK9 (Fig. 6d), suggests a liberation of CDK9 from the inhibitory 7SK-P-TEFb complex at TSS. This leads to the translocation of CDK9 from promoter into gene body, resulting in a decreased TSS binding.

We appreciate the Reviewer’s attention to Fig. 6b; however, there might be a misunderstanding. Please note that the heatmap of subtract in Fig. 6b presented the subtracted values of siRBM22 minus siControl, providing a clear visualization of the decrease in HEXIM1 density at TSS.

Fig. 6f - Please perform a same analysis using the HEXIM1 and SPT5 ChIP data as controls.

We appreciate the Reviewer’s suggestion. The analysis in Fig. 6f aims to compare the relative CDK9 accumulation before and after the +1 nucleosome. We performed a same analysis using the HEXIM1 and SPT5 ChIP data. Unlike CDK9 accumulation (Fig. 6f), HEXIM1 showed a decrease in accumulation before the +1 nucleosome, with only a subtle increase after the +1 nucleosome (Fig. R13a), consistent with its disassociation from the promoter (Fig. 6b). To enhance clarity, we have incorporated this control as new Fig. S7d in the revised manuscript.

Furthermore, despite the global decrease in SPT5 ChIP signals at genes (Fig. 7c-d), the relative accumulation of remaining SPT5 appeared to decrease before the +1 nucleosome while increase after the +1 nucleosome in response to RBM22 knockdown, as seen in Fig. R13b. This observation aligns with the established role of SPT5 as a Pol II-associated elongation factor [17, 18] and supports an increase in Pol II pause release.


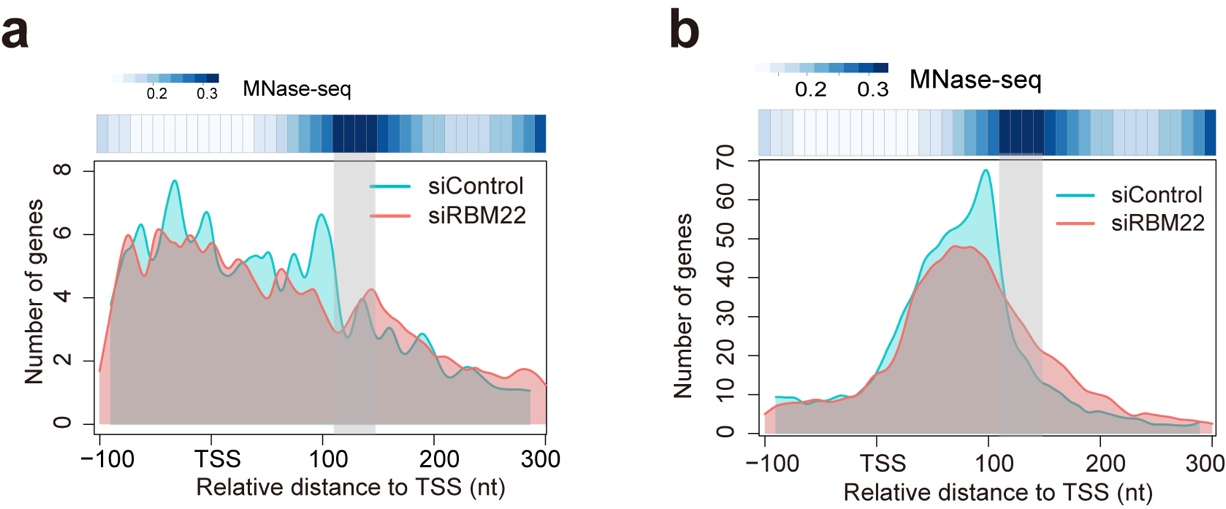


**Fig. R13.** Distance distribution of HEXIM1 and SPT5 ChIP-seq peak summit relative to TSS and +1nucleosome dyads. (a) The +1nucleosome position is determined by MNase-seq. The y axis represents the gene number of HEXIM1 accumulation at relative positions from summit to TSS in control and RBM22 knockdown cells. (b) The y axis represents the gene number of SPT5 accumulation at relative positions from summit to TSS in control and RBM22 knockdown cells.

Fig. 7a - Please see the comments for 5b-f and 5i, j.

Unlike the co-IP experiments performed in Fig. 5b-f and 5i, j, we conducted a pull-down assay *in vitro* in Fig. 7a. For this assay, we separately purified RBM22 and SPT5 proteins to investigate whether RBM22 directly interacts with SPT5. Our results unmistakably demonstrate a direct interaction, which is both RNA-independent and transcription-independent.

Please carefully check the grammar errors.

We appreciate the Reviewer's feedback. We have carefully reviewed the manuscript and addressed the grammar errors. Please let us know if there are any specific instances or areas where further improvements are needed.

In response to the constructive feedback provided by the Reviewer, we have carefully addressed each comment and concern, aiming to enhance the clarity, validity and completeness of our manuscript.

**Reviewer #3:**

The report by Du et al documents an activity by an established splicing factor and an RNA-binding protein (RBP), RBM22, in regulating multiple stages of gene transcription by RNA polymerase II (Pol II) in human cells. The authors provide several lines of evidence that the transcription-regulatory activity of RBM22, which, as reported, plays critical roles in the control of transcriptional pause release, elongation, and termination, is independent of its role in splicing. They also identify RBM22 as the first non-canonical component of the 7SK complex that promotes its inhibitory activity, thereby antagonizing Pol II pause release.

This is a well-designed and well-executed study with important implications for the fields of transcription and splicing. The study also makes an important new case for a general RBP in regulating gene transcription and, potentially, coordination of transcription with post-transcriptional processing. The main strength is discovery of new activity via genome-wide approaches and the key conclusions are sufficiently supported biochemically.

I congratulate the authors for their findings and have no serious concerns that would preclude publication of this study. However, I provide some recommendations, grouped below into major and minor comments, that might help clarify several points and should be fairly easy to address. I also recommend that the manuscript be edited for grammatical errors.

We sincerely appreciate the Reviewer’s positive comments and constructive suggestions. Accordingly, we have obtained all needed new data to address Reviewer’s concerns, as detailed below.

Major comments:

1) A critical finding of this work is that a splicing factor regulates transcription independent of its role in splicing. Since RBM22 is not the first such case, can the authors speculate on why splicing factors might be particularly well suited to 'moonlight' as transcriptional regulators? Some discussion of this would give the motivation for this work and its conclusions more context.

We appreciate the Reviewer's recognition of the critical finding that splicing factors, including RBM22, can regulate transcription independent of their canonical role in splicing. The phenomenon of splicing factors 'moonlighting' as transcriptional regulators is indeed intriguing and not unique to RBM22. While this study focuses on RBM22, we acknowledge that exploring the broader context and potential reasons behind this phenomenon is valuable.

Splicing factors, by virtue of their involvement in the intricate process of mRNA maturation, may have evolved multifaceted roles to coordinate and fine-tune gene expression. One speculation is that splicing factors, being intimately connected with nascent RNA, could influence various steps of transcription, from initiation to elongation and termination. Additionally, they may interact with components of the transcription machinery, forming dynamic regulatory networks.

This 'moonlighting' phenomenon raises intriguing questions about the evolution and functional versatility of splicing factors. Further investigations into the specific molecular mechanisms and evolutionary pressures driving splicing factors to take on additional roles in transcription will undoubtedly contribute to a deeper understanding of cellular regulatory networks. We have included a discussion of these aspects in the revised manuscript to provide more context and motivation for our work and its conclusions.

2) RBM22 presumably travels with Pol II as it regulates its activity. Since much of the pre-mRNA splicing occurs co-transcriptionally, one wonders if the same Pol II-associated molecule of RBM22 also facilitates splicing (e.g., by transiently becoming part of the spliceosome). While experiments to resolve this in detail may be challenging, it would nevertheless be important to understand whether the bulk of RBM22 forms just one large protein complex with Pol II and components of the spliceosome, or it forms separate complexes, one with Pol II and associated factors and one with the spliceosome. A relatively simple experiment to address this would be density (e.g. glycerol) gradient-based fractionation of cell lysates followed by western analysis of protein subunits that are specific to each machinery. Such analysis might also find RBM22-7SK complex as a separate species. Since this could provide support for - and clarify - the key arguments about coordination of different Pol II activities and splicing, I recommend that the authors give this a try. All the necessary antibodies are at hand.

We appreciate the Reviewer's insightful suggestion to investigate the potential association of RBM22 with both Pol II and the spliceosome, which could shed light on the coordination of different Pol II activities and splicing. To address this question, we performed the glycerol gradient fractionation analysis of RBM22 complexes in cell lysates. Our results revealed that approximately 40% of RBM22 is associated with Pol II, forming a large protein complex with spliceosome (Fig. R14, Fractions 11-19). The remaining fraction of RBM22 forms a smaller complex with the free 7SK-P-TEFb complex under our sedimentation conditions (Fig. R14, Fractions 1-5). These findings suggest that RBM22 coordinates different Pol II activities and splicing through association with distinct complex. We have incorporated these new findings, as a new Fig. 5l, into the revised manuscript.


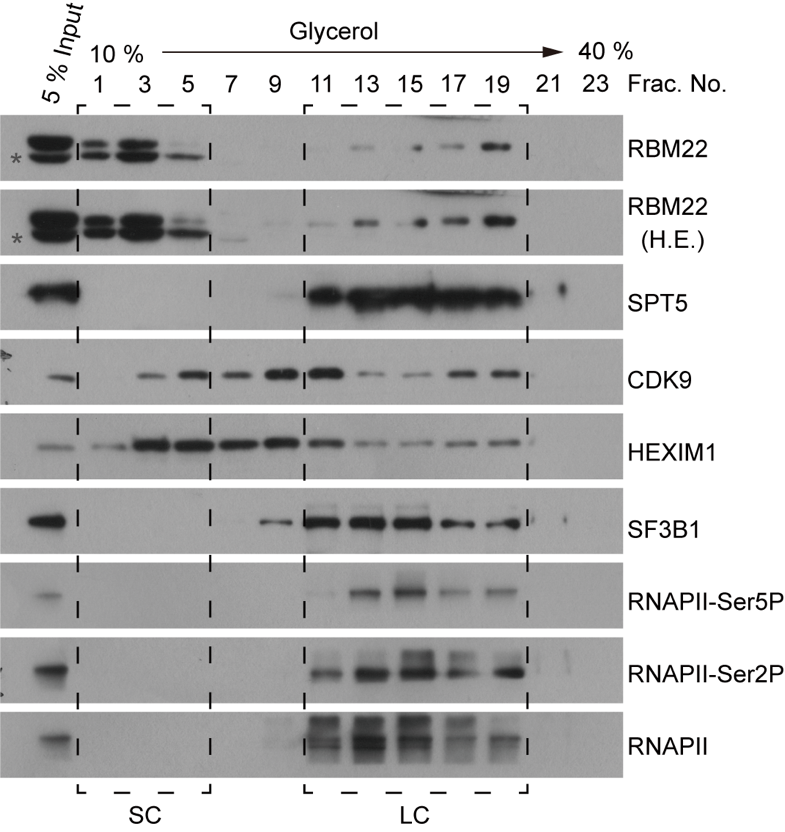


**Fig. R14.** RBM22 co-fractionation with Pol II and spliceosome and identification of separate RBM22-7SK Complex. HepG2 cell lysate was analyzed by glycerol gradient sedimentation. Collected fractions were detected by Western blotting. The dashed box highlights a large complex (LC) and a small complex (SC) associated RBM22. Asterisk indicates non-specific signal.

3) Approximately 30%-40% of metazoan genes are regulated via promoter-proximal Pol II pausing, particularly genes participating in signal-responsive pathways (see e.g. Adelman & Lis, Nat Rev Genet 2012). Pol II pausing at only a fraction of all paused genes is regulated by the inhibitory 7SK complex. However, the authors report on the role of RBM22 in controlling Pol II pause release "at most gene promoters". I suggest that this be better explained, given that most genes are likely not regulated by pausing. Are the genes known to be regulated by the 7SK complex (e.g. study in ESCs by Castelo-Branco et al, Genome Biol 2013) particularly strongly regulated by depletion of RBM22? Is regulation by RBM22 via 7SK likely to be mandatory for the control of pausing? Also, are genes whose pausing is strongly regulated by RBM22 enriched in any specific gene ontology (GO) categories?

We greatly appreciate the Reviewer’s expertise and the critical review regarding the proportion of metazoan genes regulated by Pol II pausing. We apologize for any confusion caused by our inaccurate description. In response to this concern, we have revised the manuscript to replace “at most gene promoters” with “at many gene promoters” for improved accuracy. Notably, 4507 genes displayed a ≥ 1.5 fold change in Pol II pause release ratio (PRR), with 3960 showing an increase and 547 showing a decrease. This indicates that 22% of genes are subjected to regulation by RBM22-mediated pausing, a proportion well within the normal range.

Furthermore, the Reviewer raised three interesting and important questions. In response, we have performed additional analyses to address the queries.

1. Are the genes known to be regulated by the 7SK complex (e.g. study in ESCs by Castelo-Branco et al, Genome Biol 2013) particularly strongly regulated by depletion of RBM22?

In our comparative analysis, we found that, in contrast to non-regulated genes, those known to be regulated by the 7SK complex in mESCs [19] do not appear to exhibit strong regulation upon RBM22 depletion, as illustrated in Fig. R15. This observation suggests a potential cell type-specific response [20-22], emphasizing the nuanced nature of RBM22's impact on different gene sets.


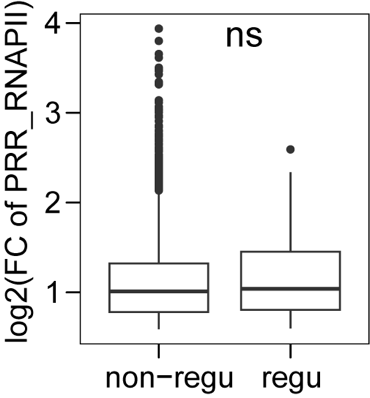


**Fig. R15.** Boxplot showing the fold change (FC) of PRR (POLR2G ChIP-seq) for the genes regulated by the 7SK complex (regu) and non-regulated genes (non-regu) in mESCs in response to RBM22 depletion. The p value was determined using the wilcoxon test.

1. Is regulation by RBM22 via 7SK likely to be mandatory for the control of pausing?

We appreciate the insightful question raised by the Reviewer regarding the potential mandatory role of RBM22 via 7SK in controlling pausing. Our study indicates that RBM22 has a broad impact on Pol II pause release, but the extent to which it is mandatory may vary across different gene contexts. While RBM22 depletion affects pausing at many gene promoters, we recognize that additional factors and mechanisms, such as SPT5 as studied in Fig. 7, are likely involved in the regulation of pausing.

Our future investigations will aim to delineate the specific scenarios where the regulatory function of RBM22, potentially via the 7SK complex, is indispensable for controlling pausing.

1. Are genes whose pausing is strongly regulated by RBM22 enriched in any specific gene ontology (GO) categories?

We performed a Gene Ontology (GO) analysis for genes whose pausing is strongly regulated by RBM22. The analysis revealed enrichment in various biological processes, including cellular component disassembly, proteasome-mediated ubiquitin-dependent protein catabolic process, vesicle organization, regulation of autophagy, macroautophagy, Golgi vesicle transport, and more. These findings are illustrated in Fig. R16. These results have now been included as a new Fig. S2h in the revised manuscript.


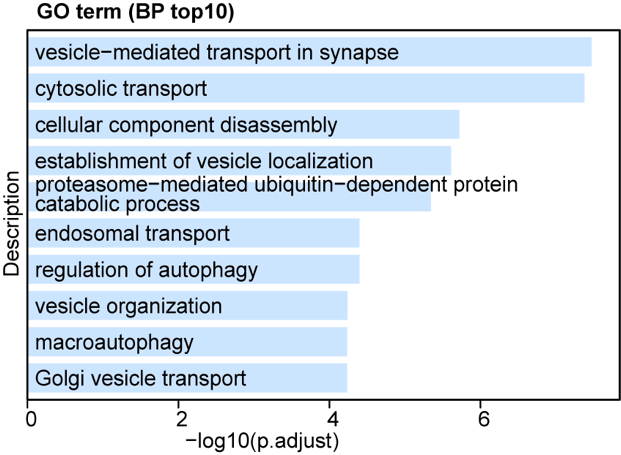


**Fig. R16.** Barplot showing the gene ontology for the ten most significantly enriched biological processes for the genes strongly regulated by RBM22.

4) I suggest that the authors demonstrate the specificity of their RBM22 ChIP signal using either siRBM22 or inducible degradation of mAID-RBM22. A ChIP-qPCR experiment should suffice.

We appreciate the Reviewer's suggestion. We performed RBM22 ChIP-qPCR using mAID-RBM22 cells. The results demonstrated a significant decrease in ChIP signals at different regions of *FUS*, *SGTA* and *U3* genes upon acute depletion of RBM22 (Fig. R17). These findings confirm the specificity of RBM22’s interaction with these genomic regions. We have incorporated these results into the new Fig. S2j in the revised manuscript.


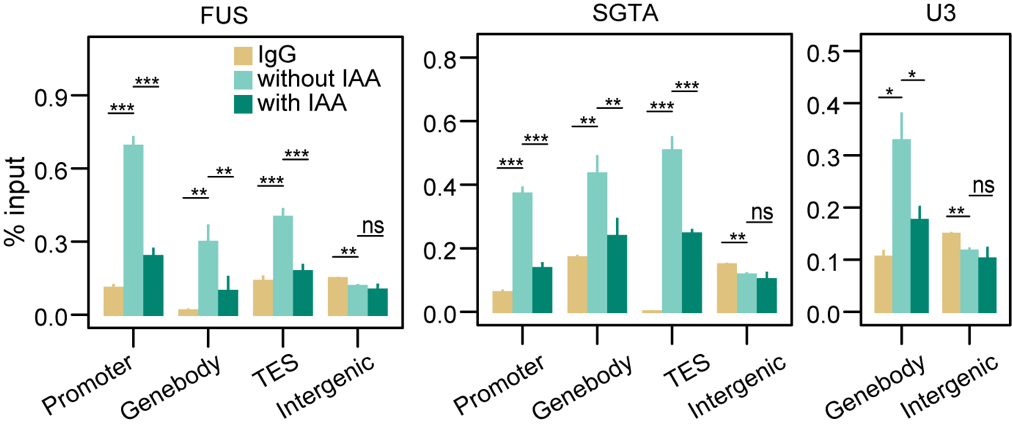


**Fig. R17.** ChIP-qPCR analysis of the RBM22 binding level at the promoter, gene body, TES and intergenic region of the protein-coding genes or at the gene body and intergenic region of the snoRNA gene. The p values are based on a two-tailed unpaired t test; (*P < 0.05; **P < 0.01; ***P < 0.001; ns, not significant).

5) On the positive correlation between elongation velocity and RBM22 occupancy (Fig. 2g): can the authors speculate on what recruits RBM22 to genes with higher elongation velocity? I recommend that the same bins of genes (low/medium/high elongation rates) be analyzed for intron density, Pol II S5P vs S2P, and SPT5 occupancy.

We appreciate the Reviewer's insightful question regarding the positive correlation between elongation velocity and RBM22 occupancy as depicted in Fig. 2g. While the precise mechanisms that recruit RBM22 to genes with higher elongation velocity require further investigation, we have considered potential factors influencing this association.

In response to the Reviewer's recommendation, we analyzed the same bins of genes categorized by low, medium, and high elongation rates for intron density, Pol II S5P vs S2P ratio, and SPT5 occupancy. Our analyses revealed a positive correlation between elongation rate and SPT5 occupancy (Fig. R18a), a positive correlation between elongation rate and the ratio of Pol II S5P to S2P (Fig. R18b), and a weak positive correlation between elongation rate and intron density (Fig. R18c). In addition to RBM22 occupancy, these additional findings provide valuable insights into the intricate relationships between elongation velocity and other key regulatory factors. We have incorporated these results into the revised manuscript for a more comprehensive understanding.


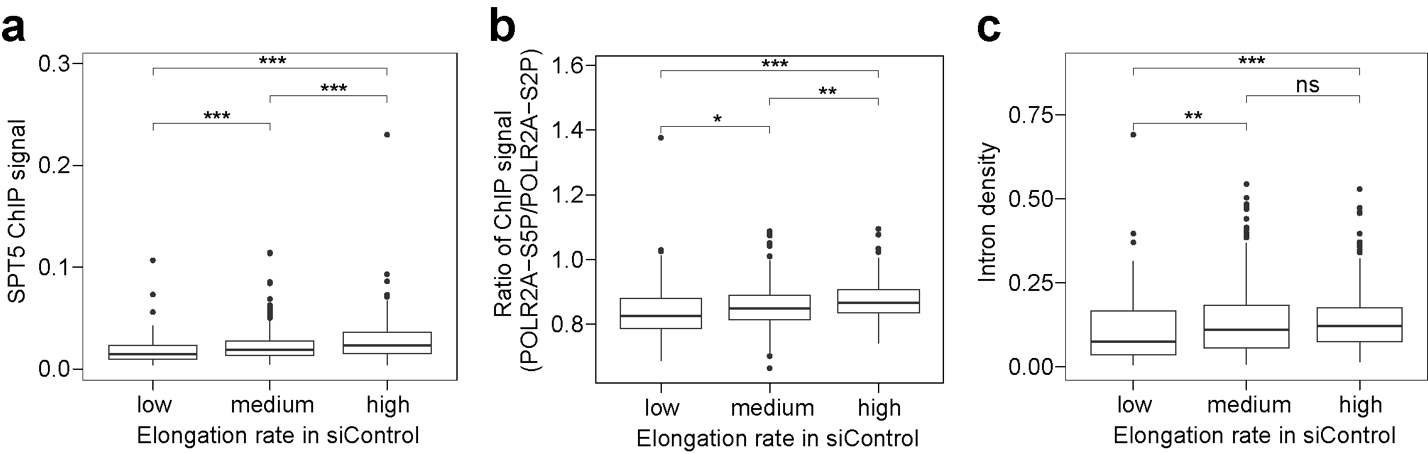


**Fig. R18.** Relationship between elongation rates and intron density, Pol II S5P vs S2P ratio, and SPT5 occupancy. (a-c) Boxplot analysis of the SPT5 ChIP-seq signals (a) and the ratio of Pol II S5P to S2P (b) and intron density (c) upon RBM22 knockdown for the genes with different elongation rate in control cells. The genes (N=597) were divided into three groups based on elongation rate in control cells: low (rate < 1.87kb/min), medium (1.87kb/min < rate < 2.36kb/min) and high (rate > 2.36kb/min). The p values are determined using the two-tailed unpaired t-test (*p<=0.05; **p<=0.01; ***p<=0.001).

6) How do the authors explain the observation that Pol II pause release is much more efficient upon RBM22 knockdown by siRNA (which is good but incomplete; Fig. S1f) than after acute loss of RBM22 (which is essentially complete; compare Figs. S1k and 1g)?

We appreciate the Reviewer's observation and concern regarding the discrepancy in Pol II pause release efficiency between RBM22 knockdown by siRNA (Fig. S1k) and acute loss of RBM22 (Fig. 1g). To make it clear, we carefully repeated the ChIP-qPCR experiment in Fig. S1k and obtained similar changes (Fig. R19) with the results upon acute loss of RBM22 in Fig. 1g. These findings were also consistent with the PRR changes obtained from Pol II ChIP-seq data in Fig. S1i. We have included these new data as a new Fig. S2g in the revised manuscript.


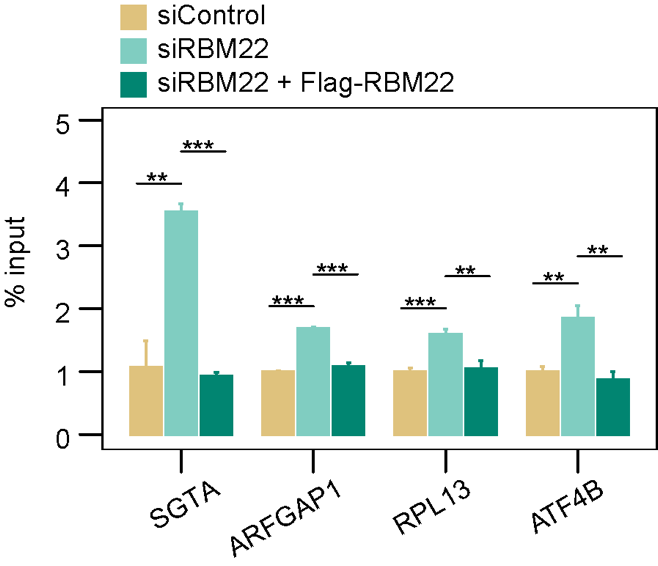


**Fig. R19.** POLR2G ChIP-qPCR quantification of RNAPII pause release at four representative protein-coding genes in control, RBM22 knockdown and re-expression of siRNA-resistant wild-type RBM22 after depletion of RBM22 HepG2 cells. Graphs show the ratios of relative pause release, normalized to control. The p values are based on a two-tailed unpaired t test; *P < 0.05, **P < 0.01, ***P < 0.001.

7) Readthrough index (R1) uses as the numerator GRO-seq read density in the region from TES to the TSS of a downstream gene (Fig. S3b). I find this problematic since this distance may vary, depending on the location of a gene, by several orders of magnitude. Instead, a fixed distance 3' of TES should be defined and used to calculate R1.

We appreciate the Reviewer's attention to the methodology used for calculating the readthrough index (RI) in our study. The choice of GRO-seq read density in the region from TES to the TSS of a downstream gene aims to capture the signals of transcriptional readthrough as much as possible. Additionally, this flexible criterion also allows us to calculate RI for more genes than using a fix distance 3’ of TES.

Furthermore, in fact, we have already used length-normalized GRO-seq read density to calculate RI (Fig. S3b), thus ruling out the potential influence by distance variability from TES to the TSS of a downstream gene. This clarification has been incorporated into the revised manuscript.

8) It is not immediately obvious why the authors liken the readthrough seen upon loss of RBM22 specifically to stress-induced transcriptional readthrough observed by Steitz and colleagues (lines 267 and 301/302)? With numerous other reported cases of induced transcriptional readthrough, is there anything that makes osmotic stress-induced readthrough particularly "reminiscent" of the current results? A comparison is made between the functions of "DoG-producing" genes in this and the Steitz's study, and a difference is found, but what is the relevance of this finding? What does the enrichment of genes with roles in viral gene expression imply? This should be either clarified or removed.

We agree. We have removed these contents in the revised manuscript.

9) In what sense are readthrough transcripts downstream of sno/snRNA genes of a "new type" (chapter starting with line 341)? The authors find that these transcripts are not polyadenylated, but are DoG transcripts of protein-coding genes polyadenylated?

We appreciate the Reviewer's question regarding the characterization of readthrough transcripts downstream of sno/snRNA genes as a 'new type.' In our study, we refer to them as a 'new type' because they initiate from sno/snRNA genes, distinguishing them from previously characterized readthrough transcripts that typically initiate from protein-coding genes. Furthermore, these sno/snRNA-associated readthrough transcripts were rarely observed in previous studies. This term is used to emphasize the novelty and specificity of these transcripts in the context of our investigation. We have clarified this point in the revised manuscript to provide a more detailed explanation.

In our analysis of polyadenylation of DoG transcripts of protein-coding genes, we examined our c3’-seq data and identified polyadenylation signals in more than 25% of DoG transcripts (Fig. R20). Notably, this phenomenon is in agreement with observations from a previous study [23].


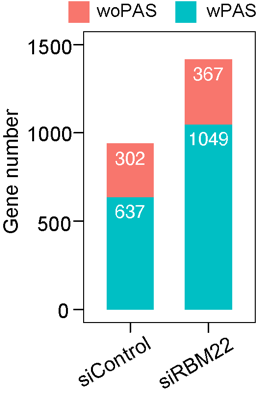


**Fig. R20.** Number of DoG transcripts polyadenylated (wPAS) or not polyadenylated (woPAS) in control and RBM22 knockdown cells.

10) It is stated that Ser5 and Ser2 phosphorylation was "eliminated" by siRNA-mediated KD of CDK7, CDK9, and CDK12 (Fig. 5; lines 389-391), but the KD efficiency of CDK7 and CDK9 is quite weak, maybe 60% (Fig. S5c), unlikely to suffice for a very significant reduction in phosphorylation. It would help if the authors showed the actual remaining levels of phosphorylation, as they do (and that is convincing) for the use of inhibitors (Fig. S5d). In addition, text should clarify what KD of each CDK7, CDK9, and CDK12 is expected to do and why a very good KD of CDK12 shows no effect on Pol II - RBM22 association.

We appreciate the Reviewer’s careful scrutiny of our siRNA-mediated knockdown efficiency (KD) for CDK7, CDK9 and CDK12 and the subsequent impact on phosphorylation events. We acknowledge the point raised about the KD efficiency of CDK7 and CDK9 being around 60% (Fig. S5c). It’s important to note that achieving a higher KD efficiency for CDK7 and CDK9 without compromising cell viability is challenging, at least in this case.

To provide data of the remaining levels of Pol II phosphorylation, we performed western blotting experiment. As expected, CDK9 knockdown reduced Ser2P RNAPII (Fig. R21), consistent with the results obtained under similar KD efficiency in the previous report [24]. Additionally, CDK7 knockdown decreased both Ser5P and Ser2P RNAPII, in agreement with previous results obtained upon CDK7 inhibitor, THZ1 [25-28]. Together, these results suggest that the levels of Pol II phosphorylation may be sensitive to the downregulation of these kinases.

Notably, the downregulation of both Ser2P and Ser5P Pol II upon CDK7 KD did not result in a stronger decrease in the interaction between Pol II and RBM22 in comparison with CDK9 KD (Fig. 5g). It is possible that CDK9, as an interacting protein of RBM22 (Fig. 5a, c and i) and Pol II [8, 29], may also have a role in enhancing their interaction through protein-protein interaction. This possibility remains to be further studied.

Furthermore, we have revised the text to better clarify the expected effects of KD for each CDK. Additionally, CDK12 KD did not resulted in changes in the levels of Pol II phosphorylation (Fig. R21), possibly due to the presence of its functionally redundant kinase, CDK13, in cells [30-32]. These findings were consistent with the results in previous report [33]. As a result, we observed no significant effect on Pol II-RBM22 association (Fig. 5g). We appreciate the opportunity to improve the clarity and completeness of our manuscript. This data has been incorporated as a new Fig. S6c in the revised manuscript.


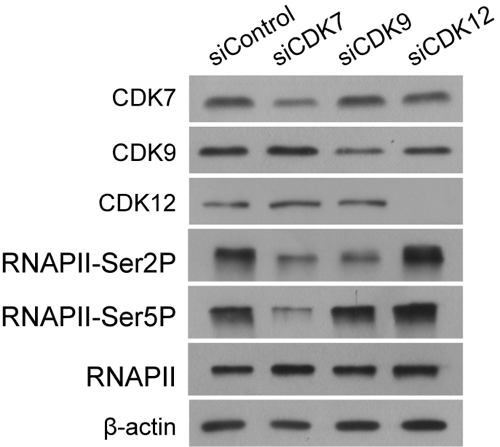


**Fig. R21.** The impact of CDK7/CDK9/CDK12 knockdown on Pol II phosphorylation. Western blot results showing the knockdown efficiency of the siRNAs against CDK7, CDK9, CDK12 and the effect of CDK7, CDK9, CDK12 knockdown on the levels of Pol II phosphorylation. β-actin serves as loading control.

Minor comments:

- The way that NGS data were normalized should be included in the methods section.

We have included the normalization approach of NGS data in the revised methods section.

- Please cite figure panels in their alphabetical order. Cite S1d before S1e (line 128), cite Figs. 6c and 6d before 6e (line 417).

We have fixed it accordingly.

- All co-IP/western experiments should state what % of total material was loaded in "input" and what % was loaded in "IP" lanes. This is important to gauge the efficiency of co-IP.

We have fixed it accordingly.

- Were the co-IP/western experiments in Figs. 5i and 5j done in the presence of RNase? Do the authors expect that the association of RBM22 with the 7SK protein components relies on RBM22 - 7SK ncRNA binding?

All the co-IP experiments, including those in Figs. 5i and 5j, were conducted using benzonase-treated cell lysates, consistent with the approach used in the IP-MS experiment. This step was taken to eliminate any nucleic acid-dependent interactions. Consequently, we can rule out the possibility that RBM22 interacts with the inhibitory 7SK complex proteins through its binding to 7SK ncRNA. We have included this crucial information in the revised manuscript.

- Line 161, correct Fig 1Sl to Fig S1l

We have fixed it accordingly.

- Line 226: the reference, I believe, should be to Fig. 2d, not 3d.

We have fixed it accordingly.

- Line 233 and Fig. 2f: which 415 genes were analyzed here?

To compare Pol II elongation rate at individual gene level, we deliberately selected 415 genes with robust Pol II ChIP-seq signals at all three time points in both control and RBM22-depleted cells for calculation of Pol II elongation rate. The remaining genes did not meet these criteria. The rationale for selecting these specific 415 genes has been included in the revised manuscript.

- Line 298: increased "intensity" of DoG transcripts should be replaced with "expression".

We have replaced “intensity” with “expression” accordingly.

- In Fig. S5b, what does log2FC(+Dox/-Dox) pertain to? Fold change (FC) of what? This should be explained in the figure legend.

We have made an explanation in the revised figure legend accordingly.

- Line 417: "Activating" would seem better than "active".

We have replaced “active” with “Activating” accordingly.

- Line 435: Cite literature documenting CDK9-HEXIM1 interaction by co-IP. 

We have cited the relevant literatures accordingly.

- Fig. 6f, y-axis: are these numbers of genes in thousands? Please fix. 

We have fixed it accordingly.

- Fig. 6h: what are the yellow lines for? It should not be necessary to have two legends.

We have fixed it accordingly.

- Lines 470-472: the conclusion should mention RBM22.

We have included RBM22 in the conclusion.

Through these revisions, we believe the manuscript is significantly improved in terms of clarity, robustness, and completeness. We appreciate the insightful feedback from the reviewers, which has contributed to the overall enhancement of the manuscript.

**Reference**

1. Sheridan RM, Fong N, D’Alessandro A, Bentley DL: Widespread Backtracking by RNA Pol II Is a Major Effector of Gene Activation, 5′ Pause Release, Termination, and Transcription Elongation Rate. *Molecular Cell* 2019, 73:107-118.e104.

2. Jonkers I, Lis JT: Getting up to speed with transcription elongation by RNA polymerase II. *Nat Rev Mol Cell Biol* 2015, 16:167-177.

3. Chen FX, Smith ER, Shilatifard A: Born to run: control of transcription elongation by RNA polymerase II. *Nature Reviews Molecular Cell Biology* 2018, 19:464-478.

4. Kamieniarz-Gdula K, Gdula MR, Panser K, Nojima T, Monks J, Wiśniewski JR, Riepsaame J, Brockdorff N, Pauli A, Proudfoot NJ: Selective Roles of Vertebrate PCF11 in Premature and Full-Length Transcript Termination. *Molecular Cell* 2019, 74:158-172.e159.

5. Gregersen LH, Mitter R, Ugalde AP, Nojima T, Proudfoot NJ, Agami R, Stewart A, Svejstrup JQ: SCAF4 and SCAF8, mRNA Anti-Terminator Proteins. *Cell* 2019.

6. Eaton JD, Davidson L, Bauer DLV, Natsume T, Kanemaki MT, West S: Xrn2 accelerates termination by RNA polymerase II, which is underpinned by CPSF73 activity. *Genes & Development* 2018, 32:127-139.

7. Chen Fei X, Woodfin Ashley R, Gardini A, Rickels Ryan A, Marshall Stacy A, Smith Edwin R, Shiekhattar R, Shilatifard A: PAF1, a Molecular Regulator of Promoter-Proximal Pausing by RNA Polymerase II. *Cell* 2015, 162:1003-1015.

8. Fujinaga K, Huang F, Peterlin BM: P-TEFb: The master regulator of transcription elongation. *Mol Cell* 2023, 83:393-403.

9. Qu X, Perez-Canadillas JM, Agrawal S, De Baecke J, Cheng H, Varani G, Moore C: The C-terminal domains of vertebrate CstF-64 and its yeast orthologue Rna15 form a new structure critical for mRNA 3'-end processing. *J Biol Chem* 2007, 282:2101-2115.

10. Caizzi L, Monteiro-Martins S, Schwalb B, Lysakovskaia K, Schmitzova J, Sawicka A, Chen Y, Lidschreiber M, Cramer P: Efficient RNA polymerase II pause release requires U2 snRNP function. *Molecular Cell* 2021, 81:1920-+.

11. Ma J, Wang M: Interplay between DNA supercoiling and transcription elongation. *Transcription* 2014, 5:e28636.

12. Kim S: Long-Distance Cooperative and Antagonistic RNA Polymerase Dynamics via DNA Supercoiling. *Biophysical Journal* 2020, 118:542a-542a.

13. Geijer ME, Zhou D, Selvam K, Steurer B, Mukherjee C, Evers B, Cugusi S, van Toorn M, van der Woude M, Janssens RC, et al: Elongation factor ELOF1 drives transcription-coupled repair and prevents genome instability. *Nature Cell Biology* 2021, 23:608-619.

14. Koga M, Hayashi M, Kaida D: Splicing inhibition decreases phosphorylation level of Ser2 in Pol II CTD. *Nucleic Acids Res* 2015, 43:8258-8267.

15. Nguyen VT, Kiss T, Michels AA, Bensaude O: 7SK small nuclear RNA binds to and inhibits the activity of CDK9/cyclin T complexes. *Nature* 2001, 414:322-325.

16. Van Nostrand EL, Freese P, Pratt GA, Wang X, Wei X, Xiao R, Blue SM, Chen J-Y, Cody NAL, Dominguez D, et al: A large-scale binding and functional map of human RNA-binding proteins. *Nature* 2020, 583:711-719.

17. Hu S, Peng L, Xu C, Wang Z, Song A, Chen FX: SPT5 stabilizes RNA polymerase II, orchestrates transcription cycles, and maintains the enhancer landscape. *Molecular Cell* 2021, 81:4425-4439.e4426.

18. Fitz J, Neumann T, Pavri R: Regulation of RNA polymerase II processivity by Spt5 is restricted to a narrow window during elongation. *The EMBO Journal* 2018, 37.

19. Castelo-Branco G, Amaral PP, Engstrom PG, Robson SC, Marques SC, Bertone P, Kouzarides T: The non-coding snRNA 7SK controls transcriptional termination, poising, and bidirectionality in embryonic stem cells. *Genome Biol* 2013, 14:R98.

20. Min IM, Waterfall JJ, Core LJ, Munroe RJ, Schimenti J, Lis JT: Regulating RNA polymerase pausing and transcription elongation in embryonic stem cells. *Genes Dev* 2011, 25:742-754.

21. Nechaev S, Adelman K: Promoter-proximal Pol II: when stalling speeds things up. *Cell Cycle* 2008, 7:1539-1544.

22. Adelman K, Lis JT: Promoter-proximal pausing of RNA polymerase II: emerging roles in metazoans. *Nature Reviews Genetics* 2012, 13:720-731.

23. Vilborg A, Passarelli Maria C, Yario Therese A, Tycowski Kazimierz T, Steitz Joan A: Widespread Inducible Transcription Downstream of Human Genes. *Molecular Cell* 2015, 59:449-461.

24. Shen S, Dean DC, Yu Z, Hornicek F, Kan Q, Duan Z: Aberrant CDK9 expression within chordoma tissues and the therapeutic potential of a selective CDK9 inhibitor LDC000067. *J Cancer* 2020, 11:132-141.

25. Zhang B, Zhong X, Sauane M, Zhao Y, Zheng ZL: Modulation of the Pol II CTD Phosphorylation Code by Rac1 and Cdc42 Small GTPases in Cultured Human Cancer Cells and Its Implication for Developing a Synthetic-Lethal Cancer Therapy. *Cells* 2020, 9.

26. Harlen KM, Churchman LS: The code and beyond: transcription regulation by the RNA polymerase II carboxy-terminal domain. *Nat Rev Mol Cell Biol* 2017, 18:263-273.

27. Ebmeier CC, Erickson B, Allen BL, Allen MA, Kim H, Fong N, Jacobsen JR, Liang K, Shilatifard A, Dowell RD, et al: Human TFIIH Kinase CDK7 Regulates Transcription-Associated Chromatin Modifications. *Cell Rep* 2017, 20:1173-1186.

28. Larochelle S, Amat R, Glover-Cutter K, Sanso M, Zhang C, Allen JJ, Shokat KM, Bentley DL, Fisher RP: Cyclin-dependent kinase control of the initiation-to-elongation switch of RNA polymerase II. *Nat Struct Mol Biol* 2012, 19:1108-1115.

29. Lu H, Yu D, Hansen AS, Ganguly S, Liu R, Heckert A, Darzacq X, Zhou Q: Phase-separation mechanism for C-terminal hyperphosphorylation of RNA polymerase II. *Nature* 2018, 558:318-323.

30. Greenleaf AL: Human CDK12 and CDK13, multi-tasking CTD kinases for the new millenium. *Transcription* 2019, 10:91-110.

31. Fan Z, Devlin JR, Hogg SJ, Doyle MA, Harrison PF, Todorovski I, Cluse LA, Knight DA, Sandow JJ, Gregory G, et al: CDK13 cooperates with CDK12 to control global RNA polymerase II processivity. *Sci Adv* 2020, 6.

32. Blazek D, Kohoutek J, Bartholomeeusen K, Johansen E, Hulinkova P, Luo Z, Cimermancic P, Ule J, Peterlin BM: The Cyclin K/Cdk12 complex maintains genomic stability via regulation of expression of DNA damage response genes. *Genes Dev* 2011, 25:2158-2172.

33. Quereda V, Bayle S, Vena F, Frydman SM, Monastyrskyi A, Roush WR, Duckett DR: Therapeutic Targeting of CDK12/CDK13 in Triple-Negative Breast Cancer. *Cancer Cell* 2019, 36:545-558 e547.

**Second round of review**

**Reviewer 1**

The authors have addressed all my questions, and I support the publication of this paper.

**Reviewer 2**

In the revision, the authors have addressed most of my concerns. Good job! I support the publication of the study. However, there is one remaining question I would like to see solved.

In the response, the authors claimed that the acute depletion of RBM22 by the addition of 5-Ph-IAA excluded the potential of an indirect effect. But this raised another possibility that 5-Ph-IAA may have adverse effects on the global Pol II transcription state. The authors should confirm that the addition of 5-Ph-IAA has no significant role in regulating WT-RBM22 expression and Pol II pausing release using regular HepG2 cells. These control experiments are easy and will improve the validity of your study.

**Reviewer 3**

The authors have carefully and satisfactorily addressed all my comments. I have no further critiques and recommend that the manuscript be accepted for publication.

**Point-by-point response**

**Reviewer #2:**

1. In the response, the authors claimed that the acute depletion of RBM22 by the addition of 5-Ph-IAA excluded the potential of an indirect effect. But this raised another possibility that 5-Ph-IAA may have adverse effects on the global Pol II transcription state. The authors should confirm that the addition of 5-Ph-IAA has no significant role in regulating WT-RBM22 expression and Pol II pausing release using regular HepG2 cells. These control experiments are easy and will improve the validity of your study.

In response to your suggestion to exclude the potential effect of 5-Ph-IAA on WT-RBM22 expression and Pol II transcription, we performed additional experiments in regular HepG2 cells. The results, now included in Fig. S2j and l, indicate no significant role of 5-Ph-IAA in regulating WT-RBM22 expression and Pol II pausing release. These findings have been incorporated into the revised manuscript.

**Third round of review**

**Reviewer 2**

The revision has addressed my concerns, and the study has further confirmed the good findings identified in the authors’ Cell paper (2019). I am sure this study provides an updated insight into functions of the general splicing machinery. The authors may consider evaluating other components of the splicing complex next.
